# Soil organic carbon thresholds control fertilizer effects on carbon accrual in croplands worldwide

**DOI:** 10.1038/s41467-025-57981-6

**Published:** 2025-03-27

**Authors:** Jun Ling, Jennifer A. J. Dungait, Manuel Delgado-Baquerizo, Zhenling Cui, Ranran Zhou, Wushuai Zhang, Qiang Gao, Yuanxue Chen, Shanchao Yue, Yakov Kuzyakov, Fusuo Zhang, Xinping Chen, Jing Tian

**Affiliations:** 1 State Key Laboratory of Nutrient Use and Management, College of Resources and Environmental Sciences, China Agricultural University, 100193 Beijing, PR China; 2 Geography, Faculty of Environment, Science and Economy, University of Exeter, Rennes Drive, Exeter, EX4 4RJ UK; 3 Carbon Management Centre, SRUC-Scotland’s Rural College, Edinburgh, EH9 3JG UK; 4 Laboratorio de Biodiversidad y Funcionamiento Ecosistémico, Instituto de Recursos Naturales y Agrobiología de Sevilla (IRNAS), CSIC, 41012 Sevilla, Spain; 5 College of Resources and Environment, Academy of Agricultural Science, Interdisciplinary Research Center for Agriculture Green Development in Yangtze River Basin, Southwest University, 400715 Chongqing, China; 6 College of Resources and Environment, Jilin Agricultural University, 130118 Changchun, China; 7 College of Resources and Environment, Sichuan Agricultural University, 611134 Chengdu, China; 8 Institute of Eco-Environment and Industrial Technology, Shanxi Agricultural University, 030031 Taiyuan, China; 9 Department of Soil Science of Temperate Ecosystems, University of Göttingen, 37077 Göttingen, Germany; 10 Bioeconomy Research Institute, Vytautas Magnus University, Agriculture Academy, Kaunas Reg., Lithuania

**Keywords:** Carbon cycle, Agroecology

## Abstract

Initiatives to restore soil fertility and mitigate global warming rely on rebuilding soil organic carbon (SOC). Nitrogen (N) fertilization is crucial for crop yields but affects SOC unpredictably due to varying responses of particulate organic carbon (POC) and mineral-associated organic carbon (MAOC) pools to initial SOC levels. To clarify these effects, here, by combining a global meta-analysis with continental-scale field experiments, we determine that an initial SOC threshold of 15 g C kg^−1^ controls the effect of N fertilization on POC and MAOC. In SOC-poor soils (< 15 g C kg^−1^), N fertilizer increases plant-derived C inputs and promotes soil aggregation, favouring POC accumulation. Conversely, in SOC-rich soils, N fertilizer stimulates microbial metabolic efficiency, leading to larger necromass production and stabilization by mineral protection, observed as more pronounced MAOC accrual. Our findings reveal how SOC thresholds shape the response of active and stable carbon pools to N fertilization, with consequences for SOC accrual in cropland soils globally.

## Abstract (web summary)

The study revealed how nitrogen fertilization affects fast and slow turnover pools of soil organic carbon differently. The authors found labile particulate organic carbon increased if SOC was below 1.5%, while stable mineral associated organic carbon increased above this critical threshold.

## Introduction

Arable lands have lost up to half of organic carbon (C) stocks since the Industrial Revolution, with a cumulative depletion nearing 62 Pg C, limiting soil fertility and threatening food security^1–3^. Soils depleted in organic C by intensive agriculture are often regarded as a potential global C sink. Building SOC in croplands is a challenging task which is, however, essential to restore soil functionality and ensure food production^4,5^. Nitrogen (N) fertilization is essential for agricultural productivity to meet the human demand for food^6^, but can strongly affect SOC dynamics^7,8^. In general, N fertilization facilitates the production of plant biomass, increasing C inputs to soil that should increase SOC^9^. However, variable responses of SOC accrual to N addition are reported, ranging from decreases^10^ to no change^11^ to increases^12^. The wide variation in the response of SOC to N fertilization has important implications for safeguarding soils to meet both Sustainable Development Goal (SDG) 2 (Zero Hunger) and SDG 13 (Climate Action) under current circumstances that require increased food production, optimizing fertilizer use and increasing C capture/storage^13,14^. Understanding anthropogenic impacts on SOC is assisted by considering the total SOC pool as a combination of particulate organic carbon (POC) and mineral-associated organic carbon (MAOC) that differ broadly in their chemical composition, turnover, and ecological functioning^15–17^. Where N availability to plants is limited, N-induced increases plant C inputs into soils is a necessary first step to SOC regeneration^18,19^. However, simply increasing the precursor materials is not sufficient to achieve long-term soil C sequestration: protection of the newly formed SOC from decomposition is crucial to ensure its long-term persistence. Therefore, understanding the mechanisms underlying variation in the response of SOC pools and its functionality to N fertilizer is now critical.

Initial SOC content has recently emerged as an important factor that modifies the responses of POC and MAOC pools to N fertilization via direct effects on microbial metabolism and indirect effects on soil physico-chemical and mineralogical traits^20,21^. Ecological stoichiometry theory (EST) predicts that the growth and reproduction of microbial communities are driven by microbial demand and resource supplies^22^. Fertilizer N inputs to SOC-poor soils may cause an stoichiometric imbalance (low C: high N) between microbial demands and soil resource availability, leading to the reallocation of a greater proportion of the substrate to maintenance metabolism^21,23^, observed as elevated microbial respiration^24^. In contrast, fertilizer N inputs to SOC-rich soil satisfy microbial nutrient demand^25^, stimulate microbial activity by providing nutrients and energy to both accelerate decomposition of plant residues and invest in the synthesis of microbial biomass and, subsequently, necromass production^26^. The accumulation of microbial necromass C and its stabilization as MAOC is also contingent on wider soil geochemical properties that could undergo significant changes that might be substantially altered by N fertilizer application^27^. SOC-rich soils buffer N-induced acidification due to the cation exchange capacity (CEC) of soil organic matter, at the cost of increased leaching of base cations (e.g., Ca^2+^ and Mg^2+^) and greater solubility of hydrolyzed cations (e.g., Fe^3+^ and Al^3+^)^28^. Compared to finer-textured (clayey) soils, coarse-textured (sandy) soils tend to be C-poor with smaller CEC and thus poorer buffering capacity against cation leaching caused by N fertilizer use^28^. Variations in mineral cation chemistry influence adsorption and co-precipitation reactions with organic compounds in soils, including those derived from microbial necromass, causing variation in the effect of N fertilizer on MAOC formation^20,29^. Additionally, N fertilizer may strengthen macroaggregate formation by stimulating root and mycorrhizal growth, which, together with plant-derived C, contribute to POC pool and its stabilization as intra-aggregate POC^20,29^. Therefore, the relationship between initial SOC and functional pools responses to N fertilization incorporates myriad variations of complex interactions between multiple mechanistic drivers, including microbial metabolic and geochemical factors that affect the balance between POC and MAOC pools. Assessing the relationship between initial SOC and C sequestration in response to fertilization is critical to better forecast the changes in POC and MAOC pools in a changing world.

**Fig. 1:**
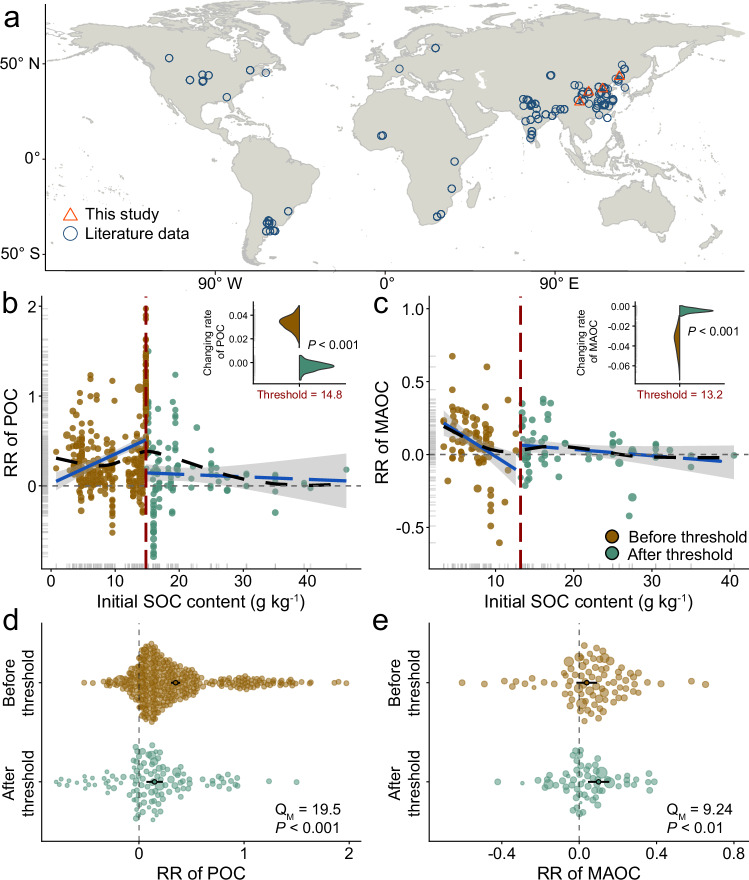
Global distribution of study sites and the interactions between nitrogen fertilization and initial soil organic carbon (SOC)on particulate organic carbon and mineral-associated organic carbon (POC and MAOC) in global cultivated upland soil. **a** Global distribution of study sites. **b**–**e** nonlinear responses of POC and MAOC attributes to initial soil organic carbon content. In **b**, **c** black long-dashed lines represent the smoothed trend fitted by a generalized additive model and blue solid lines represent the linear fits at both sides of each threshold. Inset red numbers and the vertical dashed lines describe the initial SOC identified. Violin diagrams show bootstrapped slopes or values of the predicted fitted trend at the threshold of the two regressions existing at each side of the threshold (green: before the threshold; brown, after the threshold). Significant Q_M_ (between-group heterogeneity) indicates that the response ratios differ between before and after the threshold of SOC (*P* < 0.05). Basemap in (**a**) from Natural Earth (https://www.naturalearthdata.com/). The coastline boundaries in the map are derived from the GSHHG database (https://www.ngdc.noaa.gov/mgg/shorelines/data/gshhg/latest/). Source data are provided as a Source Data file.

Herein, we combined a global meta-analysis with new data from decades-long continental-scale N fertilization field experiments in four sites across China (Fig. 1a and Supplementary Fig. 1) to investigate how initial SOC content may interact with N fertilization to influence the SOC accrual of global cropland soils. We hypothesized: 1) N fertilization promotes plant productivity and macroaggregate formation, and thereby increases POC accumulation and stabilization; 2) an imbalance between resource stoichiometry and microbial nutrient demand in SOC-poor soils hinders the formation of microbial necromass C and the decomposition of POC, weakening MAOC accrual; 3) Greater microbial metabolic efficiency and necromass production coupled with mineral protection, all of which increase MAOC in SOC-rich soils under increased N availability. Here, we address these hypotheses using two comprehensive approaches: a global meta-analysis of POC and MAOC contents from 118 fertilization experiments, and the analysis of four coordinated long-term field experiments with N fertilization to further evaluate the underlying mechanisms, including plant input, microbial transformation, and protection by aggregate and minerals. We identified a critical soil carbon threshold (i.e., 15 g kg^−1^ SOC) controlling the influence of N fertilization on C sequestration. Below this C threshold, the sequestration in response to fertilization tends towards the formation of POC derived directly from plant C, while surpassing this point shifts the balance towards MAOC accumulation derived from soil microorganisms. By identifying how specific SOC components respond to N fertilization and how carbon thresholds influence these responses through plant-microbe-soil interactions, this study paves the way for more targeted, effective soil management strategies that can bolster global food security while combating climate change.

## Results

### Fertilizer impacts on POC and MAOC depend on initial SOC in global croplands

To determine whether initial SOC content affects global changes of POC and MAOC in response to N fertilization, we compiled a database including 609 paired observations of POC and MAOC changes from 118 field sites worldwide (Fig. 1a), covering a very wide range of initial SOC content (g kg^−1^; 0.79 to 46.1). Overall, POC had 5.0% negative, 41% neutral and 54% positive responses to N addition (average effect size = 0.22, *P* = 0.02); while MAOC had 8.0% negative, 64% neutral and 28% positive responses to N addition (average effect size = 0.06, *P* = 0.02; Supplementary Fig. 2). To help explain changes in POC and MAOC responses to N fertilizer and drivers underlying, we considered 8 climatic, management and edaphic variables as moderators. Variance partition analysis showed that the relative importance of edaphic properties and management practices were higher than that of climate conditions (Supplementary Fig. 3). Among them, significant predictors in our model included individual effects of initial SOC, N fertilizer and their interactions (*P* < 0.01; Supplementary Fig. 4), suggesting that the N fertilization on POC and MAOC was modulated by initial SOC levels across different regions. The result showed that the nonlinear model was more suitable for POC and MAOC to initial C content (Supplementary Fig. 5 and Supplementary Table 1), which indicated that there were SOC thresholds where abrupt changes occurred.

Further threshold analysis assessed the responses of POC and MAOC to various initial SOC contents, identifying specific SOC levels at which their responses exhibited abrupt changes. By comparing six nonlinear models, we found that the N-induced POC and MAOC changes responded in a stegmented manner to increases in initial SOC content (Supplementary Table 1). Specifically, abrupt changes in POC and MAOC occurred over a small range of initial C content of ~13–15 g kg^−1^ (Fig. 1b, c). A sharp rise in POC was detected before an initial C content of 14.8 g kg^−1^ and then slightly decreased (Fig. 1b). However, at low levels of initial C, MAOC decreased abruptly. Once a 13.2 g kg^−1^ SOC threshold was crossed, MAOC slightly decreased as SOC increased (Fig. 1c). This notion is further supported by heterogeneity analysis, demonstrating clear differences in the POC and MAOC responses before and after reaching the SOC threshold under N addition (Q_M_ = 19.5, *P* < 0.001 for POC; Q_M_ = 9.24, *P* < 0.001 for MAOC; Fig. 1d, e). Note that the POC increase under N fertilization was more important before the threshold than afterward, whereas the MAOC response followed the opposite trend.

### Divergent responses of POC and MAOC to a decade of N addition in experimental sites

**Fig. 2:**
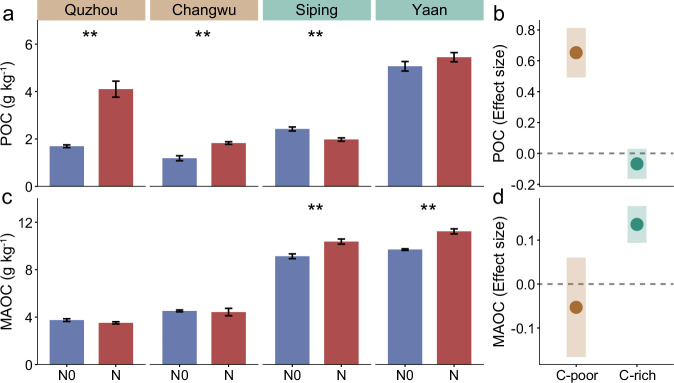
Effects of long-term nitrogen fertilization on carbon pools across four field experiments, with results categorized into C-poor and C-rich soils from four sites. **a, c** Effects of N fertilization on POC and MAOC across four field experiments. Asterisk indicate a statistical significance of the effect based on non-parametric Wilcoxon test (*P* < 0.05) using the Benjamini and Hochberg (BH) method (*n* = 4 for N0, *n* = 8 for N). **b**, **d** Effects of N fertilization on POC and MAOC in both C-poor and rich soils. The estimated effect sizes are regression coefficients based on rescaled response variables (with zero mean and unit standard deviation) in the linear mixed-effects model. The points and shades represent mean ± s.e.m. of effect sizes in B and D. Statistical significance is based on Wald type II χ² tests, and 95% CI not overlapping zero indicated significant. POC: particulate organic carbon; MAOC: mineral-associated organic carbon. N0: control (No N addition); N: N addition (results of combining optimized N addition and high N addition). C-poor: combining QZ and CW results; C-rich: combining SP and YA results. Source data are provided as a Source Data file.

To disentangle the potential mechanisms behind how initial SOC content influenced N-induced POC and MAOC changes, we collected soil samples from four coordinated long-running field N fertilizer experiments network (Supplementary Fig. 1). These experiments were located in four cropland sites with different initial SOC contents: Quzhou (QZ, 9.49 g kg^−1^), Changwu (CW, 9.51 g kg^−1^), Siping (SP, 16.3 g kg^−1^) and Yaan (YA, 17.4 g kg^−1^). A decade of N fertilizer affected SOC fractions across four sites (*P* < 0.001; Supplementary Table 2). The optimized and high N treatments (OPT and HN) increased POC content at QZ and CW (*P* < 0.01), while increased MAOC content at the SP and YA (*P* < 0.01; Supplementary Fig. 6). Given the consistent results from both OPT and HN, we merged these findings under ‘N fertilizer effects’ in our analysis, enhancing interpretability for readers. Interestingly, the responses of POC and MAOC contents to N fertilizer divided the four sites into two categories: POC accumulation response and MAOC accumulation response (Fig. 2). Along with individual effects of N fertilizer and initial SOC content, there were interactive effects of N × Initial C content on SOC functional pools (*P* < 0.001; Supplementary Table 2), suggesting that changes of POC and MAOC contents to N addition strongly depended on initial SOC content. Based on the results of the meta-analysis, the initial SOC threshold for the mutation of POC and MAOC was determined to be between 13–15 g kg^−1^. The initial SOC content of QZ and CW was found to be less than 13 g kg^−1^, while that of SP and YA was greater than 15 g kg^−1^. Consequently, the soils at the four points were classified into two categories: C-poor soils and C-rich soils. Notably, a strong contrast exists in the changes of POC and MAOC contents between C-poor and C-rich soils (Fig. 2b, d). Further analysis revealed that N fertilizer had positive effect on POC content in C-poor soils, demonstrating an average increase of 105% compared to the soil without N fertilization (N0) (*P* < 0.001; Fig. 2a, b). However, N addition increased MAOC content by 15% in C-rich soils relative to N0 (*P* < 0.001; Fig. 2c, d).

### Plant-microbe-soil matrix interactions underpin contrasting responses of POC and MAOC to N fertilization in C-poor and C-rich soils

**Fig. 3:**
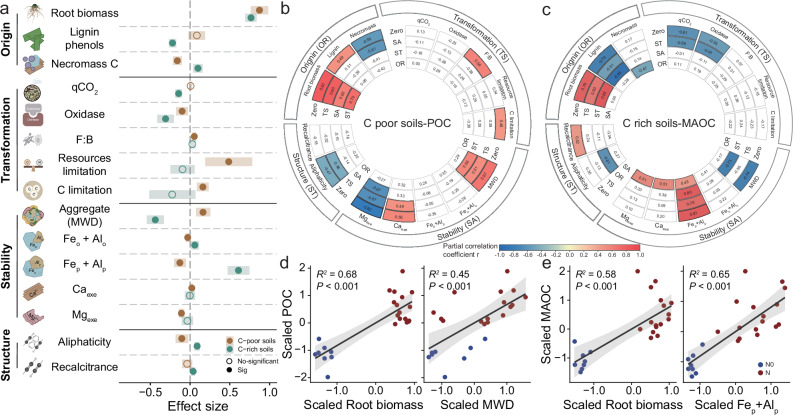
Effects of long-term nitrogen fertilization on the origin, transformation, and stability of carbon pools, as well as key indicators of carbon pool dynamics. **a** Effects of N fertilization on SOC origin (root biomass, lignin phenols and microbial necromass C), microbial transformation (qCO_2_, oxidase, F:B, resource limitation and C limitation), SOC physical and biochemical stability (aggregate stability, Fe_o _+ Al_o_, Fe_p _+ Al_p_, Ca_exe_ and Mg_exe_) and structure (Aliphaticity and Recalcitrance) between C-poor and C-rich soils and their difference. The estimated effect sizes are regression coefficients based on rescaled response variables (with zero mean and 95% confidence intervals) in the linear mixed-effects model. The points and shades represent mean ± s.e.m. of effect sizes. Statistical significance is based on Wald type II χ² tests, and 95% CI not overlapping zero indicated significant. Filled circles represent significant effects by N fertilizer (*P* < 0.05). **b**, **c** Partial correlations between POC of C-poor soils and MAOC of C-rich soils with four types of factors (SOC origin (OR), microbial transformation (TS), SOC physical and mineral stability (SA) and structure (ST)). The outermost circle represents the factors (i.e., OR, TS, SA and ST) under examination for their correlations with POC or MAOC. The color of the fan shapes signifies the strength and direction of the correlation, with a black frame indicating statistical significance at the 0.05 level. Distinctions in color between the zero-order (Zero) and controlled factors indicate the extent of dependency of the correlation between POC or MAOC and the examined factor on the controlled variable (no change in circle color between the controlled factor and zero-order = no dependency; a decrease/increase in circle color intensity = loss/gain of correlation). **d**, **e** Correlations between POC in C-poor soils and MAOC in C-rich soils with key predictors. Solid line in each panel shows significant model fit using linear regression (*P* < 0.05), and the shading around the fitted line represents the 95% confidence intervals. qCO_2_ (μg CO_2_-C μg^−1^ MBC h^−1^): metabolic quotient; F:B: fungal-bacterial biomass ratio; MWD: aggregates mean weight diameter; Fe_o _+ Al_o_: po_o_rly crystalline Fe/Al oxides; Fe_p _+ Al_p_: organically complexed Fe/Al oxides; Ca_exe_ and Mg_exe_: the molar ratios of exchangeable Ca^2+^ and Mg^2+^. Aliphaticity: the ratio of the alkyl region divided by the O-alkyl region; Recalcitrance: the ratio of (alkyl + aromatic C)/ (O-alkyl + carboxyl C). POC: particulate organic carbon; MAOC: mineral-associated organic carbon. N0: control (No N addition); N: N addition (results of combining optimized N addition and high N addition). C-poor soils: combining Quzhou and Changwu results; C-rich soils: combining Siping and Yaan results. Some elements in (**a**) were created in BioRender. B, A. (2025) https://BioRender.com/b56q841. Source data are provided as a Source Data file.

To investigate the underlying mechanisms in governing the dynamics of POC and MAOC contents in response to N fertilizer, we analyzed a set of variables associated with C pools, including plant and microbial biomarkers, soil microbial properties, aggregate and mineral protection as well as SOC chemical composition. As compared to N0, N fertilizer increased root biomass in C-poor (~148%) and C-rich (~115%) soils, respectively (*P* < 0.001; Fig. 3a and Supplementary Fig. 7). N fertilizer altered both lignin phenols and necromass C, depending on initial C content (*P* < 0.001; Supplementary Table 2). As compared to N0, N fertilizer increased lignin phenols by 18.4% (*P* = 0.121) but decreased microbial necromass C in C-poor soils (*P* < 0.001; Fig. 3a and Supplementary Fig. 8). Lignin phenols and microbial necromass C had contrasting responses to N fertilizer in C-rich soils: lignin phenols exhibited a negative response, while microbial necromass C showed a positive response (*P* < 0.01; Fig. 3a and Supplementary Fig. 8). Consequently, N fertilizer increased microbial necromass C by 10.2% compared by N0, confirming the accumulation of MAOC in C-rich soils (*P* < 0.01; Fig. 3a).

Microbial properties associated with C transformation exhibited pronounced contrastive effects in response to N fertilizer between the C-poor and C-rich soils (*P* < 0.05; Fig. 3a and Supplementary Table 2). Specifically, N fertilizer aggregated microbial C limitation, relative to N0 in C-poor soils (*P* < 0.05; Fig. 3a and Supplementary Fig. 9). By contrast, N fertilizer alleviated microbial C and resources limitation, leading to 13.6% decrease of microbial metabolic quotient (qCO_2_) in C-rich soils (*P* < 0.001; Fig. 3a and Supplementary Fig. 10), indicating a more efficient microbial growth. Additionally, N addition reduced the oxidases (phenol oxidase and peroxidase) by 26.1% relative to N0 (*P* < 0.01; Fig. 3a and Supplementary Table. 3). We then measured the mean weight diameter (MWD) of aggregates and the content of exchangeable cations to quantify SOC protection by aggregates and minerals, respectively. The fertilization effects on aggregate stability and mineral protection often depended on initial SOC (*P* < 0.05; Supplementary Table 2). N fertilizer had a positive effect on MWD in C-poor soils (20.7% increase), but decreased in C-rich soils (*P* < 0.001; Fig. 3a and Supplementary Fig. 11). In contrast, N fertilizer enhanced the content of Fe and Al oxides in C-rich soils, thereby augmenting the soil’s mineral protection capacity (*P* < 0.05; Fig. 3a and Supplementary Fig. 11). ^13^C-NMR spectroscopy analysis further showed that N fertilizer increased aliphaticity and recalcitrance from 3.0% to 10.0% in C-rich soils, indicating that N may contribute to the stabilization of organic carbon in such soils (*P* < 0.05; Fig. 3a and Supplementary Table 4).

Partial correlation analysis showed that, in C-poor soils, despite the significant correlations between POC and the four types of factors (i.e., SOC origin, microbial transformation, stability and structure; zero-order in Fig. 3b), the correlation coefficients of POC and Mg_exe_ decreased by 203% after controlling for the SOC origin (Fig. 3b). By contrast, root biomass and MWD were always closely related to the POC in C-poor soils (*P* < 0.001; Fig. 3d), even after controlling for the other three or two types of factors. Specifically, increasing root biomass and MWD was coupled with increasing POC (R^2^ = 0.68, *P* < 0.001; R^2^ = 0.45, *P* < 0.001) in C-poor soils (Fig. 3d). In C-rich soils, after controlling SOC stability, the correlation coefficients between MAOC and C origin (characterized by plant C input and biomarkers) became insignificant (Fig. 3c). Moreover, the root biomass and Fe_p _+ Al_p_ consistently increased with the MAOC (Fig. 3e; R^2^ = 0.58, *P* < 0.001; R^2^ = 0.65, *P* < 0.001). Accordingly, plant C input was the single control in all soils, exerting strong positive effects on POC and MAOC. Physical protection and mineral protection were strongly associated with POC accumulation in C-poor soils and MAOC accumulation in C-rich soils, respectively (Fig. 3).

### Changes in global farmland POC and MAOC under current N fertilization scenarios

**Fig. 4:**
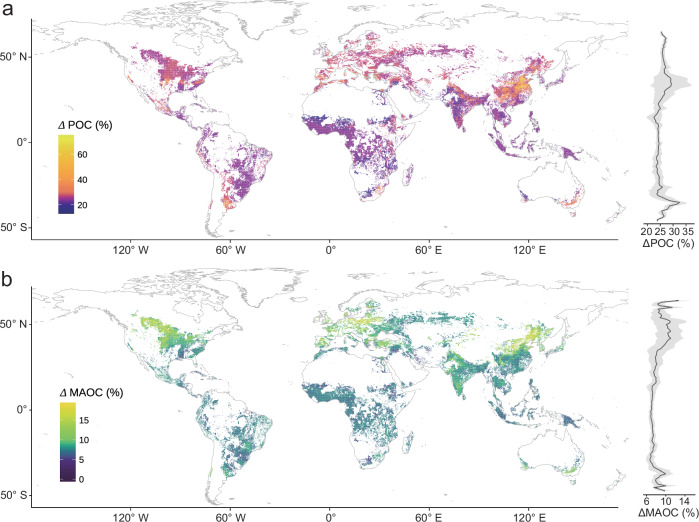
Maps of predicted spatial distribution of the impacts of current nitrogen fertilization rates on POC and MAOC changes (%) across global cultivated uplands and its latitudinal pattern, respectively. **a** Global map of changes in particulate organic carbon (POC) and its latitudinal distribution. **b** Global map of changes in mineral-associated organic carbon (MAOC) changes and its latitudinal distribution. The inset on the right-hand side shows the latitudinal averages (black line) and standard deviations (gray shading) of POC or MAOC. “Δ” represents the percentage change in POC or MAOC under the current fertilization level compared to the unfertilized condition. Basemap in (**a**) from Natural Earth (https://www.naturalearthdata.com/). The coastline boundaries in the map are derived from the GSHHG database (https://www.ngdc.noaa.gov/mgg/shorelines/data/gshhg/latest/). Source data are provided as a Source Data file.

Briefly, after the standard process of model metric evaluation (Supplementary Table 5), the optimized gradient-boosting decision trees model was chosen as the optimal model for POC and MAOC. We then applied our predictive model to scale up the site-level observations across the globe at a ~ 10-km resolution to quantify changes in POC and MAOC under current N fertilizer application rate relative to a no-fertilization scenario. Over the past decade, our global estimates indicate that the application of N fertilizer has led to an average increase in POC and MAOC by 26% and 8.3%, respectively (Fig. 4a and b). Moreover, we partitioned worldwide farmland into two categories based on SOC thresholds (SOC threshold of our meta-analysis data): C-poor and C-rich farmland. N fertilizer application increased POC by 26% in C-poor soils and 22% in C-rich soils, compared to unfertilized soils (Supplementary Fig. 12). However, the MAOC increase induced by fertilization was 7% in C-poor soils and 8% in C-rich soils (Supplementary Fig. 12). Across ecozones, we found that the beneficial impacts of N fertilizer on POC were greatest in the subtropical (27%), followed by temperate (26%) and boreal (25%), and least in tropical zones (24%; Supplementary Fig. 13). Specifically, substantial POC accrual due to fertilization is most evident in east Asia and north America. In contrast, the response of POC to fertilization in Africa was minimal or neutral. Our study revealed a relatively uniform enhancement in MAOC, induced by N fertilizer, across diverse ecozones, with increases varying between 7% and 10 %. The temperate zone observed the most significant rise, whereas the tropical zone exhibited the smallest increase (Supplementary Fig. 13).

## Discussion

**Fig. 5:**
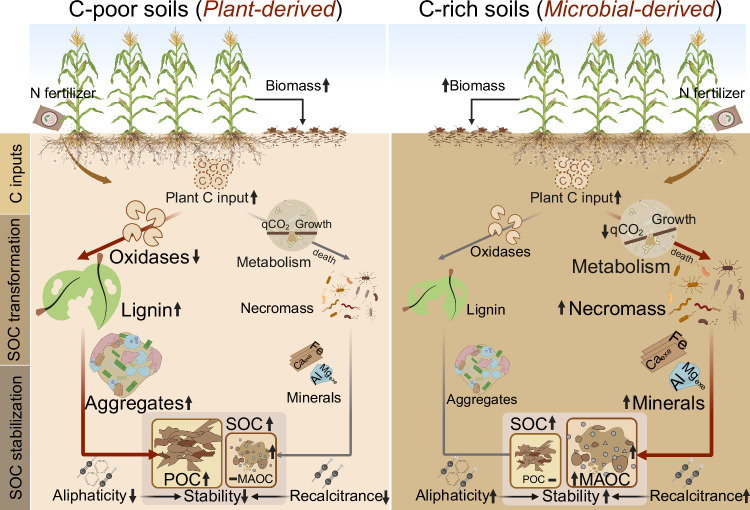
Conceptual diagram illustrating the N fertilization effects on SOC and functional pools (POC and MAOC) as well as their regulation mechanisms through C input origins, microbial transformation, and physical and chemical stabilization in C-poor and C-rich soils. Evidently, in C-poor soils with low SOC content, N fertilization facilitates the POC retention and increased SOC content, primarily through its influence on plant-derived C and protection within aggregates. Conversely, in C-rich soils with high SOC content, N fertilizer stimulated the sequestration of MAOC and increased SOC by more efficient microbial C cycling (high metabolic efficiency and pump efficiency) and strong mineral protection mechanisms. The red path represents the primary mechanism by which nitrogen fertilizer affects carbon components under the current conditions. The direction of the arrow indicates the change in the variable (upward/increase; downward/decrease), while a horizontal line represents no change. Some elements of this figure were created in BioRender. B, A. (2025) https://BioRender.com/i51j856.

Restoring SOC in agricultural land is pivotal for enhancing sustainable agricultural production and offering a viable strategy to mitigate global warming^3^. Soil carbon is not a uniform entity, differentiating it into POC and MAOC can help understand SOC formation and accrual, and thus provide more precise guidance for managers and policymakers. However, current assessments of the impact of N fertilizers on POC and MAOC depending on soil fertility levels are insufficient, which impedes the advancement of sustainable agriculture and the optimization of carbon sequestration benefits. Combining a global meta-analysis of published literature and experimental evidence from four experiments, we identified a critical soil C threshold (i.e., 1.5% SOC) controlling the influence of N fertilization on C accrual. Below this crucial soil C threshold, C accrual in response to fertilization is inclined towards the formation of POC, while surpassing this point shifts the balance towards more MAOC accumulation (Fig. 1d, e). Initial SOC content affects N-induced changes in POC and MAOC through direct effects on plant C input and indirect effects on microbial transformation, physical and mineral protection (Fig. 5).

Utilizing systematic research and diverse viewpoints, this study delves into the mechanisms behind the formation of SOC functional components mediated by N fertilizer and initial C, systematically analyzing the sources, transformation processes, and stability of these components. In agricultural systems, crop yields are often increased by N fertilization, and both above- and belowground biomass and root-derived C inputs are generally larger^18^. In C-poor soils, the increased POC was evidenced by larger root biomass and lignin phenols following N fertilization (Fig. 3a and d), in agreement with hypothesis (1). Typically, an increase in plant C input catalyzes a corresponding upsurge in enzyme activities, attributable to the enriched availability of substrates that “fuel” microbial processes^30^. In the present study, soil oxidase activities decreased slightly with N addition in C-poor soils (Supplementary Table 3), which is consistent with a recent meta-analysis that N addition suppressed the activity of lignin-decomposing enzymes^31^, resulting in the accrual of plant residues as lignin macromolecules^32^. Other than extracellular enzymes, microbial composition and classification also play a role in mediating lignin breakdown and subsequent POC formation. Fungal biomass and F:B were increased under N fertilization (Fig. 3a; Supplementary Fig. 14), suggesting that microbial communities undergo a transition towards a fungus-dominated ecosystem to raise the efficient breakdown of lignin^33^, also observed by Man et al. ^34^. Furthermore, according to EST, when N is abundant in C-poor soils, an imbalance between resource availability and microbial nutrient needs can lead to stoichiometric imbalance^23,25^. This imbalance hinders the transformation of POC to MAOC because microbes, the primary mediators in synthesizing unstable C into MAOC, lead to higher POC retention^19,35^. In addition to the aforementioned biological factors, soil aggregates play a pivotal role in the physical protection of POC (Fig. 3b, d)^36^. We found that N fertilization increased the stability of soil aggregates (Fig. 3a), which may be a result of increased plant litter, rhizodeposition and fungi biomass. The residues of litter and rhizodeposition have the capacity to bind with fungal hyphae and soil particles, leading to the formation of macroaggregates^37,38^. Soil texture, alongside aggregate formation, is a key factor influencing C accumulation^39–41^. Our meta-analysis indicated that clay and silt content strongly impact POC and MAOC accumulation (Supplementary Fig. 4). Heterogeneity analysis revealed that the effect size of POC is larger in clayey soils than clay loamy soils (Supplementary Fig. 15), as clay has more reactive sites for C adsorption^42^. Overall, the net beneficial impact of N addition on POC in C-poor soils is dependent upon interactions among increased root biomass and lignin, alterations in extracellular enzyme activities, and robust soil aggregate protection (Fig. 5).

While global consensus on increasing C sequestration, especially slow-turnover pools (e.g., MAOC), has gained importance over the past decade, the direction and magnitude of changes in MAOC in response to N remain understudied^9,12,43^. Here, we observed that N fertilization had particularly positive effect on MAOC in C-rich soils (Fig. 2c, d; Supplementary Fig. 6). We also observed that N fertilization increased newly formed microbial necromass, particularly facilitating the contribution of fungal necromass to the stable C pool (Fig. 3a and Supplementary Fig. 8). This indicates a shift in the trade-off between labile and stable SOC pools under N fertilization, which can be due to multiple overlapping mechanisms. First, the increase in root biomass and rhizodeposition induced by N fertilizer increased the availability of simple carbohydrates, which are more efficiently utilized to form microbial biomass and subsequently transformed into MAOC^44,45^. Second, aligning with our second hypothesis, increased N availability can mitigate imbalances between microbial community and available resources in C-rich soils (> 1.5% SOC), thereby alleviating microbial nutrient limitations and subsequently increasing necromass C production^23,45^. Our study is comparable with previous reports that microorganisms are more C-limited and exhibit reduced metabolic efficiency, in soils with low soil SOC content (< 1%), as evidenced by elevated qCO_2_ values^21,46^. We also determined lower qCO_2_ values under N addition in C-rich soils (Fig. 3a). Of course, microbial stoichiometric balance is influenced by variations in both C content and soil nutrient availability^22^. While N fertilizer application rate can be a key factor influencing microbial metabolic responses to fertilization^47^, the responses are not dependent on the amount of N applied^48^, which aligns with our results (Supplementary Fig. 9). Third, analysis of extracellular enzymes provides evidence that elevated phenol oxidase and peroxidase activities may increase the potential of microbes to acquire C from SOC pools with slower turnover rates^31,49^. The notable reduction in oxidase activities observed in this study may have shifted the microbial C acquisition strategies, ultimately contributing to an augmentation of MAOC under N addition (Fig. 3c, e). Finally, the adsorption of organic compounds onto mineral surfaces is a hallmark of C-rich soils, rendering these soils particularly susceptible to acidification driven by N fertilization^50^. N fertilization had significant positive effect on mineral protection in C-rich soils in this study, primarily caused by increasing content of organic-aluminum/iron complexes (Fe_p_ and Al_p_; Fig. 3a). Specifically, N-induced acidification increased overall MAOC as a consequence of heightened mineral cations solubility and the subsequent formation of organo-metal complexes (Supplementary Fig. 11)^27^. Our study demonstrates that C-rich soils that contain larger clay and silt fractions (such as those from SP and YA) with enhanced water-holding capacity support microbial biomass production and thereby microbial necromass formation^51^. The large surface area of fine soil particles provides more mineral surfaces and reactive sites to stabilize microbial-derived C and promote MAOC accumulation^42,52^. Additionally, the augmented contribution of stable C to the SOC pool was evidenced by an 4.1% - 8.7% increase in aliphaticity following N addition (Supplementary Table 4), since aliphaticity represents a comprehensive index for assessing chemically ‘recalcitrant’ compounds^53^. Overall, N-induced MAOC accumulation in C-rich soils mainly resulted from accumulated necromass and increased stabilization on minerals (Figs. 2d and 3a). Our results together underscore the crucial role of root biomass, microbial properties and minerals as key predictors influencing the observed accumulation in MAOC levels following N addition in C-rich soils.

The ability to predict C pools and its functionality changes under current N fertilization can guide the optimal fertilization application of cropland-based climate solutions. Recent studies have forecasted a wide range of SOC responses to inorganic fertilizers, which differ in magnitude and sign^54^. The uncertainty is, in part, attributed to the allocation of C into model pools that lack distinct biophysical definitions and exhibit varying sensitivities to N addition.

Based on our findings, the application of N fertilizer promoted the accrual of MAOC in C-rich arable soils (Supplementary Fig. 12), and could help combat the projected soil C losses from croplands to meet soil C sequestration targets. Several existing meta-analysis studies have also shown that fertilization slightly promoted MAOC accumulation^9,12,43,55^. However, this strategy should be implemented with caution to mitigate potential global risks to the economy, environment, and public health^56^. For instance, the benefits of increasing the MAOC sink may be partly offset by increased N_2_O emissions, fossil energy and the associated greenhouse gas emissions during fertilizer manufacture^57,58^. Considering the greenhouse gas emissions throughout the entire life cycle of N fertilizer, the global warming potential of fertilized soil remains greater than that of unfertilized soil^58^. This trade-off is often necessitated by the imperative of ensuring food security. Developing a comprehensive understanding of N fertilizer-induced C changes is essential to accurately forecast the fate of SOC in a warmer world, for maximizing agricultural ability to mitigate climate change and provide additional ecosystem services. Our global forecasts indicated that fertilization leads to a significantly greater increase in POC in C-poor soils compared to C-rich soils (Fig. 4a). Yet, due to POC’s rapid turnover and vulnerability—lacking protection from microaggregates and minerals—this retention does not translate into long-term SOC gains^59^, particularly under global warming conditions^60^. Moreover, warming that increase microbial activity will likely accelerate POC decomposition, as microbes process available C sources, potentially leading to significant carbon loss in an amplified manner^42^. This exacerbation leads to elevated emissions of greenhouse gases, thereby creating a positive feedback loop that further intensifies the warming of our planet’s climate. Although fertilization has been observed to increase C pools (Fig. 4), caution is necessary when assessing its future potential, particularly in areas with overfertilization and high fertility, where the outcomes are often suboptimal^54^. While our analysis provides a global estimate of N fertilization-induced changes in C pools over the past decade, several limitations must be acknowledged. First, our global assessment, based on meta-analyzes with non-standardized experimental and sampling protocols across studies, restricts model outputs to correlative interpretations rather than establishing causality. To address this limitation, future efforts should prioritize standardized multi-region N fertilizer trials that integrate plot-scale data with causal inference methods. Such frameworks would elucidate the causal relationships underlying how N fertilizer mediates plant-microbial-environmental feedbacks governing C pools dynamics. Second, sparse data coverage in Europe, Canada, and Russia constrains model predictability and accuracy in these regions, particularly given their high-latitude locations and heightened susceptibility to climate change impacts. Addressing this limitation will require concerted efforts from the scientific community to perform large-scale, networked experiments across continents. Third, our estimates rely on interpolated global datasets with coarse spatiotemporal resolution (Supplementary Table 5), as many plot-level metadata are unavailable. To reduce uncertainties in such datasets, further efforts to enhance data quality and resolution are necessary. Despite these limitations, the comprehensive nature of our study, which incorporates a diverse range of data sources and advanced analytical methods, provides a robust estimation of N fertilizer-induced C pools changes.

In summary, our work provides new insights into the benefits of N fertilizer application for SOC accrual in global cropping systems. We discern two distinct mechanisms of global soil C accumulation following N fertilizer across an initial C threshold of ~15 g kg^−1^: in C-poor soils (SOC < 15 g kg^−1^), accumulation is primarily driven by increased plant inputs and aggregate protection promoting POC; conversely, in C-rich soils (SOC > 15 g kg^−1^), accumulation mainly occurs through an enhanced microbial C pump and increased mineral protection, leading to a shift towards MAOC. As depicted by Fig. 5, by carrying out four long-term N fertilizer networking experiments, we observed that N fertilization altered soil functional C pools depended on initial SOC content, orchestrating an integrated control that encompasses plant, geochemical, and microbial factors. Our findings contribute to the nuanced understanding of N fertilizer’s role in promoting SOC sequestration, laying the groundwork for revising existing models of soil C dynamics. The dichotomy in SOC accumulation mechanisms we have uncovered – more pronounced POC accumulation in C-poor soils versus MAOC accrual in C-rich conditions - challenges and extends traditional notions of N fertilization impacts. This divergence underscores the complexity of plant-microbe-soil matrix interactions in mediating these effects. Future theoretical frameworks should incorporate the diversity of soil responses to N addition, emphasizing the interplay between biological and geochemical factors.

## Methods

### Field site description

Four coordinated long-running field N fertilizer experiments network were established between 2008 and 2011 in the four main maize production regions in China and represent four dominant soil types (Supplementary Fig. 1). The four sites were located in Siping (SP) Experimental Station, Jilin Province in Northeast China (43°20’N, 124°20′E); Quzhou Experimental Station (QZ), Hebei Province in the North China Plain (36°53’N, 115°12′E); Changwu Experimental Station (CW), Shaanxi Province in Northwest China (35°28’N, 107°88′E); and Yaan Experimental Station (YA), Sichuan Province in Southwest China (29°59’N, 103°14’E). At Siping, the soil texture is silt clay with 16.3 g kg^−1^ SOC, 1.69 g kg^−1^ TN and pH 6.16. The area had an average air temperature of 6.81 °C and mean annual precipitation of 551 mm. In Quzhou experimental station, the soil texture is clay loam with 9.49 g kg^−1^ SOC, 0.87 g kg^−1^ TN and pH 8.5. The area had an average air temperature of 13.2 °C and mean annual precipitation of 516 mm. In Changwu experimental station, the soil texture is silt loam with 9.51 g kg^−1^ SOC, 0.95 g kg^−1^ TN and pH, 8.4. The area had an average air temperature of 9.2 °C and mean annual precipitation of 595 mm. In Yaan experimental station, the soil texture is clay loam with 17.4 g kg^−1^ SOC, 1.29 g kg^−1^ TN and pH, 6.3. The area had an average air temperature of 17.1 °C and mean annual precipitation of 1635 mm. Detailed site information of the four sampling regions is summarized in Supplementary Table 7.

The same fertilizer experiments were established in a randomized complete-block design with four replicates at each site. N fertilizer was added as urea [CO(NH_2_)_2_] at three N levels: (1) no N-fertilizer (hereafter called ‘Control’); (2) optimized N rate (hereafter called ‘OPT’); (3) high N rate (hereafter called ‘HN’). In brief, the optimal N application rate was determined based on the in-season root-zone N management to closely synchronize the soil and environment N supply with demand for N by the crop, while the high N rate is comparable to local farmers’ practices^61^. Fertilizer was applied twice during the growing period: initially as a basal application and subsequently as a top-dressing in all four sites. Maize straw was chopped and incorporated into the soil through tillage to a depth of ~20 cm.

### Soil and plant sampling and analysis

Topsoils (0–20 cm) were sampled in September 2020, and five topsoil cores taken randomly from each plot were composited as on sample per plot. The soil samples were sieved through a 2-mm sieve with all visible roots and rocks were removed except for the aggregate fractionation and were divided into two parts: one was stored at 4 °C for enzymes activities and microbial composition analysis within one week, and the remaining soil was stored at room temperature for determining soil physicochemical analyzes and further incubation experiment.

The SOC and total nitrogen (TN) contents were determined by dry combustion using an elemental analyzer (Elementar, Vario Max CN, Germany). Before measuring the SOC and TN, the alkaline soil was fumigated with concentrated HCl for 48 h to remove carbonates. The soil pH was measured using an ULTRAMETERII™ 6PFCE (Mellon, California, USA) after shaking the soil in deionized water (1:2.5 w/v) suspensions for 30 min. Microbial biomass C (MBC) and N (MBN) was extracted in 0.5 M K_2_SO_4_ solution from chloroform fumigated and unfumigated soil and determined by the difference in C and N concentration using a Shimadzu TOC-L analyzer^62^. The fungal-to-bacterial ratio (F:B) was assessed by applying the phospholipid fatty acids method^63^.

At maturity in 2018, four complete root systems were collected from the topsoil (0–20 cm) in each plot. The roots were separated from the soil by rinsing on a fine mesh screen under a gentle water spray until most of the soil was removed. The cleaned roots were then dried at 40 °C, sieved to retain the roots while dislodging any remaining soil, and subsequently weighed.

### Soil enzymes activities, microbial metabolic efficiency and nutrient limitation

The hydrolytic enzymes, α-glucosidase (BG), cellobiohydrolase (CBH), *N*-acetyl-β-D-glucosaminidase (NAG), leucine amino peptidase (LAP), acid and alkaline phosphatase (ACP and ALP) in fresh soil samples were measured fluorometrically using methylumbelliferone-labeled substrates^64^. Briefly, 125 mL of buffer solution was added to 1.0 g dry mass of fresh soil adjusted pH to 8.2 with HCl. For ALP and ALP analysis, the buffer was adjusted to pH 6.5 and 8.2, respectively. A total of 200 μL sample suspension and 50 μL substrate solutions were dispensed into the wells of a black 96-well microplate. The microplates were covered and incubated in the dark at 25 °C for 4 h and the fluorescence quantified using a microplate fluorometer with 365-nm excitation and 450-nm emission filters. Polyphenol oxidase (PPO) and peroxidase (POD) were measured spectrophotometrically in a clear 96-well microplate using the substrate of L-3,4-dihydroxy-phenylalanine (L-DOPA; German et al. ^64^). A 1 mL aliquot of soil slurry was mixed with 1 mL of 10 mM L-DOPA in 50 mM acetate buffer for PPO or 1 mL L-DOPA and 0.2 mL of hydrogen peroxide for POD and then incubated in the dark at 20 °C for 2 h. The suspensions were centrifuged, and the activity quantified by measuring absorbance at 450 nm.

Enzymatic stoichiometric was employed to assess potential C limitations in the soil. The vector length were calculated according to Moorhead et al. ^65^. Vector length indicates the enzyme activity towards C acquisition relative to N and P. A Higher vector length represents larger microbial C limitation^65^.

1 $${VL}=\sqrt{{x}^{2}+{y}^{2}}$$

where *x* and *y* represent the relative C:P and C:N acquiring enzyme ratios, respectively. C-acquiring enzyme: BG and CBH; N-acquiring enzyme: NAG and LAP; P-acquiring enzyme: ACP for SP and YA, ALP for QZ and CW.

We also assessed microbial resource limitation by calculating the ratios of the resource C:N ratios (R_C:N_), normalized to the ratio of MBC to MBN (B_C:N_)^23,66^. Given that biomass stoichiometry determines the nutrient requirements of microbial decomposers, greater resource limitation indicates increasing microbial N limitation.

2 $${Resource\; limitation}={R}_{C:N}/{B}_{C:N}$$

where *R*_*C:N*_ represents the ratio of SOC-to-TN, *B*_*C:N*_ represents the ratio of MBC-to-MBN.

Microbial metabolic efficiency was assessed using qCO2, with higher qCO2 values indicating less carbon (C) allocation to biomass and greater respiration losses^21^. Soil samples (equivalent to 30 g dry weight) were adjusted to 60% water holding capacity, and placed into 250 ml jars, and incubated at 25 °C for 28 days. Headspace CO_2_ release was measured using a soil CO_2_ flux system equipped with a portable chamber (EGM-5, USA, PP SYSTEM) on days 1, 3, 5, 7, 14, 21, 28 after soil incubation. Soil qCO_2_ was expressed as μg CO_2_-C μg^−1^ MBC h^−1^.

### SOC fractionation

Particulate organic matter (POC) and mineral-associated organic carbon (MAOC) were determined with modifications to the method described by Poeplau et al. ^67^. Briefly, 20 g of 2-mm sieved bulk soil was shaken for 18-h with glass beads in 100 mL 0.5 % sodium hexametaphosphate to disrupt all aggregates. The resulting soil slurry was rinsed with deionized water over a 53-µm sieve with the fraction passing through (< 53-µm) representing MAOC and the fraction remaining on the sieve (> 53-µm) representing POC. Following physical fractionation, total C in MAOC and POC fractions were measured using elemental analysis.

### Compound specific analysis of lignin phenols and amino sugars

Lignin phenols were used as a geochemical proxy for terrestrial plant C input according to Otto & Simpson^68^. Soil samples were ground to pass through a 0.15 mm mesh sieve and a subsample was transferred to a Teflon vessel digestion for digestion using alkaline CuO under high pressure and temperature to release constituent monomer phenols from lignin polyphenols. Briefly, 1 g of air-dried soil (<0.15 mm) was solvent-extracted and base hydrolyzed to remove extractable and hydrolysable lipids. The dried residues were mixed with 1 g CuO, 100 mg ammonium iron(II) sulfate hexahydrate, and 15 ml N_2_-purged 2 M NaOH in teflon-lined bombs. The bombs were flushed with N_2_ for 10 min and heated at 170 °C for 2.5 h. After cooling, lignin oxidation products (LOPs) were spiked with ethyl vanillin, acidified to pH 1 with 6 M HCl, and kept in the dark for 1 h. LOPs were extracted from the supernatant with ethyl acetate, concentrated under N_2_, and dissolved in a solution with trans-cinnamic acid. The mixture was dried under N_2_, derivatized with N,O-bis-(trimethylsilyl) trifluoroacetamide and pyridine (70 °C, 1 h), and quantified using a GC-MS QP 2010 PLUS (Shimadzu) with an HP-5 fused silica column and a flame ionization detector. This method released eight characteristic lignin monomer phenols, including vanillyl (V; vanillin, acetovanillone and vanillic acid), syringyl (S; syringaldehyde, acetosyringone and syringic acid) and cinnamyl (C; p- coumaric acid and ferulic acid) monomers. Vanillyl (V), syringyl (S) and cinnamyl (C) phenols were summed to represent lignin phenols in soils.

Amino sugars analysis was used to quantify microbial necromass contributed by bacteria or fungi according to Zhang & Amelung^69^. Briefly, weighed soil subsamples were hydrolyzed with 6 M HCl for 8 h at 105 °C. Myo-inositol was added as an internal standard after the hydrolysate cooled to room temperature. The hydrolysate was then filtered, evaporated, pH adjusted (6.6–6.8), centrifuged and freeze-dried. Amino sugars were re-dissolved in methanol and separated from salts by centrifugation. After the addition of a quantitative standard (methyl-glucamine), amino sugars were transformed into aldononitrile derivatives. Then reacted with derivative reagent, acetic anhydride, ethyl acetate, and hexane for derivatization. Gas chromatograph (Agilent 6890 A, Agilent Technologies) was utilizing to separated and quantified the three amino sugars. Bacterial and fungal necromass C were calculated using the following equations and microbial necromass C refer to the sum of bacterial and fungal necromass C^63,70^:

3 $${Bacterial\; necromass\; C}={muramic\; acid}\times 45$$

4 $${Fungal\; necromass\; C}=\left({glucosamine}-2\times {muramic\; acid}\right)\times 179.17\times 9$$

where 45 represents the conversion ratio of muramic acid to bacterial necromass C; 179.17 is the glucosamine molecular weight; and 9 indicates the conversion ratio from fungal glucosamine-to-fungal necromass C^71^.

### Soil aggregate size fractionation

Soil aggregates size fractions were determined using wet sieving procedure^72^, in which 100 g soil was separated into four aggregate size class: large macroaggregates (2–8 mm), small macroaggregates (0.25–2 mm), microaggregates (0.053–0.25 mm) and < 0.053 mm fractions. In brief, undisturbed air-dried soil samples were gently crushed to pass an 8-mm sieve mesh. Then, 100 g samples of ~8 mm-sized aggregates were placed in the top sieve of a set containing sieves with 2.0 mm, 0.25 mm, and 0.053 mm mesh sizes that was submerged and oscillated (amplitude ~2.5 cm) in deionized water. The soil material was rinsed from each sieve and then oven-dried and weighed. The mean weight diameter (MWD, mm) indicates aggregate stability, which is calculated as follows:

5 $${MWD}={\sum }_{i=1}^{n}{X}_{i}\times {W}_{i}$$

where *X*_*i*_ is the average diameter of aggregates in any size fraction; *W*_*i*_ is the proportion of aggregates corresponding to *X*_*i*_.

### Solid-state ^13^C nuclear magnetic resonance (NMR) analysis

The chemical composition of SOC was investigated by determining the relative abundance of functional groups using solid-state ^13^C cross-polarization and magic-angle-spinning (CPMAS) NMR^53^. Soil samples were treated with 10% HF-HCL solution to concentrate the organic matter and to remove paramagnetic minerals. The ^13^C-CPMAS NMR spectra were acquired using an AVANCE III 400 WB spectrometer (Bruker, Billerica, MA, USA). The sold-state ^13^C-CPMAS NMR spectra were divided into four common chemical shift regions: alkyl-C (0–45 ppm), O-alkyl-C (45−110 ppm), Aromatic-C (110−160 ppm) and Carbonyl-C (160–220 ppm). Different indices of SOC stability were calculated as follows:

6 $${Aliphaticity}={alkyl\; C}/{O\; alkyl\; C}$$

7 $${Recalcitrance}=\left({alkyl}+{aromatic\; C}\right)/\left({O\; alkyl}+{carboxyl\; C}\right)$$

### Mineral protection

Polyvalent cations provide a mechanism for carbon retention through their role in cation bridging. We measured the content of exchangeable cations including Fe/Al-(hydr) oxides^73^. To measure Fe/Al oxides, pyrophosphate-extractable Fe/Al oxides (Fe_p _+ Al_p_) extracted using 0.2-M sodium pyrophosphate, and oxalate-extractable Fe/Al oxides (Fe_o _+ Al_o_) extracted using 0.2-M oxalic acid-ammonium oxalate (pH = 3), were used to represent organically complexed Fe/Al oxides and poorly crystalline oxyhydroxides, respectively.

Exchangeable calcium (Ca_exe_) and magnesium (Mg_exe_) ions safeguard C in soils were extracted with ammonium chloride-70% ethanol solution after removing the soluble chloride and sulfate by 70% ethanol solution^73^. The contents of Fe/Al, Ca_exe_ and Mg_exe_ mineral phases were determined with an inductively coupled plasma optical emission spectrometer (ICP-OES; iCAP 6300, Thermo Scientific, Waltham, USA).

### Compilation of the global database

To investigate changes of soil C functional pools (SOC, POC and MAOC) response to N fertilizer application under influences of different initial SOC levels in global agricultural land, we compiled experimental research articles published prior to June 2024 from the Web of Science and the China National Knowledge Infrastructure Database using the following keywords: (“nitrogen”) AND (“soil carbon fraction” OR “soil mineral-associated organic carbon” OR “soil mineral-associated organic matter” OR “MAOC” OR “MOC” OR “MAOM” OR “MOM” OR “particulate organic matter” OR “particulate organic carbon” OR “POC” or POM”) AND (“farmland” OR “cropland” OR “agriculture”). For in inclusion in the database, published research must have satisfied the following criteria: (1) a N fertilization experiment was conducted in the field and included N-addition and control treatments within agroecosystems under the same environmental conditions; (2) only studies conducted at upland fields were included; (3) no other organic manure was applied in all treatments during the experiments; (4) C pools and at least one related variable were measured, and the mean, standard deviation or standard error, and sample size were reported for all treatments; To ensure comparability with our four-site experiment, we selected only the data from trials conducted on dryland. Implementing these criteria, 118 published articles were selected (Supplementary References). We compiled a final dataset containing 494 and149, measurements of POC and MAOC, respectively. Data were also collected on site coordinates, climate, initial soil organic carbon, initial soil pH, initial total nitrogen, initial silt and clay content (all initial soil properties refer to those before the start of the experiment.), N input rate, and duration. Crop yield values were also extracted if available. When data were presented graphically only, figures were digitized to extract the numerical values using GETDATA GRAPH. If soil and climatic variables were not reported, we supplemented the missing data using global databases (e.g., soilgrids and WorldClim database). We classified data into tropical, subtropical, temperate and boreal ecozones based on site-specific coordinates and Food and Agriculture Organization ecozones.

### Predictive modeling of global POC and MAOC

To create a spatially predictive model of the effect of current N fertilizer on POC and MAOC, we first sampled our prepared stack of environmental covariates at each independent data point within the compiled database. These layers included climatic, soil, and fertilizer use (Supplementary Table 6). Specifically, values of all grids of environmental layers were extracted based on site-specific coordinates or directly from original studies when available. All covariates were resampled and reprojected to a unified pixel grid in EPSG:4326 (WGS84) at five-arcminute spatial resolution. We selected four common and reliable machine-learning models (random forest model, the extreme gradient-boosting model, the light gradient-boosting machine model, and the gradient-boosting decision trees mode) to predict POC and MAOC changes. The data used for training and validation are 80% and 20%, respectively. We used a grid-search procedure, with five-fold cross-validation, to select the best model. The predictive power of the models was measured using three key metrics: the Root Mean Square Error (RMSE), the Mean Absolute Error (MAE), and the goodness of fit, as represented by the coefficient of determination (R^2^). The results showed that the gradient-boosting decision trees machine model performed best among all models (characterized by the maximum R^2^ and the minimum MAE and MSE; Supplementary Table 5) and was therefore selected to predict the global variations in POC/MAOC. All models were run in Python.

### Statistical analysis

For meta-analysis, we calculated the mean effect size and 95% CI of the overall effects of N fertilizer managements on SOC, POC and MAOC. The effects of N fertilization were estimated based on the natural log-transformed response ratio (*RR*)^74^, confidence intervals (CIs) on the weighted effect size were generated using boot-strapping (999 iterations, function confint, package metafor):

8 $${{\mathrm{ln}}}{RR}={{\mathrm{ln}}}\left(\bar{{X}_{t}}/\bar{{X}_{c}}\right)={\mathrm{ln}}\left({X}_{t}\right)-{\mathrm{ln}}({X}_{c})$$

Where *X*_*t*_ is the treatment mean, and *X*_*c*_ is the control mean. The variance (*v*) associated with each value of lnRR was calculated as follows:

9 $$v=\frac{{S}_{t}^{2}}{{n}_{t}\overline{{X}_{t}^{2}}}+\frac{{S}_{c}^{2}}{{n}_{c}\overline{{X}_{c}^{2}}}$$

Where *n*_*t*_ and *n*_*c*_ are the sample sizes for the treatment and control groups, respectively; and *s*_*t*_ and *s*_*c*_ are the SDs for the treatment and control groups, respectively.

We first fitted linear and non-linear (quadratic and general additive models [GAM]) regressions to the relationships between the effect sizes of POC/MAOC and initial SOC content, and used the Akaike information criterion (AIC) to decide which model was the best fit in each case^75,76^. Thresholds could only be defined when non-linear regressions best fit the data. Then, we evaluated six different nonlinear models to select the one that best aligned with our data (Supplementary Table 1), and which each differed from each other by the functions fitted either side of the estimated breakpoint^77^. The nonlinear models evaluated were: general additive models (GAM), step (two intercept models, for which the differences in intercept were tested at the breakpoint), segmented (two linear models in which the slope is changed at breakpoint), stegmented (two linear models in which both the slope and the intercept are changed at the breakpoint), hinge model 12 (one linear model is fitted for the left part of the breakpoint and a second degree polynomial is fitted for the right part), and hinge model 22 (two different second degree polynomial models are fitted at both sides of the breakpoint). The model exhibiting the lowest AIC and Bayesian information criterion (BIC) was considered the best model. Detailed AIC and BIC values for each regression model are provided in Supplementary Table 1. We defined the breakpoint as the initial SOC threshold.

To further test whether the thresholds identified significantly affected the slope of the fitted regressions, we bootstrapped linear regressions either side of each threshold for each variable. We then extracted the slope and the predicted value of the variable evaluated before and after the threshold and compared them using a Mann-Whitney U test following the methods in refs. ^75,76^. We also used the Q-statistic to test the heterogeneity of the N effect sizes on POC and MAOC between before and after the SOC threshold. Significant Q_M_ values (between-group heterogeneity) indicated that the RR differed between groups (*P* < 0.05).

We assessed the quality of our meta-analysis using the checklist developed by Koricheva and Gurevitch (Supplementary Table 8^78^;). Our meta-analysis met all the quality criteria established for meta-analyzes in ecology. The assessment of publication bias conventionally employs funnel plot analysis. However, numerous studies lack the necessary sample variance information crucial for the construction of informative funnel plots. Consequently, a histogram was generated to examine the distribution of individual effect sizes across the dataset, aiming to ascertain any indications of publication bias (Supplementary Fig. 16). Egger’s test suggests that the impact of nitrogen fertilizer management on POC may be slightly overestimated, yet it appears to have no significant effect on MAOC. The failsafe number for both POC and MAOC exceeds the threshold of 5n + 10 (*N* is the sampling size in this study), suggesting an absence of publication bias. We performed a Jackknife sensitivity analysis to assess the impact of individual studies on the overall robustness of the analysis^79^. In the Jackknife analysis, each study received a unique identification, and, in each iteration, data from one study were systematically excluded from the dataset (Supplementary Fig. 17). The sensitivity analysis demonstrated the robustness of these findings, with no specific publication exerting a disproportionate influence on the overall response. In addition, temporal change tests show significant correlation between POC with publication year (*P* < 0.05; MAOC: *P* > 0.05), we can still infer that our results are robust as the estimates are small-scale (*β* = 0.02 for POC; Supplementary Fig. 18).

For the field, experiments, we used linear mixed-effect models to determine the effects of N fertilizer practices, initial C conditions and their interactions on root biomass, soil C pools, microbial properties and necromass, lignin phenols and mineral and physical protection. Statistical significance is based on Wald type III χ² tests and all estimated effect sizes (*β*) are based on rescaled response variables. Specifically, the preceding responsive variables for each experiment were tested with N fertilizer managements as fixed effects, and site were tested as random effects (function lmer, package *lme4* and *lmertest*). Pairwise treatment comparisons were assessed via Tukey’s honest significant difference (function emmeans, package *emmeans*). The statistical analyzes were performed in R version 4.2.1.

Partial correlation analysis was used to assess the relationships between C fractions (POC and MAOC) with five groups of impact factors (i.e., C origin (root biomass, lignin phenols and microbial necromass C), transform (qCO_2_, Oxidase, F:B, resource limitation and C limitation), stability (MWD, Fe_p _+ Al_p_, Fe_o _+ Al_o_, Ca_exe_ and Mg_exe_) and structure (recalcitrance and aliphaticity). The greater the difference in partial correlation coefficient between zero-order and controlling correlation, the stronger the effect of control factor. After identifying the key predictors, the correlation between C fraction and these factors were analyzed by univariate linear regression.

### Reporting summary

Further information on research design is available in the Nature Portfolio Reporting Summary linked to this article.

## Supplementary information

Supplementary Information

Peer Review file

Reporting Summary

## Source data

Source Data

## Data Availability

The data used in this study are available in Source data and online in the Zenodo database (10.5281/zenodo.10374039). Source data are provided with this paper.

## Code availability

The analysis code that supports the findings of this study is available on GitHub (https://github.com/ling9802/articles_code_backup/tree/main/N_fertilizer_C%20pools).

## References

[1] Sanderman, J., Hengl, T. & Fiske, G. J. Soil carbon debt of 12,000 years of human land use. *Proc. Natl Acad. Sci.* **114**, 9575–9580 (2017). 28827323 10.1073/pnas.1706103114 PMC5594668

[2] Ma, Y. et al. Global crop production increase by soil organic carbon. *Nat. Geosci.* **16**, 1159–1165 (2023).

[3] Oldfield, E. E., Bradford, M. A. & Wood, S. A. Global meta-analysis of the relationship between soil organic matter and crop yields. *SOIL* **5**, 15–32 (2019).

[4] Lal, R. Enhancing crop yields in the developing countries through restoration of the soil organic carbon pool in agricultural lands. *Land Degrad. Dev.* **17**, 197–209 (2006).

[5] Vendig, I. et al. Quantifying direct yield benefits of soil carbon increases from cover cropping. *Nat. Sustain.* **6**, 1125–1134 (2023).

[6] Vitousek, P. M. et al. Nutrient Imbalances in Agricultural Development. *Science* **324**, 1519–1520 (2009). 19541981 10.1126/science.1170261

[7] Beillouin, D. et al. A global meta-analysis of soil organic carbon in the Anthropocene. *Nat. Commun.* **14**, 3700 (2023). 37349294 10.1038/s41467-023-39338-z PMC10287672

[8] Liu, B. et al. Co-benefits for net carbon emissions and rice yields through improved management of organic nitrogen and water. *Nat. Food* **5**, 241–250 (2024). 38486125 10.1038/s43016-024-00940-z

[9] Tang, B., Rocci, K. S., Lehmann, A. & Rillig, M. C. Nitrogen increases soil organic carbon accrual and alters its functionality. *Glob. Change Biol.* **29**, 1971–1983 (2023). 10.1111/gcb.16588 36607159

[10] Cai, A. et al. Manure acts as a better fertilizer for increasing crop yields than synthetic fertilizer does by improving soil fertility. *Soil Tillage Res.* **189**, 168–175 (2019).

[11] Kazanski, C. E., Riggs, C. E., Reich, P. B. & Hobbie, S. E. Long-term nitrogen addition does not increase soil carbon storage or cycling across eight temperate forest and grassland sites on a sandy outwash plain. *Ecosystems* **22**, 1592–1605 (2019).

[12] Rocci, K. S., Lavallee, J. M., Stewart, C. E. & Cotrufo, M. F. Soil organic carbon response to global environmental change depends on its distribution between mineral-associated and particulate organic matter: a meta-analysis. *Sci. Total Environ.* **793**, 148569 (2021). 34328984 10.1016/j.scitotenv.2021.148569

[13] Eisenstein, M. Natural solutions for agricultural productivity. *Nature* **588**, S58–S59 (2020). 33299207 10.1038/d41586-020-03445-4

[14] Neal, A. L. et al. Arable soil nitrogen dynamics reflect organic inputs via the extended composite phenotype. *Nat. Food* **4**, 51–60 (2023). 37118575 10.1038/s43016-022-00671-z

[15] Cambardella, C. A. & Elliott, E. T. Particulate soil organic-matter changes across a grassland cultivation sequence. *Soil Sci. Soc. Am. J.* **56**, 777–783 (1992).

[16] Kleber, M. et al. Mineral–organic associations: formation, properties, and relevance in soil environments. *Adv., Agron.* **130**, 1–140 (2015).

[17] Cotrufo, M. F., Ranalli, M. G., Haddix, M. L., Six, J. & Lugato, E. Soil carbon storage informed by particulate and mineral-associated organic matter. *Nat. Geosci.* **12**, 989–994 (2019).

[18] Ge, T. et al. Tracking the photosynthesized carbon input into soil organic carbon pools in a rice soil fertilized with nitrogen. *Plant Soil* **392**, 17–25 (2015).

[19] Cotrufo, M. F. & Lavallee, J. M. Soil organic matter formation, persistence, and functioning: a synthesis of current understanding to inform its conservation and regeneration. *Adv., Agron.* **172**, 1–66 (2022).

[20] Fohrafellner, J. et al. Cover crops affect pool specific soil organic carbon in cropland – a meta‐analysis. *Eur. J. Soil Sci.* **75**, e13472 (2024).

[21] Clayton, J., Lemanski, K. & Bonkowski, M. Shifts in soil microbial stoichiometry and metabolic quotient provide evidence for a critical tipping point at 1% soil organic carbon in an agricultural post-mining chronosequence. *Biol. Fertil. Soils* **57**, 435–446 (2021).

[22] Allen, A. P. & Gillooly, J. F. Towards an integration of ecological stoichiometry and the metabolic theory of ecology to better understand nutrient cycling. *Ecol. Lett.* **12**, 369–384 (2009). 19379132 10.1111/j.1461-0248.2009.01302.x

[23] Yang, L. et al. Microbial life‐history strategies mediate microbial carbon pump efficacy in response to N management depending on stoichiometry of microbial demand. *Glob. Change Biol.* **30**, e17311 (2024). 10.1111/gcb.17311 38742695

[24] Yuan, X. et al. Linkages of stoichiometric imbalances to soil microbial respiration with increasing nitrogen addition: Evidence from a long-term grassland experiment. *Soil Biol. Biochem.* **138**, 107580 (2019).

[25] Zechmeister-Boltenstern, S. et al. The application of ecological stoichiometry to plant–microbial–soil organic matter transformations. *Ecol. Monogr.* **85**, 133–155 (2015).

[26] Xu, H. et al. Impact of nitrogen addition on plant-soil-enzyme C–N–P stoichiometry and microbial nutrient limitation. *Soil Biol. Biochem.* **170**, 108714 (2022).

[27] Ye, C. et al. Reconciling multiple impacts of nitrogen enrichment on soil carbon: plant, microbial and geochemical controls. *Ecol. Lett.* **21**, 1162–1173 (2018). 29781214 10.1111/ele.13083

[28] Tian, D. & Niu, S. A global analysis of soil acidification caused by nitrogen addition. *Environ. Res. Lett.* **10**, 024019 (2015).

[29] Lu, X. et al. Nitrogen addition stimulates soil aggregation and enhances carbon storage in terrestrial ecosystems of China: A meta‐analysis. *Glob. Change Biol.* **27**, 2780–2792 (2021). 10.1111/gcb.15604 33742519

[30] Wang, H. et al. Alterations in substrate stoichiometry control the responses of soil diazotrophs to nutrient enrichment. *Soil Biol. Biochem.* **179**, 108975 (2023).

[31] Chen, J. et al. A keystone microbial enzyme for nitrogen control of soil carbon storage. *Sci. Adv.* **4**, eaaq1689 (2018). 30140736 10.1126/sciadv.aaq1689 PMC6105232

[32] Bonner, M. T. L. et al. Why does nitrogen addition to forest soils inhibit decomposition? *Soil Biol. Biochem.* **137**, 107570 (2019).

[33] Floudas, D. et al. The Paleozoic origin of enzymatic lignin decomposition reconstructed from 31 fungal genomes. *Science* **336**, 1715–1719 (2012). 22745431 10.1126/science.1221748

[34] Man, M., Deen, B., Dunfield, K. E., Wagner-Riddle, C. & Simpson, M. J. Altered soil organic matter composition and degradation after a decade of nitrogen fertilization in a temperate agroecosystem. *Agric. Ecosyst. Environ.* **310**, 107305 (2021).

[35] Yu, F., Zhang, W., Hou, X., Li, Y. & Tong, J. How nutrient loads influence microbial-derived carbon accumulation in wetlands: A new insight from microbial metabolic investment strategies. *Environ. Res.* **217**, 114981 (2023). 36460070 10.1016/j.envres.2022.114981

[36] Even, R. J. & Francesca Cotrufo, M. The ability of soils to aggregate, more than the state of aggregation, promotes protected soil organic matter formation. *Geoderma* **442**, 116760 (2024).

[37] Lehmann, A., Zheng, W. & Rillig, M. C. Soil biota contributions to soil aggregation. *Nat. Ecol. Evol.* **1**, 1828–1835 (2017). 29038473 10.1038/s41559-017-0344-y PMC5701735

[38] Yudina, A. & Kuzyakov, Y. Dual nature of soil structure: The unity of aggregates and pores. *Geoderma* **434**, 116478 (2023).

[39] Begill, N., Don, A. & Poeplau, C. No detectable upper limit of mineral-associated organic carbon in temperate agricultural soils. *Glob. Change Biol.* **29**, 4662–4669 (2023). 10.1111/gcb.16804 37271832

[40] Matus, F. J. Fine silt and clay content is the main factor defining maximal C and N accumulations in soils: a meta-analysis. *Sci. Rep.* **11**, 6438 (2021). 33742022 10.1038/s41598-021-84821-6 PMC7979709

[41] Matus, F. J. et al. Upper limit of mineral-associated organic carbon in temperate and sub-tropical soils: How far is it? *Geoderma Reg.* **37**, e00811 (2024).

[42] García-Palacios, P. et al. Dominance of particulate organic carbon in top mineral soils in cold regions. *Nat. Geosci.* **17**, 145–150 (2024).

[43] Wu, J. et al. Particulate organic carbon is more sensitive to nitrogen addition than mineral-associated organic carbon: A meta-analysis. *Soil Tillage Res.* **232**, 105770 (2023).

[44] Villarino, S. H., Pinto, P., Jackson, R. B. & Piñeiro, G. Plant rhizodeposition: a key factor for soil organic matter formation in stable fractions. *Sci. Adv.* **7**, eabd3176 (2021). 33853771 10.1126/sciadv.abd3176 PMC8046368

[45] Liang, G., Stark, J. & Waring, B. G. Mineral reactivity determines root effects on soil organic carbon. *Nat. Commun.* **14**, 4962 (2023). 37587139 10.1038/s41467-023-40768-y PMC10432558

[46] Cui, Y. et al. Stoichiometric models of microbial metabolic limitation in soil systems. *Glob. Ecol. Biogeogr.* **30**, 2297–2311 (2021).

[47] Zhang, T., Chen, H. Y. H. & Ruan, H. Global negative effects of nitrogen deposition on soil microbes. *ISME J.* **12**, 1817–1825 (2018). 29588494 10.1038/s41396-018-0096-y PMC6018792

[48] Fierer, N. et al. Comparative metagenomic, phylogenetic and physiological analyses of soil microbial communities across nitrogen gradients. *ISME J.* **6**, 1007–1017 (2012). 22134642 10.1038/ismej.2011.159 PMC3329107

[49] Rui, Y. et al. Persistent soil carbon enhanced in Mollisols by well-managed grasslands but not annual grain or dairy forage cropping systems. *Proc. Natl Acad. Sci.* **119**, e2118931119 (2022). 35145033 10.1073/pnas.2118931119 PMC8851490

[50] Niu, B. et al. pH: A core node of interaction networks among soil organo-mineral fractions. *Environ. Int.* **178**, 108058 (2023). 37392731 10.1016/j.envint.2023.108058

[51] Mao, H. et al. Dual role of silt and clay in the formation and accrual of stabilized soil organic carbon. *Soil Biol. Biochem.* **192**, 109390 (2024).

[52] Cotrufo, M. F., Wallenstein, M. D., Boot, C. M., Denef, K. & Paul, E. The Microbial Efficiency-Matrix Stabilization (MEMS) framework integrates plant litter decomposition with soil organic matter stabilization: do labile plant inputs form stable soil organic matter? *Glob. Change Biol.* **19**, 988–995 (2013). 10.1111/gcb.12113 23504877

[53] Lu, X. et al. Nitrogen deposition accelerates soil carbon sequestration in tropical forests. *Proc. Natl Acad. Sci.* **118**, e2020790118 (2021). 33846252 10.1073/pnas.2020790118 PMC8072245

[54] Lessmann, M., Ros, G. H., Young, M. D. & de Vries, W. Global variation in soil carbon sequestration potential through improved cropland management. *Glob. Change Biol.* **28**, 1162–1177 (2022). 10.1111/gcb.15954 PMC9299007 34726814

[55] Xu, Y. et al. Nitrogen addition-driven soil organic carbon stability depends on the fractions of particulate and mineral-associated organic carbon. *Nutr. Cycl. Agroecosyst.* **128**, 269–281 (2024).

[56] Houlton, B. Z. et al. A world of cobenefits: solving the global nitrogen challenge. *Earth’s. Future* **7**, 865–872 (2019). 10.1029/2019EF001222 PMC6733275 31501769

[57] Guenet, B. et al. Can N_2_O emissions offset the benefits from soil organic carbon storage? *Glob. Change Biol.* **27**, 237–256 (2021). 10.1111/gcb.15342 32894815

[58] Gao, Y. & Cabrera Serrenho, A. Greenhouse gas emissions from nitrogen fertilizers could be reduced by up to one-fifth of current levels by 2050 with combined interventions. *Nat. Food*10.1038/s43016-023-00698-w (2023). 37117855 10.1038/s43016-023-00698-w

[59] Liu, F. et al. Divergent changes in particulate and mineral-associated organic carbon upon permafrost thaw. *Nat. Commun.* **13**, 5073 (2022). 36038568 10.1038/s41467-022-32681-7 PMC9424277

[60] Lugato, E., Lavallee, J. M., Haddix, M. L., Panagos, P. & Cotrufo, M. F. Different climate sensitivity of particulate and mineral-associated soil organic matter. *Nat. Geosci.* **14**, 295–300 (2021).

[61] Chen, X. et al. Integrated soil–crop system management for food security. *Proc. Natl Acad. Sci.* **108**, 6399–6404 (2011). 21444818 10.1073/pnas.1101419108 PMC3080987

[62] Vance, E. D., Brookes, P. C. & Jenkinson, D. S. An extraction method for measuring soil microbial biomass C. *Soil Biol. Biochem.* **19**, 703–707 (1987).

[63] Tian, J. et al. Microbially mediated mechanisms underlie soil carbon accrual by conservation agriculture under decade-long warming. *Nat. Commun.* **15**, 377 (2024). 38191568 10.1038/s41467-023-44647-4 PMC10774409

[64] German, D. P. et al. Optimization of hydrolytic and oxidative enzyme methods for ecosystem studies. *Soil Biol. Biochem.* **43**, 1387–1397 (2011).

[65] Moorhead, D. L., Sinsabaugh, R. L., Hill, B. H. & Weintraub, M. N. Vector analysis of ecoenzyme activities reveal constraints on coupled C, N and P dynamics. *Soil Biol. Biochem.* **93**, 1–7 (2016).

[66] Chen, L. et al. Regulation of priming effect by soil organic matter stability over a broad geographic scale. *Nat. Commun.* **10**, 5112 (2019). 31704929 10.1038/s41467-019-13119-z PMC6841703

[67] Poeplau, C. et al. Isolating organic carbon fractions with varying turnover rates in temperate agricultural soils – A comprehensive method comparison. *Soil Biol. Biochem.* **125**, 10–26 (2018).

[68] Otto, A. & Simpson, M. J. Evaluation of CuO oxidation parameters for determining the source and stage of lignin degradation in soil. *Biogeochemistry* **80**, 121–142 (2006).

[69] Zhang, X. & Amelung, W. Gas chromatographic determination of muramic acid, glucosamine, mannosamine, and galactosamine in soils. *Soil Biol. Biochem.* **28**, 1201–1206 (1996).

[70] Liang, C., Amelung, W., Lehmann, J. & Kästner, M. Quantitative assessment of microbial necromass contribution to soil organic matter. *Glob. Change Biol.* **25**, 3578–3590 (2019). 10.1111/gcb.14781 31365780

[71] Joergensen, R. G. Amino sugars as specific indices for fungal and bacterial residues in soil. *Biol. Fertil. Soils* **54**, 559–568 (2018).

[72] Six, J., Elliott, E. T., Paustian, K. & Doran, J. W. Aggregation and soil organic matter accumulation in cultivated and native grassland soils. *Soil Sci. Soc. Am. J.* **62**, 1367–1377 (1998).

[73] Chen, L. et al. Soil carbon persistence governed by plant input and mineral protection at regional and global scales. *Ecol. Lett.* **24**, 1018–1028 (2021). 33709557 10.1111/ele.13723

[74] Zhou, R. et al. Microbial necromass in cropland soils: A global meta-analysis of management effects. *Glob. Change Biol.* **29**, 1998–2014 (2023). 10.1111/gcb.16613 36751727

[75] Berdugo, M. et al. Global ecosystem thresholds driven by aridity. *Science* **367**, 787–790 (2020). 32054762 10.1126/science.aay5958

[76] Gross, N. et al. Unforeseen plant phenotypic diversity in a dry and grazed world. *Nature* **632**, 808–814 (2024). 39112697 10.1038/s41586-024-07731-3

[77] Fong, Y., Huang, Y., Gilbert, P. B. & Permar, S. R. chngpt: Threshold regression model estimation and inference. *BMC Bioinf.* **18**, 1–7 (2017). 10.1186/s12859-017-1863-x PMC5644082 29037149

[78] Koricheva, J. & Gurevitch, J. Uses and misuses of meta‐analysis in plant ecology. *J. Ecol.* **102**, 828–844 (2014).

[79] Prairie, A. M., King, A. E. & Cotrufo, M. F. Restoring particulate and mineral-associated organic carbon through regenerative agriculture. *Proc. Natl Acad. Sci.* **120**, e2217481120 (2023). 37186829 10.1073/pnas.2217481120 PMC10214150

