# Author Correction: Soil organic carbon thresholds control fertilizer effects on carbon accrual in croplands worldwide

**DOI:** 10.1038/s41467-025-60069-w

**Published:** 2025-05-23

**Authors:** Jun Ling, Jennifer A. J. Dungait, Manuel Delgado-Baquerizo, Zhenling Cui, Ranran Zhou, Wushuai Zhang, Qiang Gao, Yuanxue Chen, Shanchao Yue, Yakov Kuzyakov, Fusuo Zhang, Xinping Chen, Jing Tian

**Affiliations:** 1 State Key Laboratory of Nutrient Use and Management, College of Resources and Environmental Sciences, China Agricultural University, 100193 Beijing, PR China; 2 Geography, Faculty of Environment, Science and Economy, University of Exeter, Rennes Drive, Exeter, EX4 4RJ UK; 3 Carbon Management Centre, SRUC-Scotland’s Rural College, Edinburgh, EH9 3JG UK; 4 Laboratorio de Biodiversidad y Funcionamiento Ecosistémico, Instituto de Recursos Naturales y Agrobiología de Sevilla (IRNAS), CSIC, 41012 Sevilla, Spain; 5 College of Resources and Environment, Academy of Agricultural Science, Interdisciplinary Research Center for Agriculture Green Development in Yangtze River Basin, Southwest University, 400715 Chongqing, China; 6 College of Resources and Environment, Jilin Agricultural University, 130118 Changchun, China; 7 College of Resources and Environment, Sichuan Agricultural University, 611134 Chengdu, China; 8 Institute of Eco-Environment and Industrial Technology, Shanxi Agricultural University, 030031 Taiyuan, China; 9 Department of Soil Science of Temperate Ecosystems, University of Göttingen, 37077 Göttingen, Germany; 10 Bioeconomy Research Institute, Vytautas Magnus University, Agriculture Academy, Kaunas Reg., Lithuania

**Keywords:** Carbon cycle, Agroecology

Correction to: *Nature Communications* 10.1038/s41467-025-57981-6, published online 27th March 2025

In the Acknowledgements section of this article, the grant numbers relating to the ‘National Natural Science Foundation of China’ given for J.T. was incorrectly given as ‘U32071629’ and should have been **U23A20158 and 32071629**. The original article has been corrected.

