# Supplementary material for: Soil organic carbon thresholds control fertilizer effects on carbon accrual in croplands worldwide
Source: Nat Commun. 2025 Mar 27;16:3009. doi: 10.1038/s41467-025-57981-6 (PMC11950326; doi:10.1038/s41467-025-57981-6)
Supplement: Supplementary file 1 — Supplementary Information [file 41467_2025_57981_MOESM1_ESM.pdf]

Supplementary Information

Title: Soil organic carbon thresholds control fertilizer effects on carbon accrual in croplands worldwide

Authors:

Jun Ling[1], Jennifer A. J. Dungait[2,3], Manuel Delgado-Baquerizo[4], Zhenling Cui[1], Ranran Zhou[1], Wushuai Zhang[5], Qiang Gao[6], Yuanxue Chen[7], Shanchao Yue[8], Yakov Kuzyakov[9,10], Fusuo Zhang[1], Xinping Chen[1,*], Jing Tian[1,*]

Institutional Affiliations:

1 State Key Laboratory of Nutrient Use and Management, College of Resources and Environmental Sciences, China Agricultural University, 100193 Beijing, PR China

2 Geography, Faculty of Environment, Science and Economy, University of Exeter, Rennes Drive, Exeter, EX4 4RJ, UK

3 Carbon Management Centre, SRUC-Scotland's Rural College, Edinburgh, EH9 3JG; UK

4 Laboratorio de Biodiversidad y Funcionamiento Ecosistémico, Instituto de Recursos Naturales y Agrobiología de Sevilla (IRNAS), CSIC, 41012, Sevilla, Spain

5 College of Resources and Environment, Academy of Agricultural Science, Interdisciplinary Research Center for Agriculture Green Development in Yangtze River Basin, Southwest University, 400715 Chongqing, China

6 College of Resources and Environment, Jilin Agricultural University, 130118 Changchun, China

7 College of Resources and Environment, Sichuan Agricultural University, 611134 Chengdu, China

8 Institute of Eco-Environment and Industrial Technology, Shanxi Agricultural University, 030031 Taiyuan, China

9 Department of Soil Science of Temperate Ecosystems, University of Göttingen, 37077, Göttingen, Germany

10 Bioeconomy Research Institute, Vytautas Magnus University, Agriculture Academy,

Kaunas Reg., Lithuania
* Corresponding author
Email: tianj@igsnrr.ac.cn; chenxp2017@swu.edu.cn
Telephone: +86-010-62734554

This PDF file includes:

Supplementary Figure 1 to 18

Supplementary Table 1 to 8

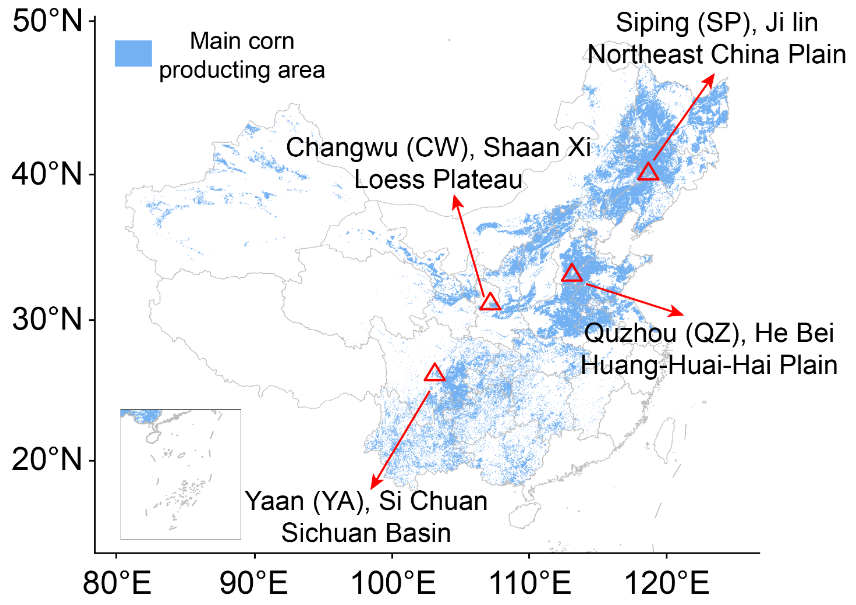

Supplementary Figure 1. Detailed information and enlarged distribution of four nitrogen (N) fertilizer network field experiments across China.

Base map data adapted from GS(2020)4619, http://bzdt.ch.mnr.gov.cn/. The maize distribution in China from https://doi.org/10.57760/sciencedb.08490 [1]. Map created using QGIS software.

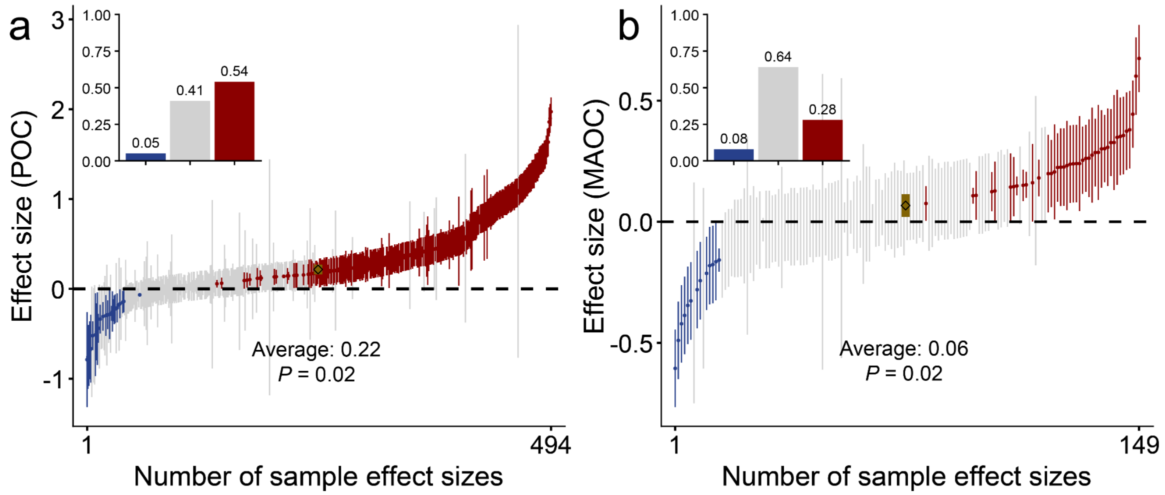

Supplementary Figure 2. Sample effect sizes of POC (a) and MAOC (b) in responding to N addition.

The heights of bar plots are relative proportions of negative (blue), neutral (grey) and positive (red) (CIs crossing zero) effect sizes, and were standardized, ranging from 0 to 1. POC: particulate organic carbon; MAOC: mineral-associated organic carbon. Circles and error bars represent average parameter estimates and 95% confidence intervals (CIs) in the linear mixed effects models (two–sided). The vertical dashed line represents the warming effect size = 0. If the 95% CI did not overlap zero, the effect of warming was statistically significant ($P < 0.05$).

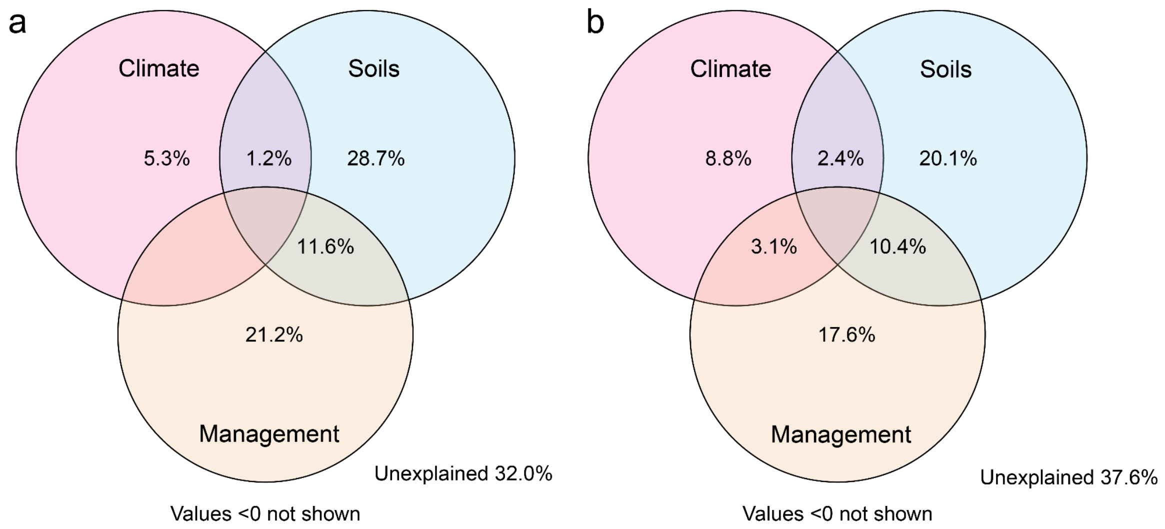

Supplementary Figure 3. Variation partitioning analysis (VPA) of the POC (a) and MAOC (b) contents explained by climatic, soils variables and management practices and their interactions.

Climatic factors: mean annual temperature and mean annual precipitation; Soils variables: initial soil organic carbon, initial soil total nitrogen, initial soil pH and initial silt and clay. Management: nitrogen fertilizer uses and experimental duration. Unexplained represents the variance of changes that are not captured by the Variance Partitioning Analysis.

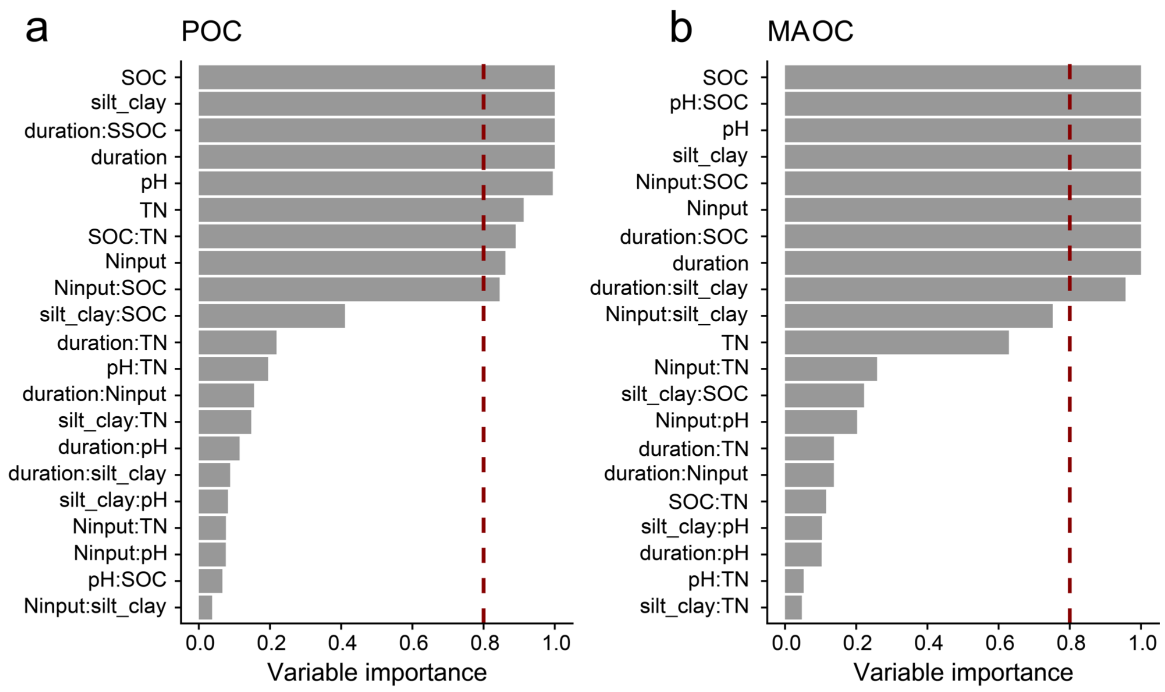

Supplementary Figure 4. Model-averaged importance of the predictors of the effects N addition on POC (a) and MAOC (b) contents.

The importance value is based on the sum of Akaike weights derived from model selection using corrected Akaike's information criteria. Cutoff is set at 0.8 to differentiate between essential and nonessential predictors. POC: particulate organic carbon; MAOC: mineral-associated organic carbon. SOC: soil organic carbon; TN: total nitrogen; N_{input}: N fertilizer use. All soil properties were the initial soil conditions before experiment. The ':' denotes the interaction between factors.

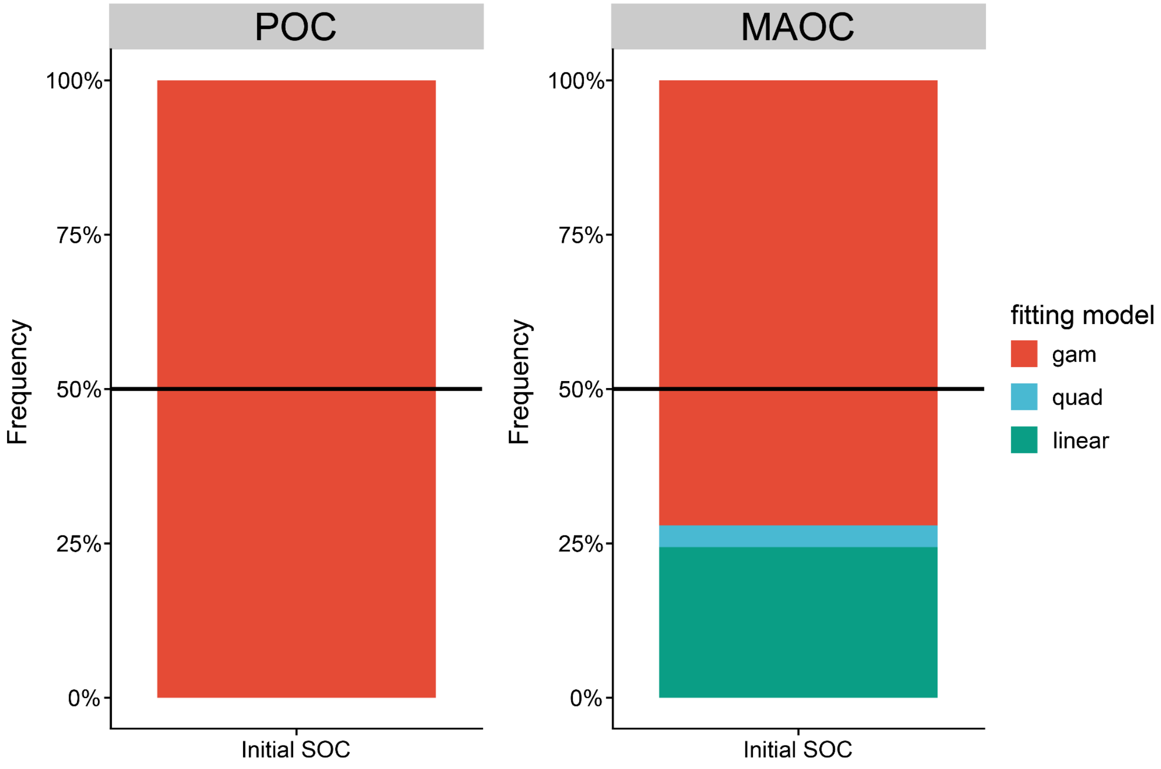

Supplementary Figure 5. Evaluation of linear and nonlinear responses of POC and MAOC contents to initial SOC based on Meta-analysis data.

Akaike information criterion (AIC) was used to decide the model that provided the best fit in each case, and the most likely model has the lowest AIC value. We chose quadratic to synthesize the simplest case of nonlinear trend, and GAM to summarize more complex trends. This comparison was conducted 1000 times by bootstrapping samples and a linear regression was a better fit over the nonlinear relationship only when the linear part ranked above half frequency spotted by horizontal black lines in each facet. SOC: soil organic carbon; POC: particulate organic carbon; MAOC: mineral-associated organic carbon.

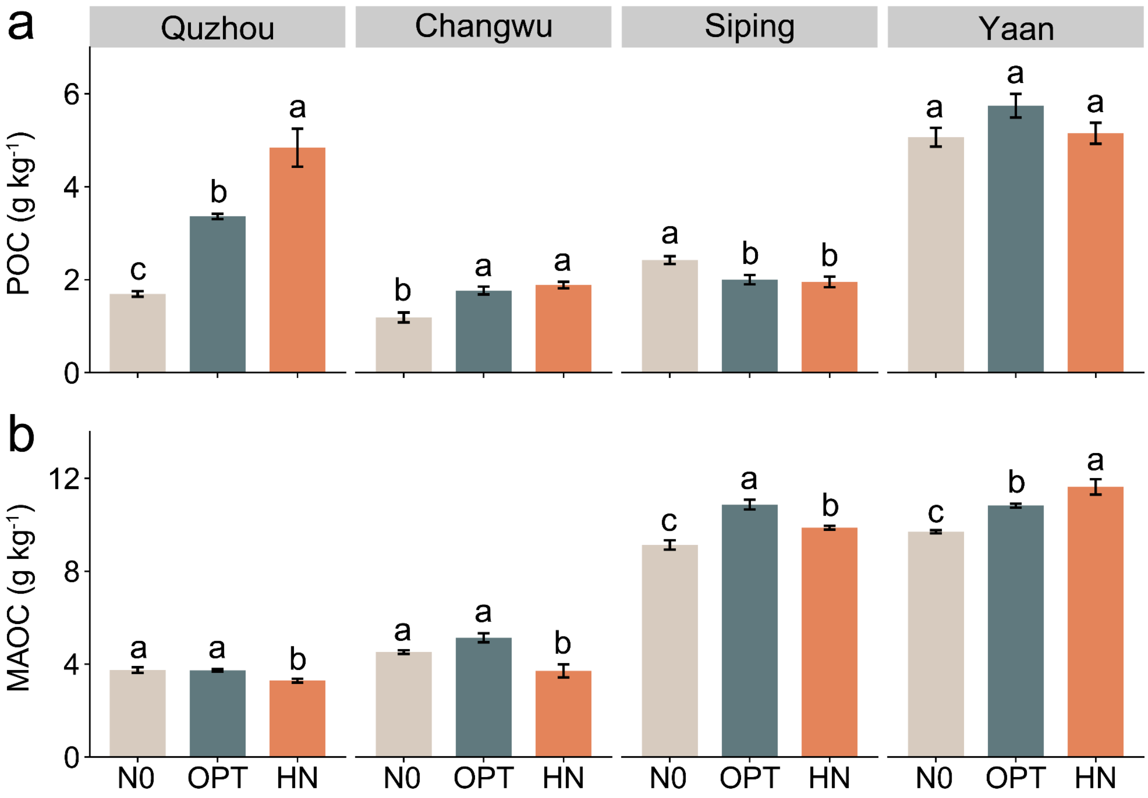

Supplementary Figure 6. Effects of N fertilizer on POC (a) and MAOC contents (b) across four sites.

Data show mean ± standard error (n = 4). Different lowercase letters indicate significant difference among treatments ($P < 0.05$) based on two-sided tests for multiple comparisons (Tukey-post-hoc test). N0: no N fertilizer; OPT: optimized N fertilizer; HN: high nitrogen fertilizer. POC: particulate organic carbon; MAOC: mineral-associated organic carbon.

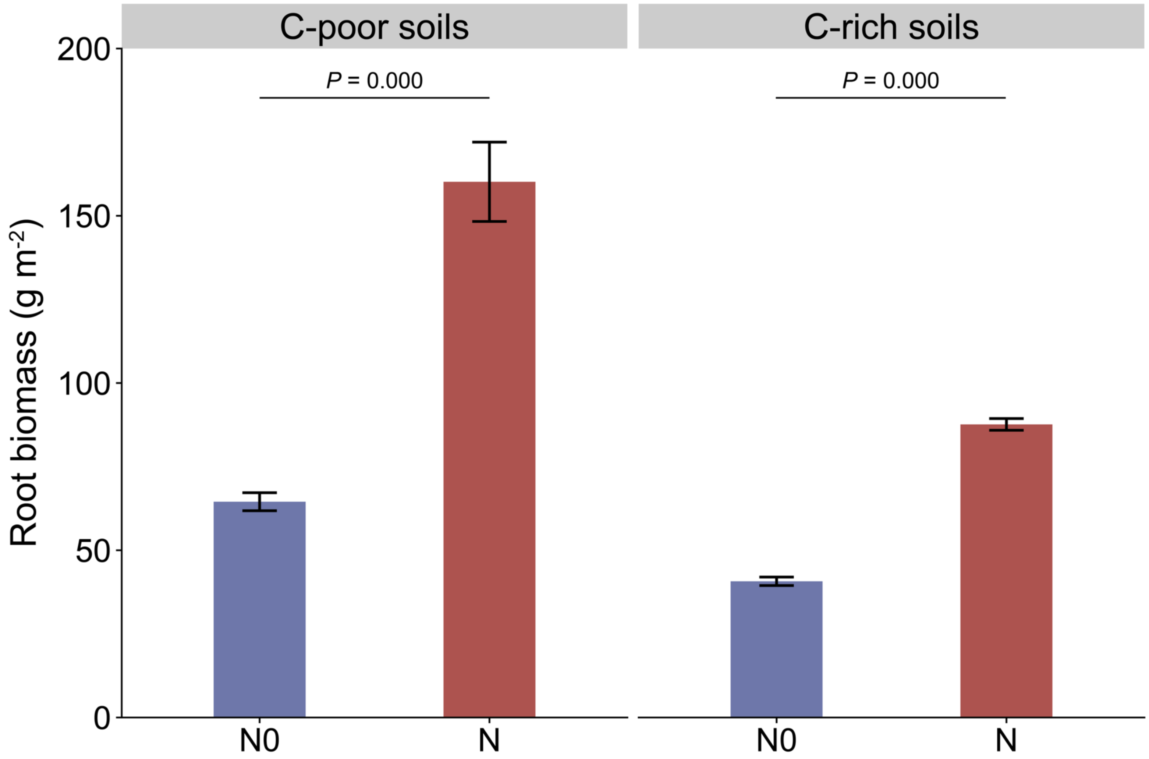

Supplementary Figure 7. Changes of root biomass under different N fertilizer treatments between C-poor and C-rich soils.

Data show mean ± standard error (n = 4 for N0, n = 8 for N). A mixed linear model was used to test the treatment effect. N0: no N addition; N: N addition. C-poor soils: combining QZ and CW results; C-rich soils: combining SP and YA results. C levels are categorized according to the organic carbon threshold (i.e., SOC = 15 g kg^{-1}) determined by the global meta-analysis. A mixed linear model was used to test the treatment effect at P = 0.05.

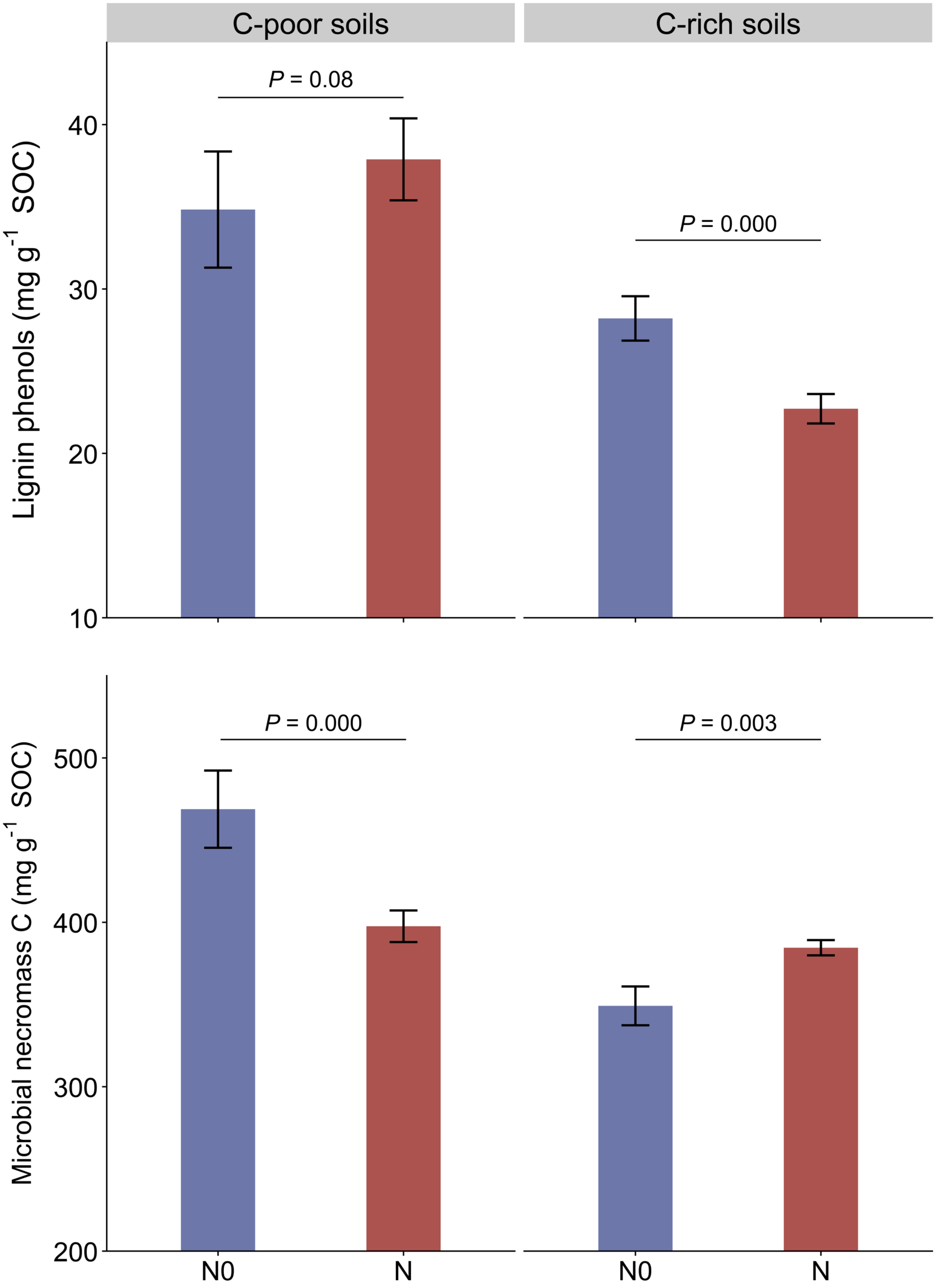

C-poor soils
C-rich soils
Lignin phenols (mg g^{-1} SOC)
Microbial necromass C (mg g^{-1} SOC)
P = 0.08
P = 0.000
P = 0.000
P = 0.003
10
20
30
40
200
300
400
500
N0
N
N0
N

Supplementary Figure 8. Changes of microbial necromass C and lignin phenols under different N fertilizer treatments between C-poor and C-rich soils.

Data show mean ± standard error (n = 4 for N0, n = 8 for N). A mixed linear model was used to test the treatment effect. N0: no N addition; N: N addition. C-poor soils: combining QZ and CW results; C-rich soils: combining SP and YA results. C levels are categorized according to the organic carbon threshold (i.e., SOC = 15 g kg^{-1}) determined by the global meta-analysis. A mixed linear model was used to test the treatment effect at $P = 0.05$.

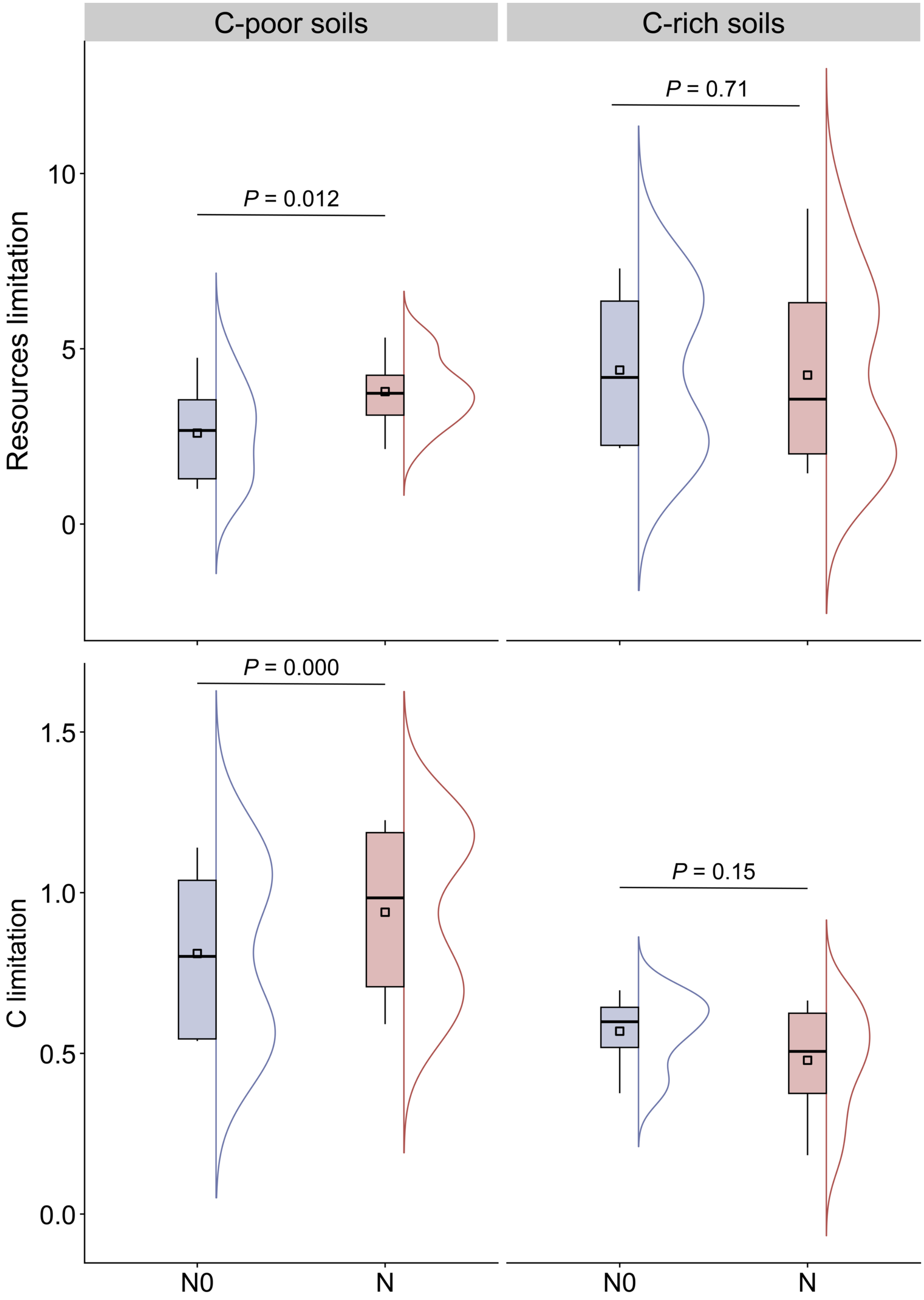
C-poor soils
C-rich soils
Resources limitation
C limitation
P = 0.012
P = 0.71
P = 0.000
P = 0.15
10
5
0
1.5
1.0
0.5
0.0
N0
N
N0
N

Supplementary Figure 9. Microbial resources and C limitation response to N addition between C-poor and C-rich soils.

The resources limitation is the ratio of the resource C:N (SOC:TN) ratios normalized to BC:N (MBC:MBN). The C limitation is the square root of the sum of x^2 and y^2, where x represents the relative activity of C vs. P-acquiring enzymes, and y represents the relative activity of C vs. N-acquiring enzymes. In box plots, the solid lines and squares in the boxes, the lower and upper edges of the boxes and the lower and upper lines extending from the boxes represent median and mean values (n = 4 for N0, n = 8 for N), Q1 and Q3, 1.5 × IQR above the box's Q1 and 1.5 × IQR below the box's Q3 of all data, respectively. A mixed linear model was used to test the treatment effect at $P = 0.05$. SOC: soil organic carbon; TN: total nitrogen; MBC: microbial biomass C; MBN: microbial biomass N; C-acquiring enzymes: the sum of BG (β-1,4-glucosidase) and CBH (cellobiohydrolase); N-acquiring enzymes: the sum of NAG (β-1,4-N-acetylglucosaminidase) and LAP (L-leucine aminopeptidase); N-acquiring enzymes: ALP (alkaline phosphomonoesterase). Asterisks indicate significant differences between N0 and N. N0: no N addition; N: N addition. C-poor soils: combining QZ and CW results; C-rich soils: combining SP and YA results. C levels are categorized according to the organic carbon threshold (i.e., SOC = 15 g kg^{-1}) determined by the global meta-analysis.

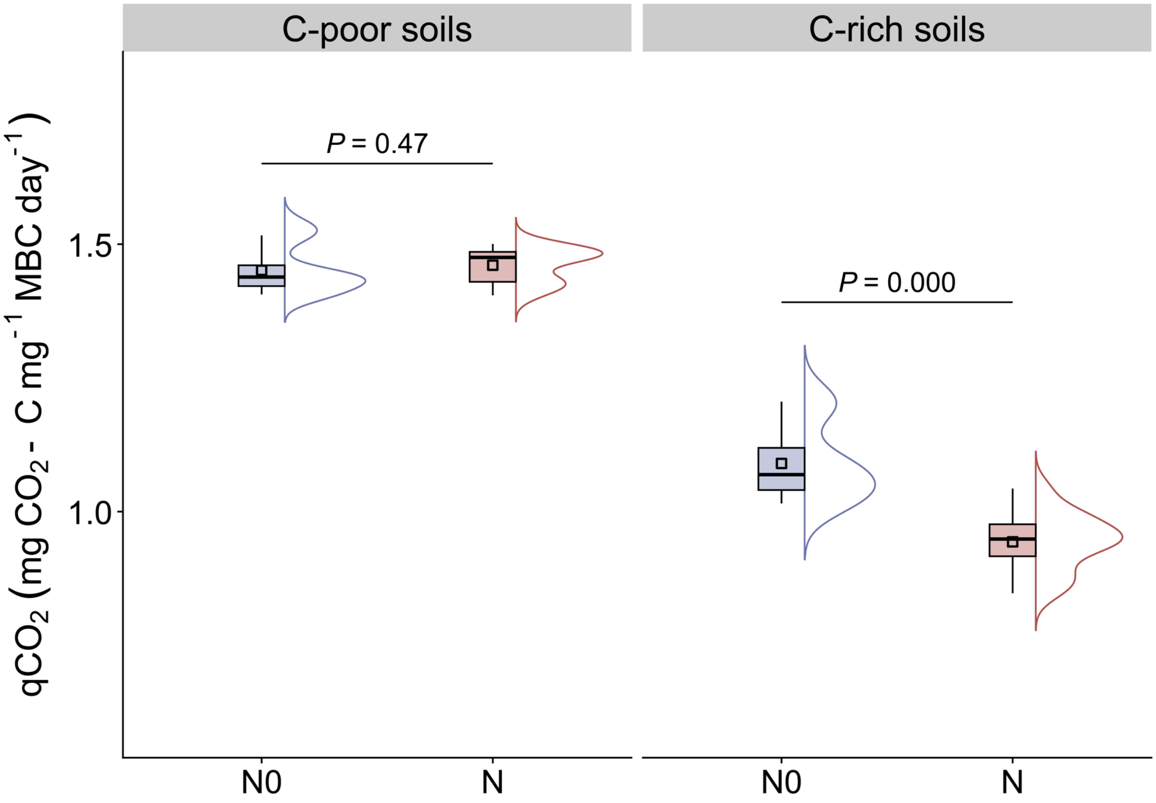

Supplementary Figure 10. Changes of qCO_2 (microbial metabolic quotient) under different N fertilizer treatments between C-poor and C-rich soils.

In box plots, the solid lines and squares in the boxes, the lower and upper edges of the boxes and the lower and upper lines extending from the boxes represent median and mean values (n = 4 for N0, n = 8 for N), Q1 and Q3, 1.5 × IQR above the box's Q1 and 1.5 × IQR below the box's Q3 of all data, respectively. A mixed linear model was used to test the treatment effect at P = 0.05. N0: no N addition; N: N addition. C-poor soils: combining QZ and CW results; C-rich soils: combining SP and YA results. C levels are categorized according to the organic carbon threshold (i.e., SOC = 15 g kg^{-1}) determined by the global meta-analysis.

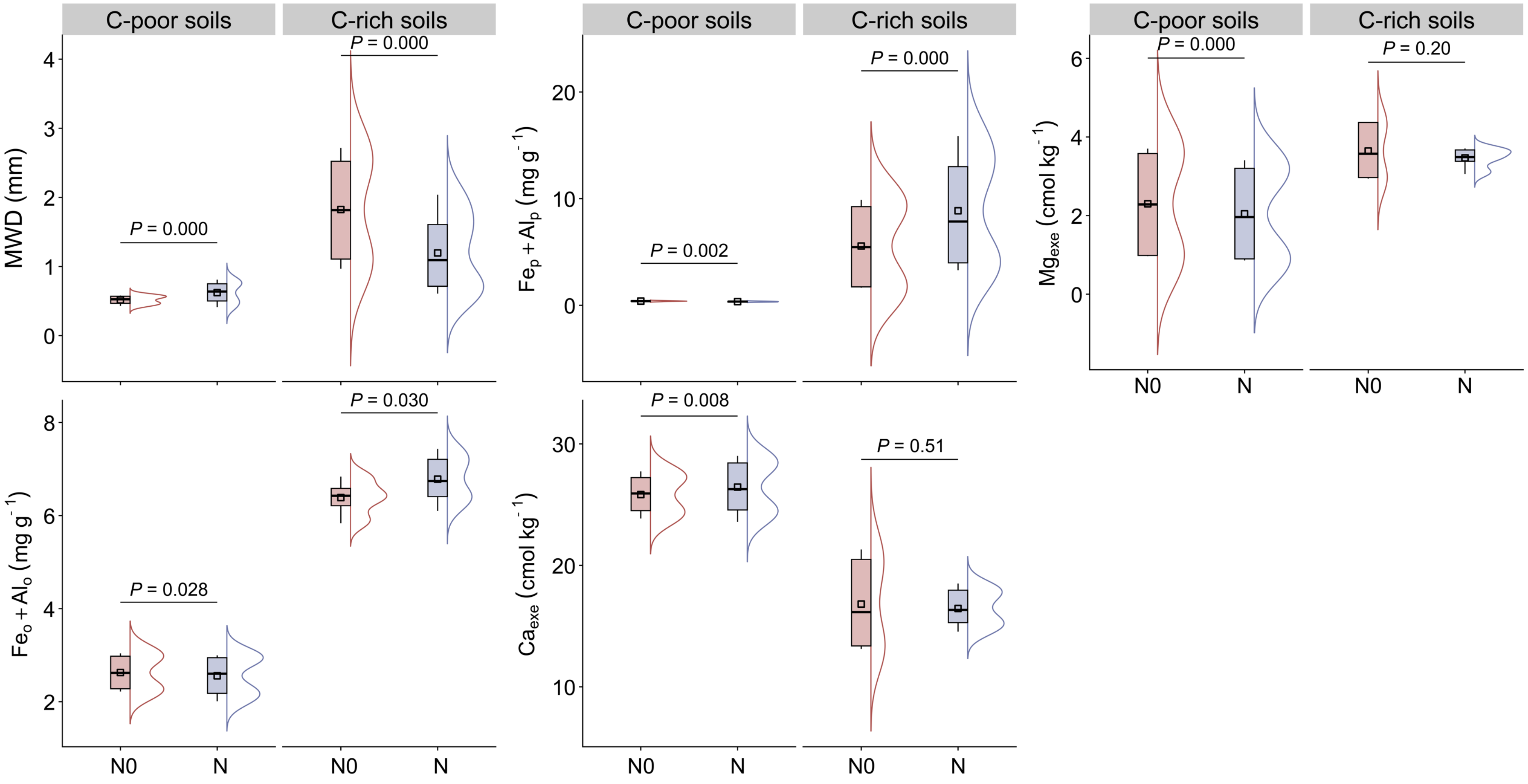
C-poor soils
C-rich soils
MWD (mm)
P = 0.000
P = 0.000
Fe_p + Al_p (mg g^-1)
P = 0.002
P = 0.000
Mg_exe (cmol kg^-1)
P = 0.000
P = 0.20
Fe_o + Al_o (mg g^-1)
P = 0.028
P = 0.030
Ca_exe (cmol kg^-1)
P = 0.008
P = 0.51
N0
N

Supplementary Figure 11. Changes of mean weight diameter (MWD) and mineral protection (Fe_o + Al_o; Fe_p + Al_p) under different N fertilizer treatments between C-poor and C-rich soils.

In box plots, the solid lines and squares in the boxes, the lower and upper edges of the boxes and the lower and upper lines extending from the boxes represent median and mean values (n = 4 for N0, n = 8 for N), Q1 and Q3, 1.5 × IQR above the box's Q1 and 1.5 × IQR below the box's Q3 of all data, respectively. A mixed linear model was used to test the treatment effect at P = 0.05. MWD: mean weight diameter; Fe_o + Al_o: poorly crystalline Fe/Al oxides; Fe_p + Al_p: organically complexed Fe/Al oxides. Asterisks indicate significant differences between N0 and N. N0: no N addition; N: N addition. C-poor soils: combining QZ and CW results; C-rich soils: combining SP and YA results. C levels are categorized according to the organic carbon threshold (i.e., SOC = 15 g kg^{-1}) determined by the global meta-analysis.

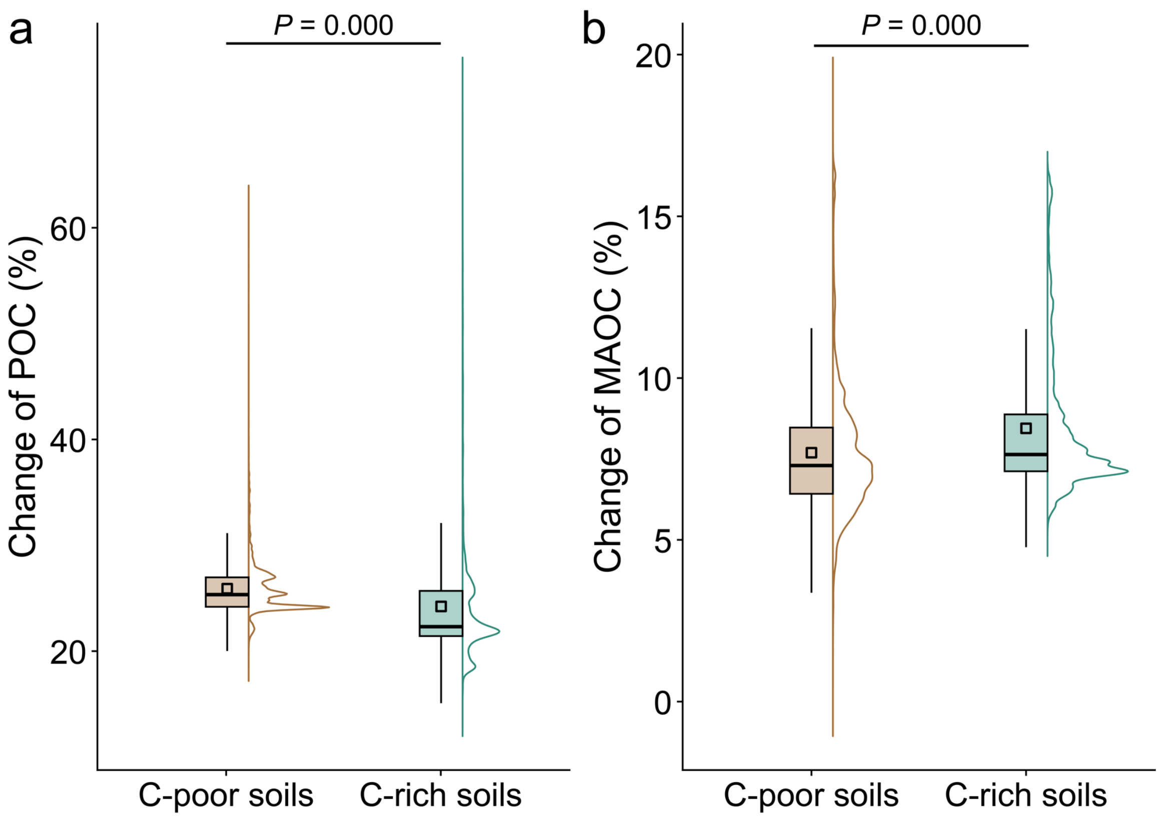

Supplementary Figure 12. Changes in POC (a) and MAOC (b) due to N fertilization in C-poor and C-rich soils at the global scale over the past decade.

In box plots, the solid lines and squares in the boxes, the lower and upper edges of the boxes and the lower and upper lines extending from the boxes represent median and mean values (n = 4 for N0, n = 8 for N), Q1 and Q3, 1.5 × IQR above the box's Q1 and 1.5 × IQR below the box's Q3 of all data, respectively. A mixed linear model was used to test the treatment effect at P = 0.05.C-poor soils: combining QZ and CW results; C-rich soils: combining SP and YA results. C levels are categorized according to the organic carbon threshold (i.e., SOC = 15 g kg^{-1}) determined by the global meta-analysis.

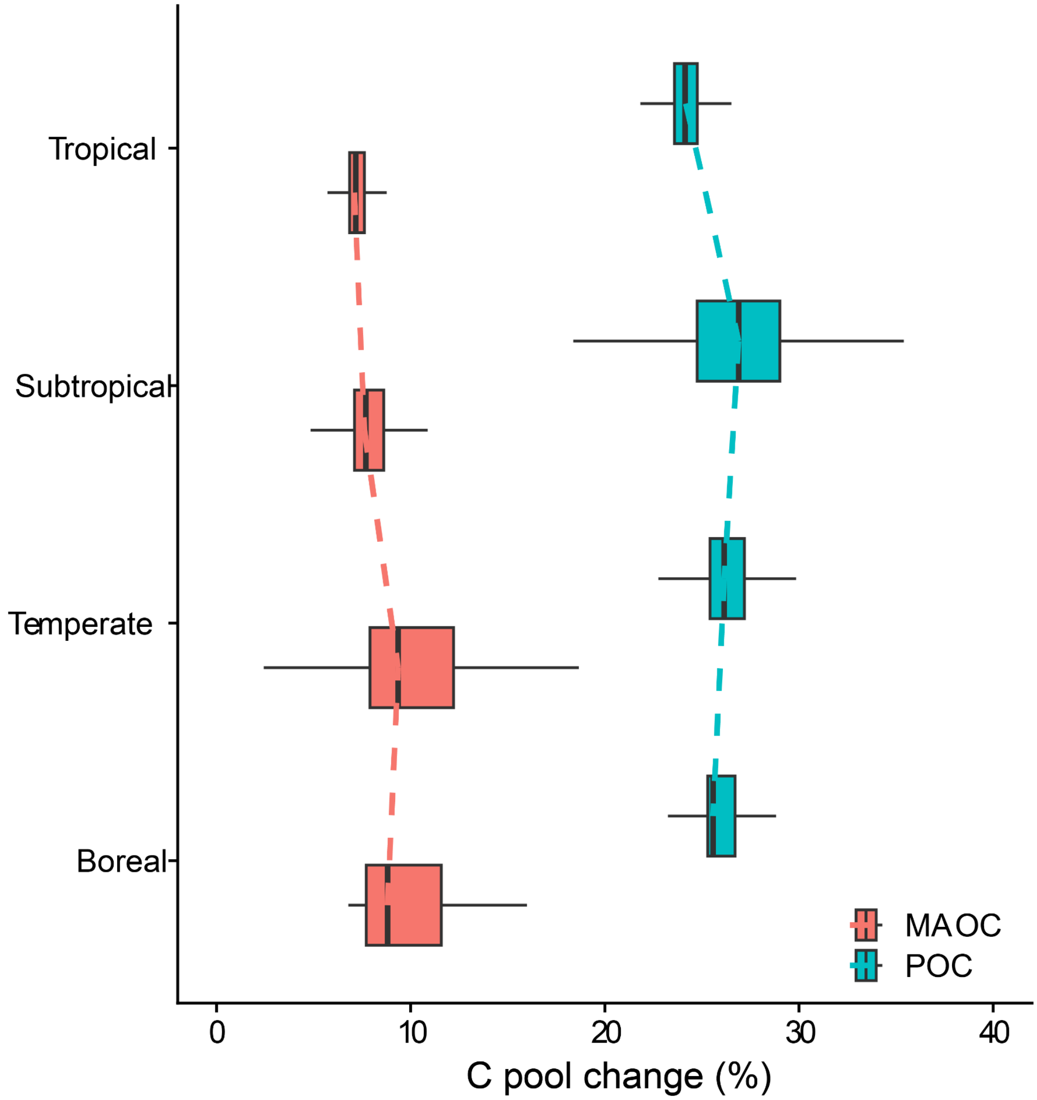

Supplementary Figure 13. POC and MAOC changes due to N fertilization by ecozones over the past decade.

Box plot shows the 25th and 75th percentiles (box borders), median (central black lines) and data ranges (whiskers). POC: particulate organic carbon; MAOC: mineral-associated organic carbon.

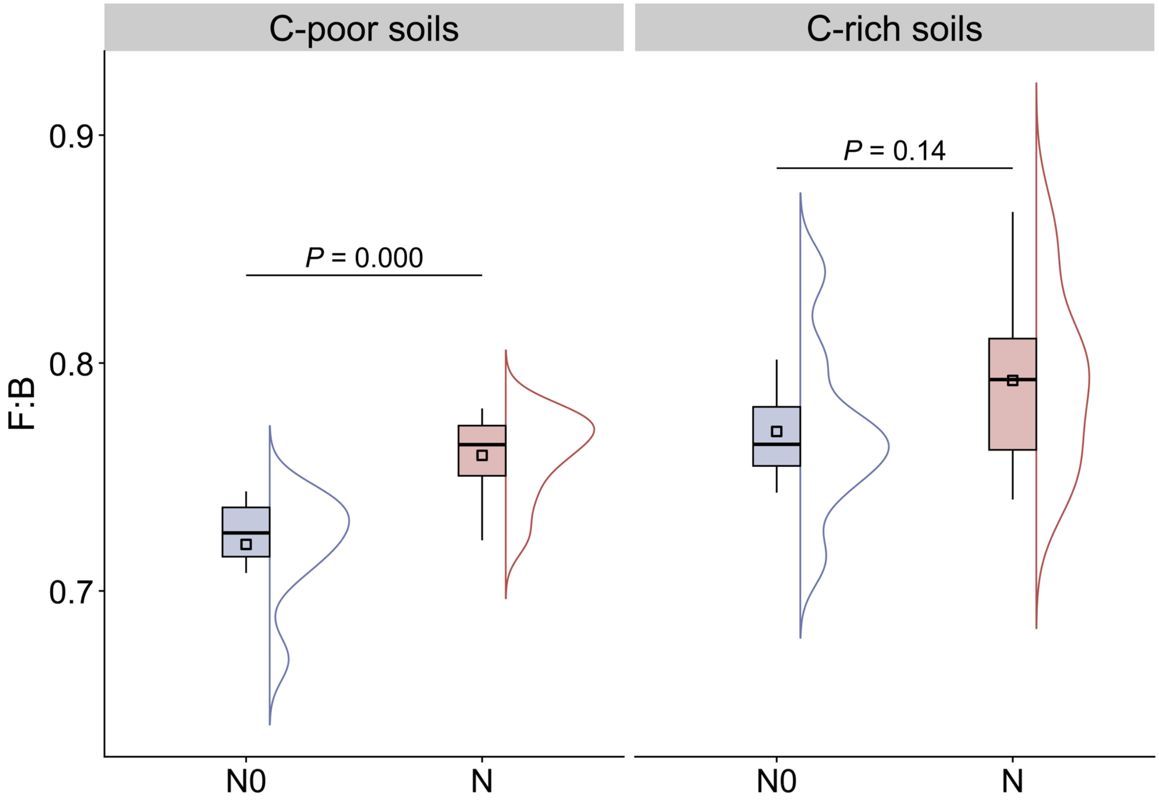

Supplementary Figure 14. The ratio of fungal (F) to bacterial (B) biomass under different N fertilizer treatments between C-poor and C-rich soils.

In box plots, the solid lines and squares in the boxes, the lower and upper edges of the boxes and the lower and upper lines extending from the boxes represent median and mean values (n = 4 for N0, n = 8 for N), Q1 and Q3, 1.5 × IQR above the box's Q1 and 1.5 × IQR below the box's Q3 of all data, respectively. A mixed linear model was used to test the treatment effect at P = 0.05. N0: no N addition; N: N addition.

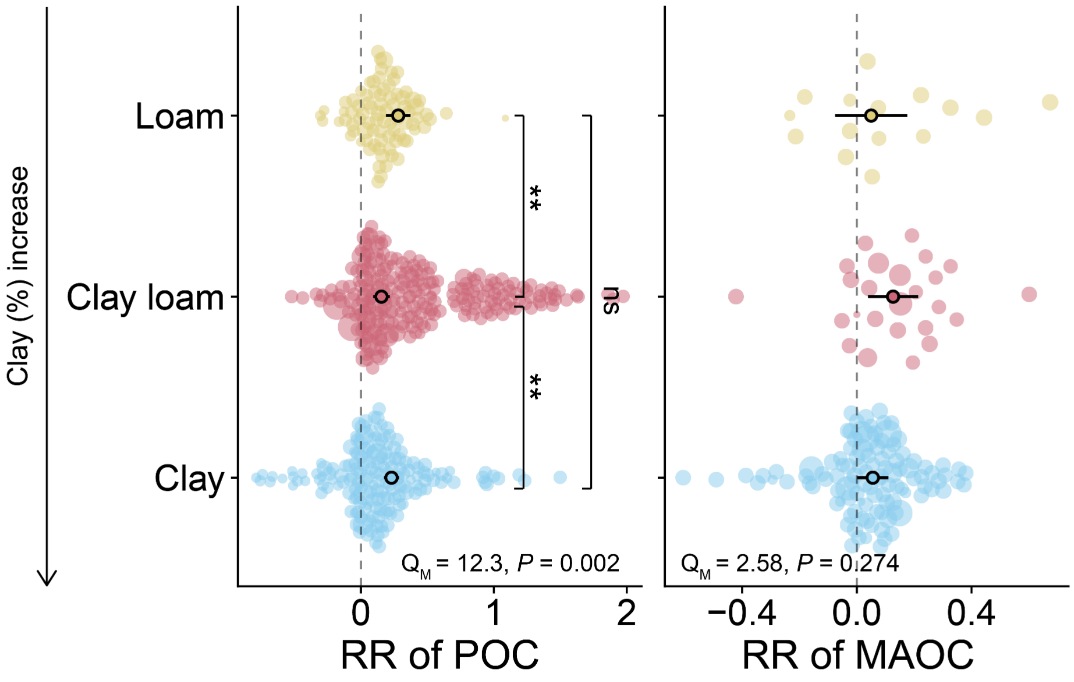

Supplementary Figure 15. Effects of N fertilization on POC and MAOC associated with soil texture.

The between-group heterogeneity (Q_M) statistical test was used to compare the differences in weighted effect sizes among groups divided by the soil texture. A significant QM value ($P < 0.05$) suggested that the weighted effect sizes of a given variable differed among groups. No samples were classified as sandy, resulting in only three observed soil texture categories. An asterisk means there is a significant difference between the two groups, and ns means no difference. * $P < 0.05$; ** $P < 0.01$; *** $P < 0.001$.

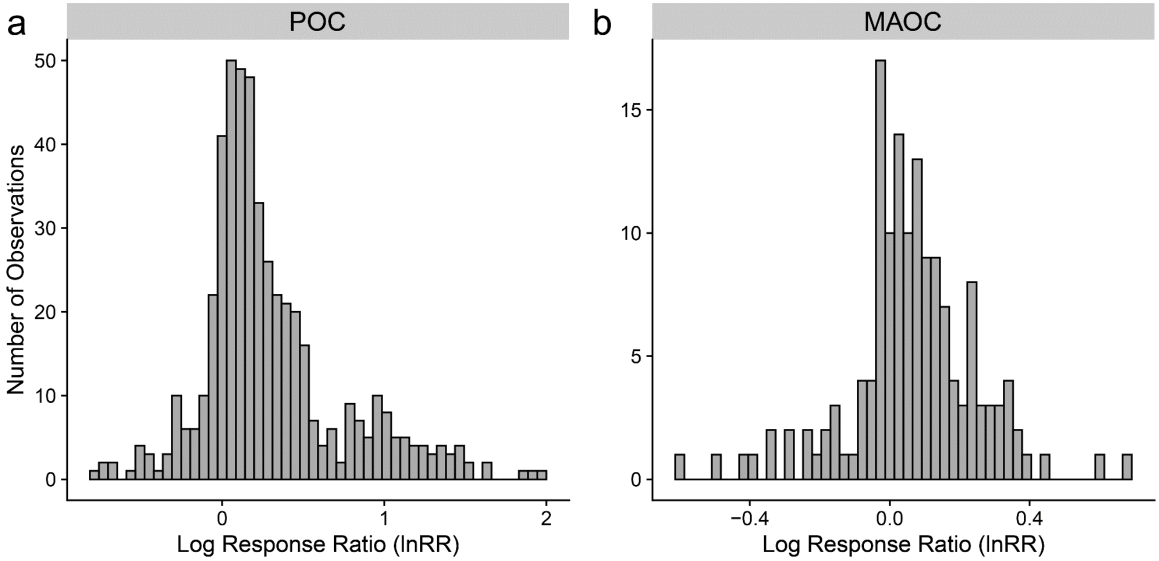

Supplementary Figure 16. Publication bias of POC (a) and MAOC (b) in the datasets.

The histogram showed that the observations were close to normal distribution, and that the meta-analysis was not subject to publication bias. POC: particulate organic carbon; MAOC: mineral-associated organic carbon.

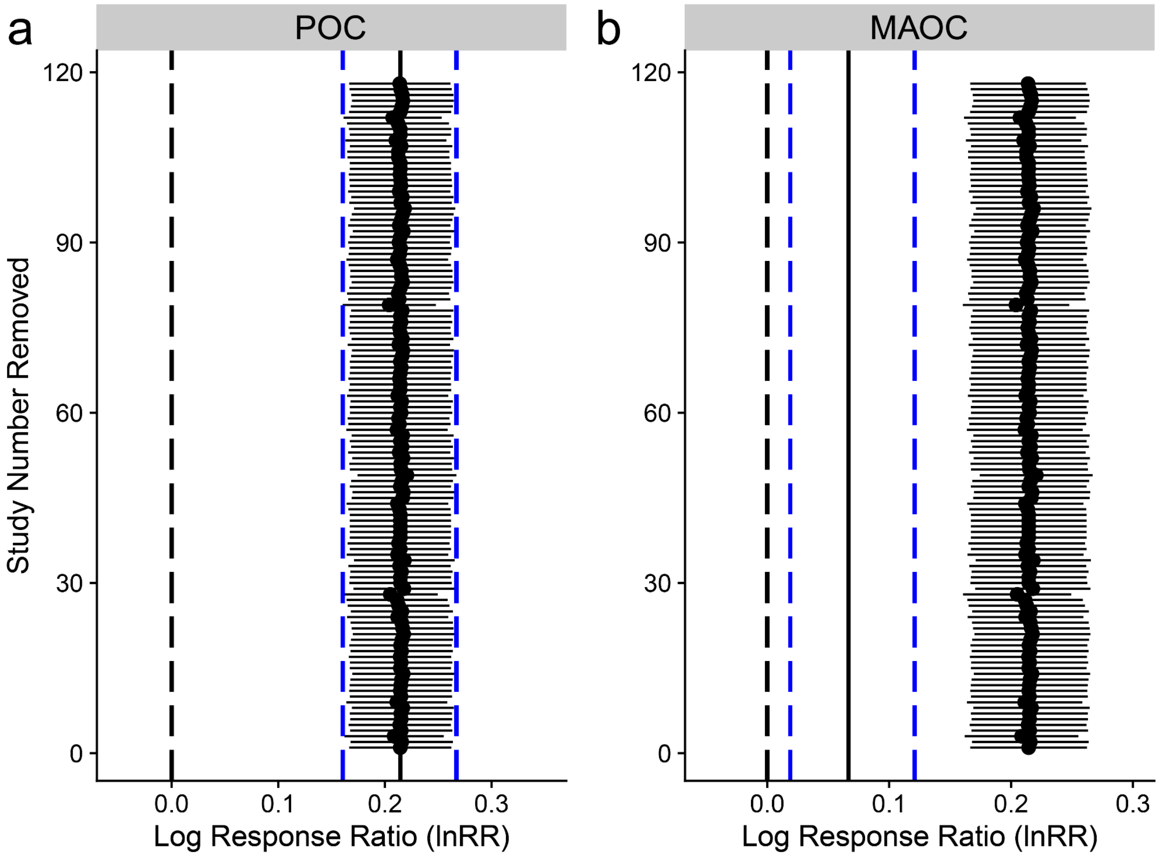

Supplementary Figure 17. The results of the sensitivity analysis for POC (a) and MAOC (b), which was completed using a Jackknife technique.

The solid black line represents the overall lnRR and the lower and higher 95% confidence intervals are also provided as dashed blue lines. The removal of any single study had no effect on the results. POC: particulate organic carbon; MAOC: mineral-associated organic carbon.

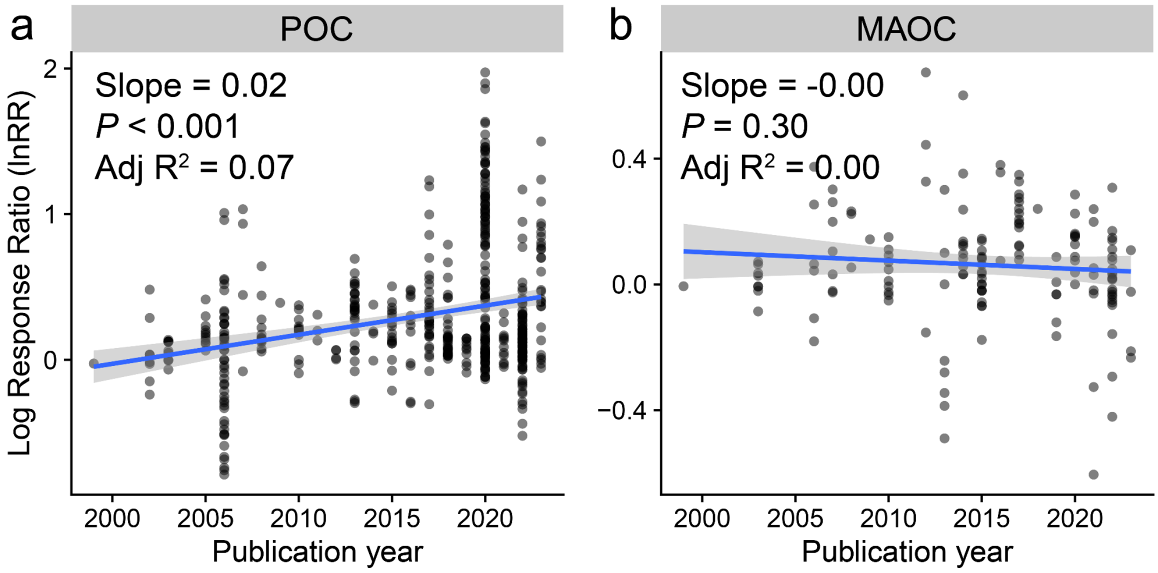

Supplementary Figure 18. Temporal change in effect size of nitrogen fertilizer on POC (a) and MAOC (b) with publication year based on meta-regression analysis.

Linear mixed regression model with two-sided test was used for the statistical analysis, and adjusted R-squared was used. The solid line represents the linear regression, and the gray shading indicates the 95% confidence intervals. POC: particulate organic carbon; MAOC: mineral-associated organic carbon.

Supplementary Table 1. Results of the model selection procedure used to evaluate the response between effect sizes of POC/MAOC and initial SOC.

Variables	model	AICs	ΔAICs	BICs	ΔBICs
POC	**Stegmented**	**491.79**	**0.00**	**519.01**	**0.00**
	M22	522.92	31.13	554.34	35.33
	Step	545.40	53.61	564.21	45.20
	M12	558.60	66.81	585.81	66.80
	Segmented	564.19	72.40	587.20	68.19
	GAM	589.53	97.74	602.13	83.12
MAOC	**Stegmented**	**-100.18**	**0.00**	**-82.28**	**0.00**
	Step	-96.30	3.88	-80.15	2.13
	M22	-92.88	7.30	-76.77	5.51
	M12	-92.56	7.62	-75.48	6.80
	Segmented	-92.50	7.68	-72.54	9.74
	GAM	-85.78	14.4	-69.85	12.43

Bold fonts indicate the best model. The best fitting models showed the lowest AIC and BIC values within a ΔAIC and ΔBIC < 2. POC: particulate organic carbon; MAOC: mineral-associated organic carbon; SOC: soil organic carbon. GAM: generalized additive model; step: two intercept models, with no slope are fitted and the intercept is changed at the breakpoint; segmented: two linear models in which the slope is changed at the breakpoint; stegmented: two linear models in which both the slope and the intercept are changed at the breakpoint; M12: hinge model 12, one linear model is fitted for the left part of the breakpoint and a second-degree polynomial is fitted for the right part; M22: hinge model 22, two different second-degree polynomial models are fitted at both sides of the breakpoint. AIC: Akaike information criteria; BIC: Bayesian information criteria.

Supplementary Table 2. The independent and interactive effect (P values) of initial C conditions (C) and N fertilization input (N) on studied variables.

Variables	Initial SOC (C)		N input (N)		C×N	
	F	*P*	*F*	*P*	*F*	*P*
POC	0.491	0.555	10.8	**< 0.001**	15.1	**< 0.001**
MAOC	120	**0.008**	13.9	**< 0.001**	18.0	**< 0.001**
AGB	1.44	0.353	386	**< 0.001**	0.008	0.991
Lignin phenols	1.71	0.321	12.3	**< 0.001**	31.6	**< 0.001**
Necromass C	1.60	0.333	2.81	0.072	22.7	**< 0.001**
qCO_2	111	0.008	15.5	**< 0.001**	19.9	**< 0.001**
Oxidase	6.81	0.121	30.3	**< 0.001**	14.8	**< 0.001**
F:B	4.15	0.179	7.10	**0.002**	0.88	0.422
Resource limitation	0.16	0.728	3.57	**0.037**	3.91	**0.027**
C limitation	2.68	0.243	0.16	0.849	6.28	**0.004**
MWD	1.94	0.299	38.2	**< 0.001**	56.1	**< 0.001**
$Fe_o + Al_o$	92.4	**0.011**	1.91	0.161	4.23	**0.022**
$Fe_p + Fe_p$	2.70	0.242	37.5	**< 0.001**	39.4	**< 0.001**
Ca_{exe}	12.5	0.071	0.58	0.561	1.41	0.256
Mg_{exe}	1.26	0.379	4.81	**0.013**	1.65	0.206
Aliphaticity	0.05	0.846	0.20	0.822	9.08	**< 0.001**
Recalcitrance	10.8	0.081	0.01	0.987	2.92	0.065

Bold values indicate $P < 0.05$. Linear mixed effect models used Satterthwaite approximation for degrees of freedom. AGB: Aboveground biomass; POC: particulate organic carbon; MAOC: mineral-associated organic carbon; qCO_2: microbial metabolic quotient (respiration-to-biomass ratio); F:B: the ratio of fungal to bacterial biomass; Resources limitation: the ratio of the resource C:N (SOC:TN) ratios normalized to $B_{C:N}$ (MBC:MBN); C limitation: the square root of the sum of x^2 and y^2, where x represents the relative activity of C vs. P-acquiring enzymes, and y represents the relative activity of C vs. N-acquiring enzymes; MWD: mean weight diameter; $Fe_o + Al_o$: poorly crystalline Fe/Al oxides; $Fe_p + Al_p$: organically complexed Fe/Al oxides; Aliphaticity: the ratio of the

alkyl region divided by the O-alkyl region; Recalcitrance: the ratio of (alkyl + aromatic C) to (O-alkyl + carboxyl C).

Supplementary Table 3. Effects of N fertilizer application on activities of oxidative enzymes including polyphenol oxidase (PPO) and peroxidase (POD).

Fertility conditions	Treatments	PPO (nmol h^{-1} g^{-1})	POD (nmol h^{-1} g^{-1})	Oxidase (nmol h^{-1} g^{-1})
C-poor soils	N0	4334 (792)	2759 (350)	82.3 (6.76) a
	N	3976 (368)	1645 (176)	73.9 (3.33) b
C-rich soils	N0	1738 (210) a	800 (94.1) a	50.2 (1.58) a
	N	963 (92.3) b	435 (24.3) b	37.1 (1.24) b

Data are presented as mean and SE (n = 4 for N0, n = 8 for N). Different lower-case letters indicate significant differences between N0 and N within one site at P = 0.05 (Tukey-post-hoc test).The oxidase activity is calculated as the square root of the sum of PPO and POD. PPO: polyphenol activity. POD: Peroxidase activity. Fertility conditions: classified according to the soil organic carbon threshold obtained from the global meta-analysis (i.e., SOC = 15 g kg^{-1}). OPT: optimized N addition; HN: high N addition. C-poor soils: combining QZ and CW results; C-rich soils: combining SP and YA results. N0: no N addition; N: N addition.

Supplementary Table 4. Percentage (%) contribution of C functional groups of bulk soil based on integration of CPMAS ^{13}C NMR spectra.

Fertility conditions	Treatments	Alkyl-C (0-45 ppm)	O-alkyl-C (45-110 ppm)	Aromatic-C (110-160 ppm)	Carbonyl-C (160-220 ppm)	Aliphaticity	Recalcitrance
C-poor soils	N0	23.61 (1.02)	46.70 (0.44) b	17.69 (0.32) b	11.53 (0.4)	0.51 (0.02) a	0.71 (0.02)
	N	22.12 (0.47)	48.67 (0.39) a	18.86 (0.16) a	11.32 (0.65)	0.45 (0.01) b	0.68 (0.01)
C-rich soils	N0	21.10 (0.94) b	46.15 (0.56)	20.78 (0.49)	11.25 (0.54)	0.46 (0.02) b	0.73 (0.01) b
	N	22.94 (0.75) a	45.85 (0.29)	20.16 (0.64)	10.96 (0.20)	0.50 (0.02) a	0.76 (0.01) a

Data are presented as mean and SE (n = 4 for N0, n = 8 for N). Different lower-case letters indicate significant differences between N0 and N within one site at P = 0.05 (Tukey-post- hoc test). The Aliphaticity index is the ratio of the alkyl region divided by the O-alkyl region. The Recalcitrance index is the ratio of (alkyl + aromatic C) to (O-alkyl + carboxyl C). Fertility conditions: classified according to the soil organic carbon threshold obtained from the global meta-analysis (i.e., SOC = 15 g kg^{-1}). C-poor soils: combining QZ and CW results; C-rich soils: combining SP and YA results. N0: no N addition; N: N addition. N0: no N addition; N: N addition.

Supplementary Table 5. Performance of the four machine-learning algorithms tested predicting POC and MAOC changes, including the gradient boosting decision tree model for POC and MAOC in the main text.

**Machine-learning algorithms**	**Variables**	**MAE**	**MSE**	**R^2**
RF	POC	0.17	0.05	0.51
	MAOC	0.11	0.02	0.16
**GBDT**	POC	0.16	0.05	**0.56**
	MAOC	0.08	0.02	**0.28**
XGBoost	POC	0.18	0.06	0.45
	MAOC	NA	NA	NA
LightGBM	POC	0.18	0.06	0.45
	MAOC	0.11	0.03	0.11

Bold indicates the model chosen for best performance and its R^2. RF: random forest; GBDT: gradient-boosting decision trees model; XGBoost: extreme gradient-boosting model; LightGBM: light gradient-boosting machine model. MAE: mean absolute error; MSE: mean squared error. NA indicates that such models are not suitable for modeling and predicting the current dataset, with performance worse than that of a mean model. The optimal model is characterized by the maximum R^2 and the minimum MAE and MSE.

Supplementary Table 6. Summary of covariates used in this study for upscaling our results to a global context.

Group	Covariates	Units	Resolution	Source
Climate	Mean annual temperature (MAT)	℃	30 s	WorldClim, [2]
	Mean annual precipitation (MAP)	mm $year^{-1}$	30 s	
Soil	Soil organic carbon (SOC)	g kg^{-1}	250 m	SoilGrids, [3]
	Total nitrogen (TN)	g kg^{-1}	250 m	
	pH	/	250 m	
	Clay Fraction (Clay)	%	250 m	
	Silt Fraction (Silt)	%	250 m	
Fertilizer	Nitrogen fertilization rate (Ninput)	kg ha^{-1} $year^{-1}$	5 min	PANGAEA, [4]
/	Land use data	/	30 m	[5]

Soil properties from SoilGrids were converted into standardized units based on conversion relationships provided in the official manual to align with our dataset.

Supplementary Table 7. Site description and general description of the experimental design at four sites of nitrogen fertilization treatments.

Sites	Geography	N fertilization rate (Kg N ha^{-1} $year^{-1}$)	Duration of N fertilization (years)	Altitude (m)	Fertility conditions
QuZhou station (QZ)	43.33 N, 124.00 W	N0: 0<br>OPT: 171<br>HN: 250	13	41	C-poor soils
Changwu station (CW)	35.22 N, 107.80 W	N0: 0<br>OPT: 200<br>HN: 400	12	1096	C-poor soils
Siping station (SP)	36.88 N, 115.20 W	N0: 0<br>OPT: 200<br>HN: 312	12	167	C-rich soils
Yaan station (YA)	29.98 N, 103.00 W	N0: 0<br>OPT: 190<br>HN: 381	13	560	C-rich soils

Fertility conditions: classified according to the soil organic carbon threshold obtained from the global meta-analysis (i.e., SOC = 15 g kg^{-1}). C-poor soils: combining QZ and CW results; C-rich soils: combining SP and YA result. OPT: optimized N addition; HN: high N addition.

Supplementary Table 8. A summary of the quality check of this meta-analysis. Quality criteria are derived from Koricheva and Gurevitch.

Quality criteria	This study
1. Has formal meta-analysis been conducted (i.e. combination of effect sizes using standard meta-analytical methodology) or is it simply a vote count?	Yes, this study used natural log response ratio (lnRR) weighted by inverse variance to evaluate the effect size. Vote count was used to map the global distribution.
2. Are details of bibliographic search (electronic data bases used, keyword combinations, years) reported in sufficient detail to allow replication?	Yes, all electronic data (including extracted data and reference list) used in the study are provided, and a PRISMA flow diagram is also provided.
3. Are criteria for study inclusion/exclusion explicitly listed?	Yes, all criteria are listed in the Methods section.
4. Have standard metrics of effect size been used or, if nonstandard metrics have been employed, is the distribution of these parameters known and have the authors explained how they calculated variances for such metrics?	Yes, we used standard metrics of effect size, i.e., Ln(Response Ratio) weighted by inverse variance, and effect size is also calculated.
5. If more than one estimate of effect size per study was included in the analysis, has potential non-independence of these estimates been taken into account?	Yes, we performed a sensitivity analysis to assess the reliability of the results
6. Have effect sizes been weighted by study precision or has the rational for using unweighted approach been provided?	Yes, effect sizes were weighted by the inverse variance.
7. Have statistical model for meta-analysis and the software used been described?	Yes, we calculated the weighted mean of lnRR in a mixed-effects model using the "metafor" paN0age in R version 4.2.1.
8. Has heterogeneity of effect sizes between studies been quantified?	Yes, confidence interval was given in all meta-analyses, and the accumulated percentage of effect sizes were shown in Fig. 5.
9. Have the causes of existent heterogeneity in effect sizes been explored by meta-regression?	Yes, grouping experiments were used.
10. If effects of multiple moderators have been tested, have potential non-independence of and interactions between moderators been taken into account?	Yes, both potential non-independence of and interactions between moderators were taken into account by the *glmuti* paN0ages in R.
11. If meta-analysis combined studies conducted on different species, has phylogenetic relatedness of species been taken into account?	Not applicable to this study.
12. Have tests for publication bias been conducted?	Yes, we carried out egger test and drew the histogram plot.
13. If meta-analysis combines studies published over considerable time span, have possible temporal changes in effect size been tested?	Yes, possible temporal changes in effect size were tested using a meta-regression method, as shown in Supplementary Fig. 7.
14. Have sensitivity analysis been performed to test the robustness of results?	Yes, the jaN0knife technique was run to test the robustness of results, as show in Supplementary Fig. 6.
15. Have full bibliographic details of primary studies included in a meta-analysis been provided?	Yes, the full bibliographic details of primary studies are deposited in a public repository.
16. Has the data set used for meta-analysis, including effect sizes and variances/sample sizes from individual primary studies and moderator variables, been provided as electronic appendix?	Yes, all the data are deposited in a public repository.

Supplementary References

1. Peng, Q. *et al.* A twenty-year dataset of high-resolution maize distribution in China. *Sci Data* **10**, 658 (2023).
2. Fick, S. E. & Hijmans, R. J. WorldClim 2: new 1-km spatial resolution climate surfaces for global land areas. *Int. J. Climatol.* **37**, 4302–4315 (2017).
3. Poggio, L. *et al.* SoilGrids 2.0: producing soil information for the globe with quantified spatial uncertainty. *Soil* **7**, 217–240 (2021).
4. Zhang, B. *et al.* Global manure nitrogen production and application in cropland during 1860–2014: a 5 arcmin gridded global dataset for Earth system modeling. *Earth Syst. Sci. Data* **9**, 667–678 (2017).
5. Potapov, P. *et al.* Global maps of cropland extent and change show accelerated cropland expansion in the twenty-first century. *Nat. Food* **3**, 19–28 (2022).

Meta-analysis database references (until June 2024)
Web of Sciences and Google Scholar

1. Yan, M., Zhang, X., Liu, K., Lou, Y. & Wang, Y. Particle size primarily shifts chemical composition of organic matter under long-term fertilization in paddy soil. *Eur. J. Soil Sci. e***73**, e13170 (2022).
2. Hao, X., Han, X., Wang, S. & Li, L.-J. Dynamics and composition of soil organic carbon in response to 15 years of straw return in a Mollisol. *SOIL TILL RES* **215**, 105221 (2022).
3. Ge, Z. *et al.* Differential long-term fertilization alters residue-derived labile organic carbon fractions and microbial community during straw residue decomposition. *SOIL TILL RES* **213**, 105120 (2021).
4. Dai, W. *et al.* Changes in soil organic carbon fractions in response to wheat straw incorporation in a subtropical paddy field in China. *J. Plant Nutr. Soil Sci.* **184**, 198–207 (2021).
5. Liliana, V. J., Gutierrez Boem, F., Garcia, F. & Rubio, G. Long-term fertilization does not affect soil carbon/nitrogen/sulfur ratios or the proportion between labile and nonlabile fractions in Mollisols. *Soil Sci. Soc. Am. J.* **84**, 798–810 (2020).
6. Chen, X. *et al.* Soil microbial communities under wheat and maize straw incorporation are closely associated with soil organic carbon fractions and chemical structure. *Appl. Soil Ecol.* **182**, 104724 (2023).
7. Ren, G. *et al.* Soil organic carbon and nitrogen fractions as affected by straw and nitrogen management on the North China Plain. *AGR ECOSYST ENVIRON* **342**, 108248 (2023).
8. Mikha, M. M. & Marake, M. V. Soil organic matter fractions and carbon distribution under different management in Lesotho, southern Africa. *Soil Sci. Soc. Am. J.* **87**, 140–155 (2023).
9. Liu, B. *et al.* 14 year applications of chemical fertilizers and crop straw effects on soil labile organic carbon fractions, enzyme activities and microbial community in rice-wheat rotation of middle China. *Sci. Total Environ.* **841**, 156608 (2022).
10. Anandakumar, S. *et al.* Impact of long-term nutrient management on sequestration and dynamics of soil organic carbon in a semi-arid tropical Alfisol of India. *Appl. Soil Ecol.* **177**, 104549 (2022).
11. Abagandura, G. O. *et al.* Soil labile carbon and nitrogen fractions after eleven years of manure and mineral fertilizer applications. *Arch. Agron. Soil Sci.* **69**, 875–890 (2023).
12. Anantha, K. C. *et al.* Distribution of sequestered carbon in different pools in Alfisols under long-term groundnut system of hot arid region of India. *Eur. J. Agron.* **135**, 126467 (2022).
13. Jaswal, A., Mehta, C. M. & Singh, A. Probing the impact of biochar combined with organic and inorganic amendments on soil carbon pools of rice (*Oryza sativa*) - wheat (*Triticum aestivum*) cropping system. *RES CROP* **23**, 508–515 (2022).
14. Sithole, N. J., Magwaza, L. S. & Thibaud, G. R. Long-term impact of no-till conservation agriculture and N-fertilizer on soil aggregate stability, infiltration and distribution of C in different size fractions. *SOIL TILL RES* **190**, 147–156 (2019).
15. Li, Z. *et al.* Effects of straw management and nitrogen application rate on soil organic matter fractions and microbial properties in North China Plain. *J. Soils Sediments* **19**, 618–628 (2019).

16. Hazra, K. K. *et al.* Improving soil organic carbon pools through inclusion of summer mungbean in cereal-cereal cropping systems in Indo-Gangetic plain. *Arch. Agron. Soil Sci.* **64**, 1690–1704 (2018).
17. Li, J. *et al.* Soil labile organic carbon fractions and soil organic carbon stocks as affected by long-term organic and mineral fertilization regimes in the North China Plain. *SOIL TILL RES* **175**, 281–290 (2018).
18. Joshi, S. K. *et al.* Soil organic carbon dynamics in a Chhattisgarh vertisol after use of a rice–wheat system for 16 years. *Agron. J.* **109**, 2556–2569 (2017).
19. Wei, Z., Ji, J., Li, Z. & Yan, X. Changes in organic carbon content and its physical and chemical distribution in paddy soils cultivated under different fertilisation practices. *J. Soils Sediments* **17**, 2011–2018 (2017).
20. Phalke, D. H., Patil, S. R., Manna, M. C., Mandal, A. & Pharande, A. L. Effect of in-situ recycling of sugarcane crop residues and its industrial wastes on different soil carbon pools under soybean (*Glycine max*) - maize (*Zea mays*) system. *INDIAN J AGR SCI* **87**, (2017).
21. Jiang, M. *et al.* Variation of soil aggregation and intra-aggregate carbon by long-term fertilization with aggregate formation in a grey desert soil. *CATENA* **149**, 437–445 (2017).
22. Hua, K., Wang, D. & Guo, Z. Effects of long-term application of various organic amendments on soil particulate organic matter storage and chemical stabilisation of vertisol soil. *ACTA AGR SCAND B-S P* **68**, 505–514 (2018).
23. Qiu, S. *et al.* Changes in soil carbon and nitrogen pools in a Mollisol after long-term fallow or application of chemical fertilizers, straw or manures. *SOIL TILL RES* **163**, 255–265 (2016).
24. Wei, Z., Wu, J., Yan, X. & Ni, G. Phosphorus and carbon status of a paddy soil under different fertilization regimes. *J. Soils Sediments* **16**, 1727–1734 (2016).
25. Wang, Y. *et al.* 23-year manure and fertilizer application increases soil organic carbon sequestration of a rice–barley cropping system. *Biol. Fertil. Soils* **51**, 583–591 (2015).
26. Sindelar, A. J., Lamb, J. A. & Coulter, J. A. Short-term stover, tillage, and nitrogen management affect near-surface soil organic matter. *Soil Sci. Soc. Am. J.* **79**, 251–260 (2015).
27. Das, B. *et al.* Effect of integrated nutrient management practice on soil aggregate properties, its stability and aggregate-associated carbon content in an intensive rice–wheat system. *SOIL TILL RES* **136**, 9–18 (2014).
28. Tong, X. *et al.* Long-term fertilization effects on organic carbon fractions in a red soil of China. *CATENA* **113**, 251–259 (2014).
29. Aulakh, M. S., Garg, A. K. & Kumar, S. Impact of integrated nutrient, crop residue and tillage management on soil aggregates and organic matter fractions in semiarid subtropical soil under soybean-wheat rotation. *Am. J. Plant Sci* **04**, 2148–2164 (2013).
30. Wang, Q. *et al.* The effects of compost in a rice–wheat cropping system on aggregate size, carbon and nitrogen content of the size–density fraction and chemical composition of soil organic matter, as shown by ^{13}C CP NMR spectroscopy. *Soil Use Manage.* **28**, 337–346 (2012).
31. Hai, L., Li, X. G., Li, F. M., Suo, D. R. & Guggenberger, G. Long-term fertilization and manuring effects on physically-separated soil organic matter pools under a wheat–

wheat–maize cropping system in an arid region of China. *SOIL BIOL BIOCHEM* **42**, 253–259 (2010).
32. Ouédraogo, E., Mando, A. & Stroosnijder, L. Effects of tillage, organic resources and nitrogen fertiliser on soil carbon dynamics and crop nitrogen uptake in semi-arid West Africa. *SOIL TILL RES* **91**, 57–67 (2006).
33. Yan, X. *et al.* Carbon sequestration efficiency in paddy soil and upland soil under long-term fertilization in southern China. *SOIL TILL RES* **130**, 42–51 (2013).
34. Yang, C., Yang, L. & Ouyang, Z. Organic carbon and its fractions in paddy soil as affected by different nutrient and water regimes. *GEODERMA* **124**, 133–142 (2005).
35. Kapkiyai, J. J., Karanja, N. K., Qureshi, J. N., Smithson, P. C. & Woomer, P. L. Soil organic matter and nutrient dynamics in a Kenyan nitisol under long-term fertilizer and organic input management. *SOIL BIOL BIOCHEM* **31**, 1773–1782 (1999).
36. Mando, A., Bonzi, M., Wopereis, M. C. S., Lompo, F. & Stroosnijder, L. Long-term effects of mineral and organic fertilization on soil organic matter fractions and sorghum yield under Sudano-Sahelian conditions. *Soil Use Manage.* **21**, 396–401 (2005).
37. Fungo, B. *et al.* Aggregate size distribution in a biochar-amended tropical Ultisol under conventional hand-hoe tillage. *SOIL TILL RES* **165**, 190–197 (2017).
38. Roohi, M. *et al.* Role of fertilization regime on soil carbon sequestration and crop yield in a maize-cowpea intercropping system on low fertility soils. *GEODERMA* **428**, 116152 (2022).
39. Rahman, M. M. *et al.* Biochar and compost-based integrated nutrient management: potential for carbon and microbial enrichment in degraded acidic and Charland soils. *Front. Environ. Sci.* **09**, 798729 (2022).
40. Hwang, H. Y. *et al.* Soil labile organic carbon fractions and carbon management index in response to different fertilization under organic maize farming system. *Korean J. Soil. Sci. Fert.* **55**, 522–532 (2022).
41. Kauer, K., Pärnpuu, S., Talgre, L., Eremeev, V. & Luik, A. Soil particulate and mineral-associated organic matter increases in organic farming under cover cropping and manure addition. *Agriculture* **11**, 903 (2021).
42. Nisar, S. & Benbi, D. K. Stabilization of organic C in an Indo-Gangetic alluvial soil under long-term manure and compost management in a rice–wheat system. *Carbon Manage.* **11**, 533–547 (2020).
43. Wuaden, C. R., Nicoloso, R. S., Barros, E. C. & Grave, R. A. Early adoption of no-till mitigates soil organic carbon and nitrogen losses due to land use change. *SOIL TILL RES* **204**, 104728 (2020).
44. Verma, R. K., Shivay, Y. S., Prasanna, R., Choudhary, M. & Ghasal, P. C. Nutrient management options modulating soil physico-chemical and biological properties under direct-seeded rice-based cropping systems. *Arch. Agron. Soil Sci.* **67**, 1783–1798 (2021).
45. Tang, H. *et al.* Short-term responses of soil organic carbon and its labile fractions to different manure Nitrogen input in a double-cropping rice field. *J. Agric. Sci.* **158**, 119–127 (2020).
46. Mafra, M. S. H. *et al.* Organic carbon contents and stocks in particle size fractions of a typic hapludox fertilized with pig slurry and soluble fertilizer. *REV BRAS CIENC SOLO* **39**, 1161–1171 (2015).

47. Singh, A., Gulati, I. J., Chopra, R., Sharma, D. & Gochar, R. Effect of drip-fertigation with organic manures on soil properties and tomato (*lycopersicon esculentum mill.*) yield under arid condition. *Annals of Biology* **30**, 345–349 (2014).
48. Yu, H., Ding, W., Luo, J., Geng, R. & Cai, Z. Long-term application of organic manure and mineral fertilizers on aggregation and aggregate-associated carbon in a sandy loam soil. *SOIL TILL RES* **124**, 170–177 (2012).
49. Wienhold, B. J. Changes in soil attributes following low phosphorus swine slurry application to no-tillage sorghum. *Soil Sci. Soc. Am. J.* **69**, 206 (2005).
50. Huang, Y. *et al.* Effects of substitution of chemical fertilizer by Chinese milk vetch on distribution and composition of aggregates-associated organic carbon fractions in paddy soils. *Plant Soil* **481**, 641–659 (2022).
51. Yan, Z. *et al.* Changes in soil organic carbon stocks from reducing irrigation can be offset by applying organic fertilizer in the North China Plain. *Agric. Water Manage.* **266**, 107539 (2022).
52. Xiang, Y., Cheng, M., Wen, Y. & Darboux, F. Soil organic carbon sequestration under long-term chemical and manure fertilization in a cinnamon soil, Northern China. *Sustainability* **14**, 5109 (2022).
53. Lan, X. *et al.* Effects of long-term manure substitution regimes on soil organic carbon composition in a red paddy soil of southern China. *SOIL TILL RES* **221**, 105395 (2022).
54. Kim, K., Daly, E. J., Gorzelak, M. & Hernandez-Ramirez, G. Soil organic matter pools response to perennial grain cropping and nitrogen fertilizer. *SOIL TILL RES* **220**, 105376 (2022).
55. Qaswar, M. *et al.* Dynamics of organic carbon and nitrogen in deep soil profile and crop yields under long-term fertilization in wheat-maize cropping system. *J. Integr. Agric.* **21**, 826–839 (2022).
56. Li, H., Han, X., You, M. & Xing, B. Organic matter associated with soil aggregate fractions of a black soil in Northeast China: impacts of land-use change and long-term fertilization. *Commun. Soil Sci. Plant Anal.* **46**, 405–423 (2015).
57. Wang, R. *et al.* Changes in soil organic carbon pools following long-term fertilization under a rain-fed cropping system in the Loess Plateau, China. *J. Integr. Agric.* **20**, 2512–2525 (2021).
58. Duan, Y. *et al.* Long-term fertilisation reveals close associations between soil organic carbon composition and microbial traits at aggregate scales. *AGR ECOSYST ENVIRON* **306**, 107169 (2021).
59. Pant, P. K., Ram, S., Bhatt, P., Mishra, A. & Singh, V. Vertical distribution of different pools of soil organic carbon under long-term fertilizer experiment on rice-wheat sequence in mollisols of North India. *Commun. Soil Sci. Plant Anal.* **52**, 235–255 (2021).
60. Chen, J. *et al.* Composition, predicted functions, and co-occurrence networks of fungal and bacterial communities_ Links to soil organic carbon under long-term fertilization in a rice-wheat cropping system. *Eur. J. Soil Biol.* **100**, 103226 (2020).
61. Zhao, Z., Gao, S., Lu, C., Li, X. & Wang, T. Soil organic carbon fractions and its association with water-stable aggregates under different fertilization management practices. *Soil Use Manage.* **38**, 478–486 (2022).

62. Duval, M. E., Martinez, J. M. & Galantini, J. A. Assessing soil quality indices based on soil organic carbon fractions in different long-term wheat systems under semiarid conditions. *Soil Use Manage.* **36**, 71–82 (2020).
63. Mandal, N., Dwivedi, B. S., Datta, S. P., Meena, M. C. & Tomar, R. K. Soil hydrophysical properties under different nutrient management practices, their relationship with soil organic carbon fractions and crop yield under pigeonpea-wheat sequence. *J. Plant Nutr.* **42**, 384–400 (2019).
64. Sarma, B. & Gogoi, N. Nitrogen management for sustainable soil organic carbon increase in inceptisols under wheat cultivation. *Commun. Soil Sci. Plant Anal.* **48**, 1428–1437 (2017).
65. Qi, R. *et al.* Temperature effects on soil organic carbon, soil labile organic carbon fractions, and soil enzyme activities under long-term fertilization regimes. *Appl. Soil Ecol.* **102**, 36–45 (2016).
66. Cai, A. *et al.* Carbon and nitrogen mineralization in relation to soil particle-size fractions after 32 years of chemical and manure application in a continuous maize cropping system. *PLoS One* **11**, e0152521 (2016).
67. Thomas, B. W., Whalen, J. K., Sharifi, M., Chantigny, M. & Zebarth, B. J. Labile organic matter fractions as early-season nitrogen supply indicators in manure-amended soils. *J. Plant Nutr. Soil Sci.* **179**, 94–103 (2016).
68. Chai, Y. *et al.* Long-term fertilization effects on soil organic carbon stocks in the irrigated desert soil of NW China. *J. Plant Nutr. Soil Sci.* **178**, 622–630 (2015).
69. Sarma, B., Bhattacharya, S., Gogoi, N., Paul, S. & Baroowa, B. Impact of N fertilization on C balance and soil quality in maize-dhaincha cropping sequence. *Journal of Agricultural Sciences (Belgrade)* **60**, 135–148 (2015).
70. Xie, H. *et al.* Long-term manure amendments enhance neutral sugar accumulation in bulk soil and particulate organic matter in a Mollisol. *SOIL BIOL BIOCHEM* **78**, 45–53 (2014).
71. Tian, J., Lu, S., Fan, M., Li, X. & Kuzyakov, Y. Integrated management systems and N fertilization: effect on soil organic matter in rice-rapeseed rotation. *Plant Soil* **372**, 53–63 (2013).
72. Manna, M. C. *et al.* Carbon fractions and productivity under changed climate scenario in soybean–wheat system. *Field Crops Res.* **145**, 10–20 (2013).
73. Mikha, M. M., Hergert, G. W., Benjamin, J. G., Jabro, J. D. & Nielsen, R. A. Soil organic carbon and nitrogen in long-term manure management system. *Soil Sci. Soc. Am. J.* **81**, 153–165 (2017).
74. Banashree, S., Smrita, B., Nath, D. J. & Nirmali, G. Temporal responses of soil biological characteristics to organic inputs and mineral fertilizers under wheat cultivation in inceptisol. *Arch. Agron. Soil Sci.* **63**, 35–47 (2017).
75. Lou, Y., Wang, J. & Liang, W. Impacts of 22-year organic and inorganic N managements on soil organic C fractions in a maize field, Northeast China. *CATENA* **87**, 386–390 (2011).
76. Lou, Y., Xu, M., Wang, W., Sun, X. & Liang, C. Soil organic carbon fractions and management index after 20 yr of manure and fertilizer application for greenhouse vegetables. *Soil Use Manage.* **27**, 163–169 (2011).

77. Beedy, T. L., Snapp, S. S., Akinnifesi, F. K. & Sileshi, G. W. Impact of Gliricidia sepium intercropping on soil organic matter fractions in a maize-based cropping system. *AGR ECOSYST ENVIRON* **138**, 139–146 (2010).
78. Banger, K., Toor, G. S., Biswas, A., Sidhu, S. S. & Sudhir, K. Soil organic carbon fractions after 16-years of applications of fertilizers and organic manure in a Typic Rhodalfs in semi-arid tropics. *Nutr. Cycling Agroecosyst.* **86**, 391–399 (2010).
79. Huang, S., Peng, X., Huang, Q. & Zhang, W. Soil aggregation and organic carbon fractions affected by long-term fertilization in a red soil of subtropical China. *GEODERMA* **154**, 364–369 (2010).
80. Huang, S., Rui, W., Peng, X., Huang, Q. & Zhang, W. Organic carbon fractions affected by long-term fertilization in a subtropical paddy soil. *Nutr. Cycling Agroecosyst.* **86**, 153–160 (2010).
81. Leifeld, J., Reiser, R. & Oberholzer, H. Consequences of conventional versus organic farming on soil carbon: results from a 27-year field experiment. *Agron. J.* **101**, 1204–1218 (2009).
82. Zhe E, S. *et al.* Long-term fertilization and manuring effects on physically separated soil organic-matter pools under continuous wheat cropping at a rainfed semiarid site in China. *J. Soil Sci. Plant Nutr.* **175**, 689–697 (2012).
83. Manna, M., Swarup, A., Wanjari, R., Mishra, B. & Shahi, D. Long-term fertilization, manure and liming effects on soil organic matter and crop yields. *SOIL TILL RES* **94**, 397–409 (2007).
84. Manna, M. C., Swarup, A., Wanjari, R. H. & Ravankar, H. N. Long-term effects of NPK fertiliser and manure on soil fertility and a sorghum-wheat farming system. *AUST J EXP AGR* **47**, 700 (2007).
85. Rudrappa, L., Purakayastha, T. J., Singh, D. & Bhadraray, S. Long-term manuring and fertilization effects on soil organic carbon pools in a Typic Haplustept of semi-arid sub-tropical India. *SOIL TILL RES* **88**, 180–192 (2006).
86. Galantini, J. & Rosell, R. Long-term fertilization effects on soil organic matter quality and dynamics under different production systems in semiarid Pampean soils. *SOIL TILL RES* **87**, 72–79 (2006).
87. Manna, M. C. *et al.* Soil organic matter in a west bengal inceptisol after 30 years of multiple cropping and fertilization. *Soil Sci. Soc. Am. J.* **70**, 121–129 (2006).
88. Fabrizzi, K. P., Morón, A. & García, F. O. Soil carbon and nitrogen organic fractions in degraded vs. non-degraded Mollisols in Argentina. *Soil Sci. Soc. Am. J.* **67**, 1831–1841 (2003).
89. Sainju, U. M., Whitehead, W. F. & Singh, B. P. Cover crops and nitrogen fertilization effects on soil aggregation and carbon and nitrogen pools. *Can. J. Soil. Sci.* **83**, 155–165 (2003).
90. Liebig, M. A., Varvel, G. E., Doran, J. W. & Wienhold, B. J. Crop sequence and nitrogen fertilization effects on soil properties in the western corn belt. *Soil Sci. Soc. Am. J.* **66**, 596–601 (2002).
91. Purakayastha, T. J., Rudrappa, L., Singh, D., Swarup, A. & Bhadraray, S. Long-term impact of fertilizers on soil organic carbon pools and sequestration rates in maize–wheat–cowpea cropping system. *GEODERMA* **144**, 370–378 (2008).
92. Sharifi, M., Lynch, D. H., Hammermeister, A., Burton, D. L. & Messiga, A. J. Effect of green manure and supplemental fertility amendments on selected soil quality

parameters in an organic potato rotation in Eastern Canada. *Nutr. Cycling Agroecosyst.* **100**, 135–146 (2014).
93. Samson, M.E. *et al.* Management practices differently affect particulate and mineral-associated organic matter and their precursors in arable soils. *SOIL BIOL BIOCHEM* **148**, 107867 (2020).
94. Cao, B. *et al.* Long-term nitrogen and straw application improves wheat production and soil organic carbon sequestration. *J. Soil Sci. Plant Nutr.* **22**, 3364–3376 (2022).
95. He, Y. T. *et al.* Long-term combined chemical and manure fertilizations increase soil organic carbon and total nitrogen in aggregate fractions at three typical cropland soils in China. *Sci. Total Environ.* **532**, 635–644 (2015).
96. Prasad, J. V. N. S. *et al.* Effect of ten years of reduced tillage and recycling of organic matter on crop yields, soil organic carbon and its fractions in Alfisols of semi arid tropics of southern India. *SOIL TILL RES* **156**, 131–139 (2016).
97. Zou, Z. *et al.* Decadal application of mineral fertilizers alters the molecular composition and origins of organic matter in particulate and mineral-associated fractions. *SOIL BIOL BIOCHEM* **182**, 109042 (2023).

CNKI

98. Cai, Y. *et al.* Effects of continuous straw returning with chemical fertilizer on the carbon pool and crop yield of rice-rape rotation soils. *Environmental Science* **43**, 4716–4724 (2022).
99. Fan, Z., Gao, H.W., Wu, J.F, Wei, Z.Q. & Yan, X. The response of organic carbon and its physical and chemical components to fertilization in red paddy soil. *Acta Agriculturae Universitatis Jiangxiensis* **43**, 214–222 (2021).
100. Fang, K. *et al.* Effects of long-term spent straw incorporation on organic carbons in soil and carbon pool management at two-crop rice fields. *Fujian Journal of Agricultural Sciences* **37**, 1216–1224 (2022).
101. Zhou, P., Zhang, X.H. & Pan, G.X. Effect of long-term fertilization on content of total and particulate organic carbon and their depth distribution of a paddy soil: an example of Huangnitu from the tai lake region China. *Plant Nutrition and Fertilizer Science* **12**, 765–771 (2006).
102. Di, Y.J. *et al.* Influences of long-term organic and chemical fertilization on soil aggregation and associated organic carbon fractions in a red paddy soil. *Chinese Journal of Eco-Agriculture* **22**, 1129–1138 (2014).
103. Hua, K.K., Zhu, B., Yang, X.L. & Wang, X.G. Effects of long-term different fertilization on soil aggregates and organic carbon fractions on sloping upland of purple soil. *Transactions of the Chinese Society for Agricultural Machinery* **45**, 167–174 (2014).
104. Kang, G.D. *et al.* Effects of organic material application on crop yield and active organic components in upland red soil. *Soils* **49**, 1084–1091 (2017).
105. He, M. *et al.* Response of the active carbon pool and enzymatic activity of soils to maize straw returning. *Journal of Agro-Environment Science* **37**, 1942–1951 (2018).
106. Li, F. *et al.* Effects of Chinese milk vetch and straw returning on soil nutrient and active organic carbon under reduced application of chemical fertilizer. *Journal of Huazhong Agricultural University* **39**, 67–75 (2020).

107. Wang, X.J. *et al.* Effects of alternative fertilization practices on components of the soil organic carbon pool and yield stability in rain-fed maize production on the Loess Plateau. *Acta Pedologica Sinica* **29**, 58–69 (2020).
108. Wan, Y.R., Han, X.Z., Li, N., You, M.Y. & Zou, W.X. Effects of different improvement regimes on organic carbon pools of soils newly developed from parent material of Mollisols. *Chinese Journal Ecology* **39**, 1175–1182 (2020).
109. Kou, Z.R, Zhou, X.B., Xu, C. & Xie, D. Effects of combination application of organic and inorganic fertilizers on organic carbon fractions in tobacco-planting yellow soil. *Soils* **52**, 195–201 (2020).
110. He, W. *et al.* Effect of long-term application of organic manure expanding organic carbon fractions in fluvo-aquic soil. *Acta Pedologica Sinica* **57**, 425–434 (2020).
111. Zhang, J.M. *et al.* Effects of different proportions of labile organic fertilizer substituted by nitrogen fertilizer on components of soil organic carbon. *Journal of Triticeae Crops* **41**, 1534–1540 (2021).
112. Sun, X.H., Niu, J.Y. & Zhao, G. Effects of previous crop stubbles and nitrogen applied amount on summer maize yield and soil organic carbon pool at a semiarid loess site of China. *Agricultural Research in the Arid Areas* **36**, 19–27 (2018).
113. Zhao, Y.H. *et al.* Soil organic carbon stock and active carbon fractions under four kinds of long-term fertilization. *Chinese Journal of Ecology* **35**, 1826–1833 (2016).
114. Hu, K. *et al.* Effects of tobacco stalk biochar-based fertilizer on the organic carbon fractions and microbial community structure of adlay soil. *Chinese Journal of Eco-Agriculture* **29**, 1592–1603 (2021).
115. Liu, Y. *et al.* Evolution characteristics of organic carbon fractions in gray desert soil under long-term fertilization. *Plant Nutrition and Fertilizer Science* **16**, 794–800 (2010).
116. Gong, W. *et al.* Effects of long-term fertilization on soil particulate organic carbon and nitrogen in a wheat-maize cropping system. *Chinese Journal of Applied Ecology* **11**, 2375–2381 (2008).
117. Yang, C.M., Ouyang, Z. & Dong, Y.H. Organic carbon fractions and aggregate stability in aquatic soil under different fertilization. *Chinese Journal of Ecology* **24**, 887–892 (2005).
118. Gao, M.Y., Jiang, T., Han, X.R. & Yang, J.F. Effects of applying biochar-based fertilizer and biochar on organic carbon fractions and contents of brown soil. *Scientia Agricultura Sinica* **51**, 2126–2135 (2018).
