# Supplementary material for: Soil organic carbon thresholds control fertilizer effects on carbon accrual in croplands worldwide
Source: Nat Commun. 2025 Mar 27;16:3009. doi: 10.1038/s41467-025-57981-6 (PMC11950326; doi:10.1038/s41467-025-57981-6)
Supplement: Supplementary file 3 — Reporting Summary [file 41467_2025_57981_MOESM3_ESM.pdf]

nature portfolio

Corresponding author(s): Xinping Chen, Jing Tian

Last updated by author(s): Feb 23, 2025

Reporting Summary

Nature Portfolio wishes to improve the reproducibility of the work that we publish. This form provides structure for consistency and transparency in reporting. For further information on Nature Portfolio policies, see our Editorial Policies and the Editorial Policy Checklist.

Statistics

For all statistical analyses, confirm that the following items are present in the figure legend, table legend, main text, or Methods section.

n/a	Confirmed	
☐	☒	The exact sample size (*n*) for each experimental group/condition, given as a discrete number and unit of measurement
☐	☒	A statement on whether measurements were taken from distinct samples or whether the same sample was measured repeatedly
☐	☒	The statistical test(s) used AND whether they are one- or two-sided<br>*Only common tests should be described solely by name; describe more complex techniques in the Methods section.*
☐	☒	A description of all covariates tested
☐	☒	A description of any assumptions or corrections, such as tests of normality and adjustment for multiple comparisons
☐	☒	A full description of the statistical parameters including central tendency (e.g. means) or other basic estimates (e.g. regression coefficient) AND variation (e.g. standard deviation) or associated estimates of uncertainty (e.g. confidence intervals)
☐	☒	For null hypothesis testing, the test statistic (e.g. *F*, *t*, *r*) with confidence intervals, effect sizes, degrees of freedom and *P* value noted<br>*Give P values as exact values whenever suitable.*
☒	☐	For Bayesian analysis, information on the choice of priors and Markov chain Monte Carlo settings
☐	☒	For hierarchical and complex designs, identification of the appropriate level for tests and full reporting of outcomes
☐	☒	Estimates of effect sizes (e.g. Cohen's *d*, Pearson's *r*), indicating how they were calculated

Our web collection on statistics for biologists contains articles on many of the points above.

Software and code

Policy information about availability of computer code

Data collection	Getdata (version 2.20) was used to extract data for figures.
Data analysis	Open source code R 4.2.1 is used for statistical analyses （R core team)

For manuscripts utilizing custom algorithms or software that are central to the research but not yet described in published literature, software must be made available to editors and reviewers. We strongly encourage code deposition in a community repository (e.g. GitHub). See the Nature Portfolio guidelines for submitting code & software for further information.

Data

Policy information about availability of data

All manuscripts must include a data availability statement. This statement should provide the following information, where applicable:

- Accession codes, unique identifiers, or web links for publicly available datasets
- A description of any restrictions on data availability
- For clinical datasets or third party data, please ensure that the statement adheres to our policy

The data and code that support the findings of this study are available in zenodo at 10.5281/zenodo.10374039

Research involving human participants, their data, or biological material

Policy information about studies with human participants or human data. See also policy information about sex, gender (identity/presentation), and sexual orientation and race, ethnicity and racism.

Reporting on sex and gender	Not applied.
Reporting on race, ethnicity, or other socially relevant groupings	Not applied.
Population characteristics	Not applied.
Recruitment	Not applied.
Ethics oversight	Not applied.

Note that full information on the approval of the study protocol must also be provided in the manuscript.

Field-specific reporting

Please select the one below that is the best fit for your research. If you are not sure, read the appropriate sections before making your selection.

☐ Life sciences ☐ Behavioural & social sciences ☒ Ecological, evolutionary & environmental sciences

For a reference copy of the document with all sections, see nature.com/documents/nr-reporting-summary-flat.pdf

Ecological, evolutionary & environmental sciences study design

All studies must disclose on these points even when the disclosure is negative.

Study description	We conducted a global meta-analysis of particulate organic carbon (POC) and mineral-associated organic carbon (MAOC) based on 118 fertilization experiments. This analysis also examined the relationship between initial soil organic carbon and effect size in global cultivated uplands. Additionally, we analyzed four long-term (greater than 10 years) nitrogen fertilization experiments to further evaluate how nitrogen addition affects POC and MAOC in relation to initial soil organic carbon, and to elucidate the underlying mechanisms of carbon pool formation.
Research sample	For meta-analysis, we conducted an extensive search for studies of N fertilization and POC/MAOC published between 1999 and 2024 through Web of Science, Google Scholar and the China Knowledge Resource Integrated Database (http://www.cnki.net/). This yielded a dataset of 609 observations of POC/MAOC from 118 field studies. We investigated whether the effect of nitrogen input on particulate organic carbon (POC) and mineral-associated organic carbon (MAOC) depends on the initial soil organic carbon content. Additionally, we investigated the underlying mechanisms by which nitrogen fertilizer affects the accumulation of carbon pool components through four field experiments.
Sampling strategy	For meta-analysis, the following keywords: (“nitrogen”) AND ("soil carbon fraction" OR "soil mineral-associated organic carbon" OR "soil mineral-associated organic matter" OR "MAOC" OR "MOC" OR "MAOM" OR "MOM" OR "particulate organic matter" OR "particulate organic carbon" OR "POC" or POM") AND ("farmland" OR "cropland" OR "agriculture"). For in inclusion in the database, published research must have satisfied the following criteria: (1) an N fertilization experiment was conducted in the field and included N-addition and control treatments within the same ecosystem under the same environmental conditions; (2) no other organic manure was applied in all treatments during the experiments; (3) C pools and at least one related variable were measured, and the mean, standard deviation or standard error, and sample size were reported for all treatments; (4) the experiment was conducted in cultivated upland.<br>For field sampling, in September 2020 when crop harvested, four soil cores (5 cm diameter and 0-20 cm depth) were collected from each plot in four sites.
Data collection	Ranran zhou conducted the literature search, screened all literature. Jun Ling calculated effect size of POC/MAOC, and coded the information from the studies. Jun Ling also collected field soil samples.
Timing and spatial scale	For meta-analysis, the searching for publication and data collection continuously proceeded from Jan, 2023 to July, 2023. The timing scale of the observations in original studies from 1999 to 2023. For the field experiments, soil samples were collected in September 2020 across four sites.
Data exclusions	No data were excluded from the analyses.
Reproducibility	Our study is an integrated study with two comprehensive, global meta-analyses based on published reference data and four field experiments. Our results can be reproduced when following the described methods and data.
Randomization	Soil samples were randomly collected from field station without a priori expectations that the sampling would influence the analysis.
Blinding	Not applicable. There were no human or animal research participants involved in our study.

Did the study involve field work? [x] Yes [ ] No

Field work, collection and transport

Field conditions	The four sites were located in Siping (SP) Experimental Station, Jilin Province in Northeast China (43°20′N, 124°20′E); Quzhou Experimental Station (QZ), Hebei Province in the North China Plain (36°53′N, 115°12′E); Changwu Experimental Station (CW), Shaanxi Province in Northwest China (35°28′N, 107°88′E); and Yaan Experimental Station (YA), Sichuan Province in Southwest China (29°59′N, 103°14′E). At Siping, the soil texture is silt clay with 16.3 g kg-1 SOC, 1.69 g kg-1 TN and pH 6.16. The area had an average air temperature of 6.81 °C and mean annual precipitation of 551 mm. In Quzhou experimental station, the soil texture is clay loam with 9.49 g kg-1 SOC, 0.87 g kg-1 TN and pH 8.5. The area had an average air temperature of 13.2 °C and mean annual precipitation of 516 mm. In Changwu experimental station, the soil texture is silt loam with 9.51 g kg-1 SOC, 0.95 g kg-1 TN and pH, 8.4. The area had an average air temperature of 9.2 °C and mean annual precipitation of 595 mm. In Yaan experimental station, the soil texture is clay loam with 17.4 g kg-1 SOC, 1.29 g kg-1 TN and pH, 6.3. The area had an average air temperature of 17.1 °C and mean annual precipitation of 1635 mm.
Location	The four sites were located in Siping (SP) Experimental Station, Jilin Province in Northeast China (43°20′N, 124°20′E); Quzhou Experimental Station (QZ), Hebei Province in the North China Plain (36°53′N, 115°12′E); Changwu Experimental Station (CW), Shaanxi Province in Northwest China (35°28′N, 107°88′E); and Yaan Experimental Station (YA), Sichuan Province in Southwest China (29°59′N, 103°14′E).
Access & import/export	Project and class site use requests were completed for our study.
Disturbance	No disturbance was caused by the study.

Reporting for specific materials, systems and methods

We require information from authors about some types of materials, experimental systems and methods used in many studies. Here, indicate whether each material, system or method listed is relevant to your study. If you are not sure if a list item applies to your research, read the appropriate section before selecting a response.

Materials & experimental systems

n/a	Involved in the study
[x]	[ ] Antibodies
[x]	[ ] Eukaryotic cell lines
[x]	[ ] Palaeontology and archaeology
[x]	[ ] Animals and other organisms
[x]	[ ] Clinical data
[x]	[ ] Dual use research of concern
[x]	[ ] Plants

Methods

n/a	Involved in the study
[x]	[ ] ChIP-seq
[x]	[ ] Flow cytometry
[x]	[ ] MRI-based neuroimaging

Plants

Seed stocks	Not applied.
Novel plant genotypes	Not applied.
Authentication	Not applied.
