# Supplementary material for: Soil organic carbon thresholds control fertilizer effects on carbon accrual in croplands worldwide
Source: Nat Commun. 2025 Mar 27;16:3009. doi: 10.1038/s41467-025-57981-6 (PMC11950326; doi:10.1038/s41467-025-57981-6)
Supplement: Supplementary file 2 — Peer Review file [file 41467_2025_57981_MOESM2_ESM.pdf]

nature portfolio

Peer Review File

Soil organic carbon thresholds control fertilizer effects on carbon accrual in croplands worldwide

Corresponding Author: Professor Jing Tian

This file contains all reviewer reports in order by version, followed by all author rebuttals in order by version.

Version 0:

Reviewer comments:

Reviewer #1

(Remarks to the Author)
Ling and the co-authors investigated the effect of N addition on POC and MAOC in cropland through meta-analysis and field experiments. They found that initial SOC threshold of 1.5% controls the responses of POC and MAOC to N fertilization. They also explained the mechanisms of the differential responses of POC and MAOC to N fertilizations under SOC-poor and -rich soils. The findings were interesting and meaningful. However, at present form, the manuscript has many obvious problems. Before it could be considered for publication, the manuscript needs a thorough revision.
Major concerns:
1, The results showed that N fertilization significantly increased POC content both before and after the threshold (initial SOC 1.5%) (Fig 1d). In other words, the response of POC to N fertilization may be not depend on the initial SOC.
2, The belowground plant biomass (i.e., fine root biomass) is more important than aboveground plant biomass in regulating the dynamics of soil aggregates or SOC. However, the author only used aboveground plant biomass to interpret the response of POC or MAOC to N fertilization. It is inadequate and inappropriate.
Minor concerns:
1, the Keywords was unrepresentative and inappropriate, and I don’t know what the manuscript mainly want to present after reading these keywords
2, Line 85-86, global meta-analysis first and then field experiments. Exchange the order please.
3, Line 89-90, the hypothesis was poor, and it seems like the authors already knew the results before they conducted the experiments. We cannot find any information about the effect of N fertilization on soil aggregates in Introduction section.
4, Line 128, How did you conduct threshold analysis, please clarify.
5, Line 142-143, Please give the full name of these abbreviations.
6, Line 277-278. There were several global meta-analyses had already investigated the response of MAOC to N addition except the citation in this manuscript. For example, Tang et al. (2023) (Global Change Biology, DOI: 10.1111/gcb.16588) and Wu et al. (2023) (Soil and Tillage Research, DOI:10.1016/j.still.2023.105770), both investigated the effect of N addition on MAOC on a global scale.
7, Line 351, How about the land managements in these different sites? Whether they were same or not?
8, Line 485, What was the pretreatment before soil aggregates size fractions? Please clarify.
9, Figure 1a, It looks strange for the distribution of the study sites. The studies in North America and Europe were lacking. However, a lot of studies were conducted in India. Please check the data.

Reviewer #2

(Remarks to the Author)
This is an impressive manuscript with a wealth of data, used to generate a theoretical framework of SOC responses to N fertilization. The topic is interesting, important and the way the story is build up would generally suit the journal. The main finding of an SOC threshold that determines weather POC or MAOC is preferentially formed due to mineral fertilization is generally interesting. However, I fear that this might be too simple to be true. First of all, the literature thresholds (Fig1) seam somewhat arbitrary and I cannot find any explanation on how they were determined (statistically). Furthermore, in the whole paper, the influence of texture on POM and MAOM is totally missing, although texture is highly important for the proportion of those OM pools and also the formation efficiency of MAOM. It was the second most important variable in the literature dataset, and also in the two long-term experiments a clay loam and a silt loam are compared (more MAOM formation in the

clay loam, which is expected but never mentioned as a potential influence). Texture and SOC are generally coupled. Nevertheless, the authors never mention any potential texture influence, but define a texture-independent SOC threshold, which appears highly unlikely. Not even a specific land-use type is really mentioned (although I believe it is about agricultural soils only), or the C:N ratio of the SOC itself, which could also play a role in this context. So, in essence the authors assume that sandy forest soils with a lot of POM and a wide C:N ratio, would follow the same pattern (regarding the threshold) as fine-textured agricultural soils, which is hard to believe because 1.5% SOC is a completely different thing in those two situations. In this context, and also in general, the predictive modelling and mapping of POC and MAOC increases across the globe (here it is farmland) is weak and misleading. I do not think that there is an increase in MAOC and POC under current fertilization practices in global agricultural soils. Authors write about restoring SOC in degraded agricultural soils by N fertilization. In many parts of the world (industrialized agriculture), there is already to much N in the system, so the overall potential to increase N inputs is low. Moreover, we can even observe losses of SOC in fertilized agricultural soils all over the world, so fertilization alone is not the answer. Therefore, I do not think that showing the maps makes much sense. Overall, although I see the potential of that paper and dataset to advance our understanding on SOC responses to mineral N fertilization, I am not convinced yet and recommend that major revisions are necessary.

Specific comments:
l.50-52: I don’t agree with that statement. Not everywhere in the world, an increase in fertilization is possible or helpful to increase crop yields. In many parts, soils are already overfertilized.
l.79-81: This sentence is not very logical to me. Where is the connection between mineral properties and initial SOC?
l.91: . and is wrong.
l.93: MAOC instead of MOAC
l.181: What did you mean by N fertilization aggregated C limitation?
l. 261: F:B ratio: Why is it increasing with N fertilization? I expected the opposite.
l.287: recent advances of which kind?
l.323-325: This contradicts state-of-the-art literature. Others either find a relatively stable ratio of POC and MAOC across a wide range of C contents (Begill, Matus) or an increasing proportion of POC with increasing SOC (Cotrufo).
L363-366: How large is the actual difference in texture between the two experiments? That is important, because this could also explain the difference in SOC, as well as the dynamic of MAOC and POC.
l.520: this whole section is not explained well enough. How is the globa mapping done, which kind of covariate maps are in the background, and where did they come from?

Reviewer #3

(Remarks to the Author)
Dear Authors,
I have enjoyed reviewing your manuscript entitled "Soil organic carbon thresholds control fertilizer effects on carbon accrual in croplands worldwide." Your research presents valuable insights into unraveling the N fertilization effects on soil organic carbon dynamics, which has significant implications for environmental and agricultural management.
Overall, I found your paper well-written, scientifically sound, and impactful. The data and analyses you present provide a strong foundation for your conclusions, and your discussion of global implications is well-argued. However, I have a few comments and questions that could further strengthen the manuscript:
- L91 “. and”
- L172-174. N fertilizer effect on lignin phenols was not significant in C-poor soil (Figure 3a; Supplementary Figure 8). I see the trend, but could you soften the statement or provide the p-value?
- L187 “…the mean weight diameter (MWD) of aggregates”
- L261 F/B was not defined. (chose between F:B or F/B to refer to the ratios)
- L306. I'm confused with what you refer to with the recalcitrant and labile pools. Aliphaticity and recalcitrance are indexes but do not represent SOC pools. The organic compounds interacting with minerals in the MAOM pools are not necessarily recalcitrant; they could be labile compounds that do not decompose because of mineral protection. Therefore, I'm not sure that the accumulation of SOC is attributed to the "recalcitrant C pool." Aliphaticity and recalcitrance increased in C-rich soil with N addition, but we don't know if these compounds are in POC or MAOC. N could produce a quality change in POC that does not necessarily explain increases in MAOC or SOC.
- L305. MAOC could also have plant-derived compounds, not just necromass.
- L307. Belowground biomass was not measured, so assessing it is impossible. However, roots and rhizodeposition likely play a role in MAOC formation, not just AGB. Nitrogen fertilization positively impacted AGB, and it is likely that it also increased root biomass (bigger plants, more roots too). It would be good to mention the potential role of roots in your discussion.
- L821 “…Global map of particulate organic carbon (POC) changes”; “Global map of mineral-associated organic carbon (MAOC) changes”
- Figure 1. I would flip the color for C-poor and C-rich soils. This is more personal and unnecessary, but for me, it is more intuitive that the green color is associated with higher SOC. In panels d and e, the RR of reference (no change) should be 1 – not zero – according to its definition in methods. In panels a and b, the linear fitted models at two sides of the threshold ignore the continuum nature of the initial SOC. Given that there are different models, an abrupt change in the estimation occurs from one side to the other side of the threshold. Therefore, I think it is not a good way to assess differences between C-poor and C-rich soils. In addition, using bootstrapping to assess the slopes of the model assumes independence between groups. However, some data in these groups could come from the same study, and this independence assumption is

violated. The generalized additive model is more appropriate. (The caption does not explain the difference between solid and dashed blue lines).
- Figure 2 (b,c) This figure has too much information, and the analysis shown in panels b and c is not intuitive. I suggest trying another way to highlight this correlation's main findings and may leave these panels for the supplementary material. In panel c, the meaning of color dots is not described, and the underscore in variable names should be removed.
- Figure 4. I would explore a different legend scale to see clearer the spatial patterns.
- Is it possible to know which types of crops are represented in the meta-analysis? Are they all annual crops?

Version 1:

Reviewer comments:

Reviewer #1

(Remarks to the Author)
The authors did a good job, and they addressed all of my concerns, I have no more comments.

Reviewer #2

(Remarks to the Author)
The authors took a great effort to revise the manuscript, which is now much improved. The analytical and statistical part of that manuscript is very strong, my remaining points are mostly related to the text. I believe that it can be ready for publication after some moderate revisions:

l.44-46: I have the feeling that it should be toned down a bit. On average, POC and MAOC are promoted in all cases. There just seams to be a higher tendency of MAOC vs. POC formation in high C soils. This should be better reflected here.

l.70: increases of plant C inputs

l.76-85: I have the feeling that the EST part and particularly the difference between C-poor and C-rich soils still needs some better explanation here. How and why does the stoichiometric imbalance (C limitation) occur in a soil with 1.4% C and not in a soil with 1.6% C. When it is so driven by stoichiometry, then it should not only be the amount of C in the soil, but e.g. also the amount of N that is added to the soil, right? It should be crystal-clear, why such a sharp threshold value is mechanistically justified.

l.88-89: In the last round I asked for the inclusion of texture, but this is not the way it should be done. This sentence makes no sense.

l.298; maybe call it “a particularly positive effect” to clarify that both fractions in both soils were positively affected by N fertilization (at least in the literature dataset).

l.320-321: Don’t make it sound too general, here you are writing about your experiments.

l.255: long-term filed networking experiments? What does that mean?

l.301: This indicates a shift… Grammar in this sentence is not correct.

l.337-340: It could also be mentioned, that C sequestration via N fertilization is also not a climate mitigation measure, because N fixation requires a lot of energy and N2O emissions are also strongly increased. The overall GHG budget of a fertilized soil is still less positive than that of an unfertilized soil. This should be briefly mentioned here as well.

l.383: Again, the term predominantly is somewhat to strong. In the large literature dataset, the difference is not so large, so I would rather call it “a more pronounced accumulation of POC…” or similar.

Reviewer #3

(Remarks to the Author)
The authors responded satisfactorily to my reviews and provided an improved version. My recommendation is to accept the manuscript.

Version 2:

Reviewer comments:

Reviewer #2

(Remarks to the Author)

Thanks for doing the requested changes. The latest version of the manuscript can be accepted as is.

Open Access This Peer Review File is licensed under a Creative Commons Attribution 4.0 International License, which permits use, sharing, adaptation, distribution and reproduction in any medium or format, as long as you give appropriate credit to the original author(s) and the source, provide a link to the Creative Commons license, and indicate if changes were made.
In cases where reviewers are anonymous, credit should be given to 'Anonymous Referee' and the source.
The images or other third party material in this Peer Review File are included in the article's Creative Commons license, unless indicated otherwise in a credit line to the material. If material is not included in the article's Creative Commons license and your intended use is not permitted by statutory regulation or exceeds the permitted use, you will need to obtain permission directly from the copyright holder.
To view a copy of this license, visit https://creativecommons.org/licenses/by/4.0/

Response to Reviewers' comments

A. Response to Reviewer #1:

[A1] Ling and the co-authors investigated the effect of N addition on POC and MAOC in cropland through meta-analysis and field experiments. They found that initial SOC threshold of 1.5% controls the responses of POC and MAOC to N fertilization. They also explained the mechanisms of the differential responses of POC and MAOC to N fertilizations under SOC-poor and -rich soils. The findings were interesting and meaningful. However, at present form, the manuscript has many obvious problems. Before it could be considered for publication, the manuscript needs a thorough revision.

Response: We appreciate the time and effort taken by the Reviewer to provide supportive comments. We hope that we have satisfied the queries raised by the Reviewer that enabled us to revise and improve our manuscript substantially. Please see our detailed responses to your comments listed below.

[A2] The results showed that N fertilization significantly increased POC content both before and after the threshold (initial SOC 1.5%) (Fig 1d). In other words, the response of POC to N fertilization may be not dependent on the initial SOC.

Response: We appreciate the Reviewer's comment. We agree with the Reviewer that POC increased both before and after the threshold. However, we found that the response of POC to N fertilizer showed a significant change in the linear regression slope with initial SOC content both before and after threshold ($P < 0.001$; Fig. 1b). Heterogeneity analysis further revealed that the effect of N fertilizer on POC was greater before, compared to after, the threshold ($Q_M = 19.5$, $P < 0.001$; Fig. 1d). We have made the following changes to the Results based on Reviewer's comment:

Lines 145–150: "This notion is further supported by heterogeneity analysis, demonstrating clear differences in the POC and MAOC responses before and after reaching the SOC threshold under N addition ($Q_M = 19.5$, $P < 0.001$ for POC; $Q_M = 9.24$, $P < 0.001$ for MAOC; Figs. 1d, e). Note that the POC increase under N fertilization was more important before the threshold than afterward, whereas the MAOC response followed the opposite trend."

Lines 646–648: "We also used the Q-statistic to test the heterogeneity of the N

effect sizes on POC and MAOC between before and after the SOC threshold. Significant Q_M values (between-group heterogeneity) indicated that the RR differed between groups ($P < 0.05$)."

[A3] The belowground plant biomass (i.e., fine root biomass) is more important than aboveground plant biomass in regulating the dynamics of soil aggregates or SOC. However, the author only used aboveground plant biomass to interpret the response of POC or MAOC to N fertilization. It is inadequate and inappropriate.

Response: We thank the Reviewer for their advice. We agree with the Reviewer that root biomass is more important than aboveground plant biomass in regulating the dynamics of soil aggregates or SOC. Therefore, we have replaced the date for aboveground biomass for root biomass in the revised MS. We have made corresponding revisions to the Results and Discussion sections, including updating Fig. 3. Our results showed that the inclusion of root biomass did not change our primary conclusions: the C source and stability remain key factors in determining POC and MAOC accumulation. (Note that our previous results showed that the root biomass and aboveground biomass were highly correlated across all four sites (Quzhou: $R^2 = 0.958$, $P < 0.001$; Changwu: $R^2 = 0.974$, $P < 0.001$; Siping: $R^2 = 0.967$, $P < 0.001$; Yaan: $R^2 = 0.962$, $P < 0.001$). Due to the strong collinearity between root biomass and aboveground biomass, as indicated by their high correlation and variance inflation factor, we included only aboveground biomass in the partial correlation analysis. Therefore, we had presented the aboveground biomass data in the previous manuscript only.)

Lines 183-185: "As compared to N0, N fertilizer increased root biomass in C-poor (~148%) and C-rich (~115%) soils, respectively ($P < 0.001$; Fig. 3a and Supplementary Fig. 7)."

Lines 211–224: "Partial correlation analysis showed that, in C-poor soils, despite the significant correlations between POC and the four types of factors (i.e., SOC origin, microbial transformation, stability and structure; zero-order in Fig. 3b), the correlation coefficients of POC and Mg_{exe} decreased by 203% after controlling for the SOC origin (Fig. 3b). By contrast, root biomass and MWD were always closely related to the POC in C-poor soils ($P < 0.001$; Fig. 3d), even after controlling for the other three or two types of factors. Specifically,

increasing root biomass and MWD was coupled with increasing POC ($R^2 = 0.68$, $P < 0.001$; $R^2 = 0.45$, $P < 0.001$) in C-poor soils (Fig. 3d). In C-rich soils, after controlling SOC stability, the correlation coefficients between MAOC and C origin (characterized by plant C input and biomarkers) became insignificant (Fig. 3c). Moreover, the root biomass and Fe_p + Al_p consistently increased with the MAOC (Fig. 3e; $R^2 = 0.58$, $P < 0.001$; $R^2 = 0.65$, $P < 0.001$). Accordingly, plant C input was the single control in all soils, exerting strong positive effects on POC and MAOC. Physical protection and mineral protection were strongly associated with POC accumulation in C-poor soils and MAOC accumulation in C-rich soils, respectively (Fig. 3)."

[A4] the Keywords was unrepresentative and inappropriate, and I don't know what the manuscript mainly wants to present after reading these keywords.
Response: We thank the Reviewer for raising this issue. We have revised the keywords to better reflect the focus of our research.

Lines 51–52: "Keywords: Particulate organic carbon; Mineral-associated organic carbon; Nitrogen fertilizer; Agriculture; Initial SOC content"

[A5] Line 85-86, global meta-analysis first and then field experiments. Exchange the order please.
Response: We thank the Reviewer for raising this issue. We have revised this sentence to make it clearer, it now reads:

Line 96–98: "Herein, we combined a global meta-analysis with new data from decades-long continental-scale N fertilization field experiments in four sites across China (Fig. 1a and Supplementary Fig. 1) to investigate how initial SOC content may interact with N fertilization to influence the SOC accrual of global cropland soils."

[A6] Line 89-90, the hypothesis was poor, and it seems like the authors already knew the results before they conducted the experiments. We cannot find any information about the effect of N fertilization on soil aggregates in Introduction section.
Response: We thank the Reviewer for drawing attention to our hypotheses. We have added a statement about the effect of N fertilization on soil aggregates supported by a recent literature citation (Lu et al., 2021; Fohrafellner et al., 2024)

to the Introduction section. We also revised Hypothesis 1 to enhance its logical flow and scientific rigor.

Lines 90–93: "Beyond influencing mineral properties, N fertilization may also strengthen macroaggregate formation by stimulating root and mycorrhizal growth, which, together with plant-derived C, contribute to POC pool and its stabilization as intra-aggregate POC [28,29]."

Lines 99–101: "N fertilization promotes plant productivity and macroaggregate formation, and thereby increases POC accumulation and stabilization;"

Lu, X., Hou, E., Guo, J., Gilliam, F. S., Li, J., Tang, S., & Kuang, Y. (2021). Nitrogen addition stimulates soil aggregation and enhances carbon storage in terrestrial ecosystems of China: A meta-analysis. Global Change Biology, 27(12), 2780–2792. https://doi.org/10.1111/gcb.15604

Fohrafellner, J., Keiblinger, K. M., Zechmeister-Boltenstern, S., Murugan, R., Spiegel, H., & Valkama, E. (2024). Cover crops affect pool specific soil organic carbon in cropland – A meta-analysis. European Journal of Soil Science, 75(2), e13472. https://doi.org/10.1111/ejss.13472

[A7] Line 128, How did you conduct threshold analysis, please clarify.

Response: We apologize for the lack of clarity in our original method description. We assessed whether a linear or nonlinear fit was more appropriate for our data by using the Akaike Information Criterion (AIC). When the nonlinear fit proved superior, we further evaluated six nonlinear models and selected the one that best fit the data (Supplementary Table 1). We have now revised and clarified the Method section, providing detailed steps for our threshold analysis, and we hope this revision will resolve any confusion.

Lines 626–648: "We first fitted linear and non-linear (quadratic and general additive models [GAM]) regressions to the relationships between the effect sizes of POC/MAOC and initial SOC content, and used the Akaike information criterion (AIC) to decide which model was the best fit in each case [70,71]. Thresholds could only be defined when non-linear regressions best fit the data. Then, we evaluated six different nonlinear models to select the one that best aligned with our data (Supplementary Table 1), and which each differed from each other by the functions fitted either side of the estimated breakpoint [72]. The nonlinear models evaluated were: general additive models (GAM), step (two intercept models, for which the differences in intercept were tested at the

breakpoint), segmented (two linear models in which the slope is changed at breakpoint), stegmented (two linear models in which both the slope and the intercept are changed at the breakpoint), hinge model 12 (one linear model is fitted for the left part of the breakpoint and a second degree polynomial is fitted for the right part), and hinge model 22 (two different second degree polynomial models are fitted at both sides of the breakpoint). The model exhibiting the lowest AIC and Bayesian information criterion (BIC) was considered the best model. Detailed AIC and BIC values for each regression model are provided in Supplementary Table 1. We defined the breakpoint as the initial SOC threshold.

To further test whether the thresholds identified significantly affected the slope of the fitted regressions, we bootstrapped linear regressions either side of each threshold for each variable. We then extracted the slope and the predicted value of the variable evaluated before and after the threshold and compared them using a Mann-Whitney U test following the methods in ref. [71] and [70]. We also used the Q-statistic to test the heterogeneity of the N effect sizes on POC and MAOC between before and after the SOC threshold. Significant Q_M values (between-group heterogeneity) indicated that the RR differed between groups ($P < 0.05$)."

Supplementary Table 1. Results of the model selection procedure used to evaluate the response between effect sizes of POC/MAOC and initial SOC.

Index	model	AICs	ΔAICs	BICs	ΔBICs
POC	**Stegmented**	**491**	**0.00**	**519**	**0.00**
	M22	522	31.1	554	35.3
	Step	545	53.6	564	45.2
	M12	558	66.8	585	66.8
	Segmented	564	72.4	587	68.2
	GAM	589	97.7	602	83.1
MAOC	**Stegmented**	**-100**	**0.00**	**-82.3**	**0.00**
	Step	-96.3	3.88	-80.1	2.13
	M22	-92.9	7.30	-76.7	5.51
	M12	-92.6	7.62	-75.5	6.80
	Segmented	-92.5	7.68	-72.5	9.74
	GAM	-85.8	14.4	-69.8	12.4

Bold fonts indicate the best model. The best fitting models showed the lowest AIC and BIC values within a ΔAIC and ΔBIC < 2. POC: particulate organic carbon; MAOC: mineral-associated organic carbon; SOC: soil organic carbon. GAM: generalized additive model; step: two intercept models, with no slope fitted and the intercept is changed at the breakpoint; segmented: two linear models in which the slope is changed at the breakpoint; stegmented: two linear models in which both the slope and the intercept are changed at the breakpoint; M12: hinge model 12, one linear model is fitted for the left part of the breakpoint and a second-degree polynomial is fitted for the right part; M22: hinge model 22, two different second-degree polynomial models are fitted at both sides of the breakpoint. AIC: Akaike information criteria; BIC: Bayesian information criteria.

[A8] Line 142-143, Please give the full name of these abbreviations.

Response: Thank you for pointing this out. We have supplied the full name of these abbreviations.

Lines 155-157: "These experiments were located in four cropland sites with different initial SOC contents: Quzhou (QZ, 9.49 g kg^{-1}), Changwu (CW, 9.51 g kg^{-1}), Siping (SP, 16.3 g kg^{-1}) and Yaan (YA, 17.4 g kg^{-1})."

[A9] Line 277-278. There were several global meta-analyses had already investigated the response of MAOC to N addition except the citation in this manuscript. For example, Tang et al. (2023) (Global Change Biology, DOI: 10.1111/gcb.16588) and Wu et al. (2023) (Soil and Tillage Research, DOI:10.1016/j.still.2023.105770), both investigated the effect of N addition on MAOC on a global scale.

Response: We thank the Reviewer for highlighting the need to update our references and for bringing these useful references to our attention. We read these references very carefully and used the information to update our manuscript. Please see lines 287–288.

[A10] Line 351, How about the land managements in these different sites? Whether they were same or not?

Response: We appreciate the Reviewer's suggestion. We have provided detailed information about the fertilization management. The farm management practices including tillage and straw residue were consistent across the four sites. This information has now been included in the Material and Methods section.

Line 409-410: "The same fertilizer experiments were established in a randomized complete-block design with four replicates at each site."

Lines 415–417: "Fertilizer was applied twice during the growing period: initially as a basal application and subsequently as a top-dressing in all four sites. Maize straw was chopped and incorporated into the soil through tillage to a depth of approximately 20 cm."

[A11] Line 485, What was the pretreatment before soil aggregates size fractions? Please clarify.

Response: Thank you for raising this point. We used the wet-sieving method to evaluate the soil aggregate stability. We have supplemented the pretreatment information in the revised text:

Lines 532–536: "In brief, undisturbed air-dried soil samples were gently crushed to pass an 8-mm sieve mesh. Then, 100 g samples of ~8 mm-sized aggregates were placed in the top sieve of a set containing sieves with 2.0 mm, 0.25 mm, and 0.053 mm mesh sizes that was submerged and oscillated (amplitude ~ 2.5 cm) in deionized water. The soil material was rinsed from each sieve and then oven-dried and weighed."

[A12] Figure 1a, it looks strange for the distribution of the study sites. The studies in North America and Europe were lacking. However, a lot of studies were conducted in India. Please check the data.

Response: We appreciate the Reviewer's comment. We have rechecked the 118 articles in the database, classifying the sites at both national and intercontinental scales (Fig. 1, below). Most data points were from Asia, followed by North America. At the national level, China (n = 26), India (n = 24), and the United States (n = 6) had the highest number of data points. Additionally, when comparing our study with Tang et al. (2023) (Global Change Biology), we found that their sampling sites in North America and Europe were also limited (North America = 10, Europe = 3). It is worth noting that our inclusion criteria differ, which may account for some sample variations. Nonetheless, research in North America and Europe remains relatively scarce, or possibly unpublished.

We further checked studies from India, with specific reference to "Meta-analysis database references" in the supplementary material: 10, 12, 13, 16, 18, 20, 27, 29, 42, 44, 47, 59, 63, 64, 69, 72, 74, 78, 83, 84, 84, 87, 91, 96,

which included a total of 24 studies, accounting for 20.3%.

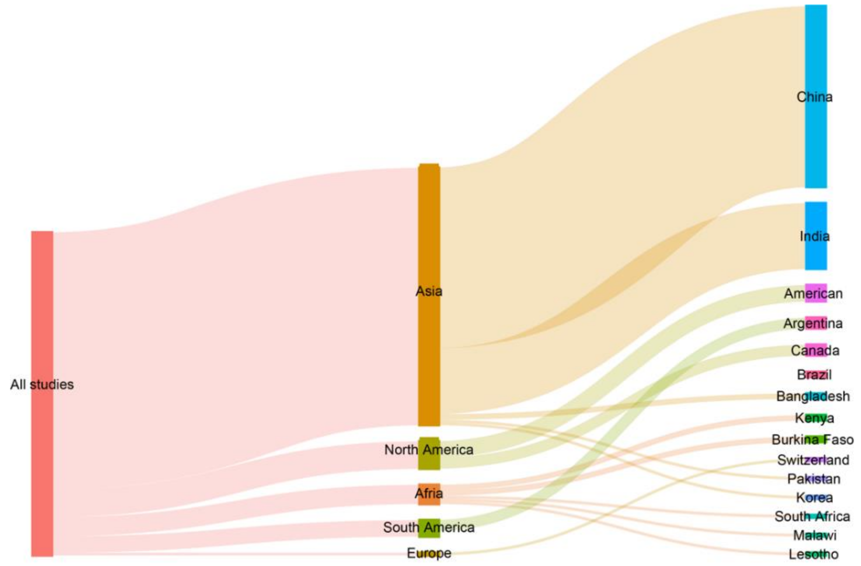

Figure 1. Countries and continents of the studies included in the Meta database.

B. Response to Reviewer #2

[B1] This is an impressive manuscript with a wealth of data, used to generate a theoretical framework of SOC responses to N fertilization. The topic is interesting, important and the way the story is build up would generally suit the journal.

Response: We are grateful to the Reviewer for their positive and supportive comments and for highlighting the strengths and novelty of our study.

[B2] The main finding of an SOC threshold that determines whether POC or MAOC is preferentially formed due to mineral fertilization is generally interesting. However, I fear that this might be too simple to be true. First of all, the literature thresholds (Fig1) seam somewhat arbitrary and I cannot find any explanation on how they were determined (statistically).

Response: We apologize for any lack of clarity in our original method description. We first assessed whether a linear or nonlinear fit was more appropriate for our data by using the Akaike Information Criterion (AIC). When the nonlinear fit proved superior, we evaluated six different nonlinear models and selected the one that best aligned with our data (Supplementary Table 1). We have now revised and clarified the method section, providing detailed steps for our threshold analysis, and we hope this revision will resolve any confusion. We refer the Reviewer to our response *comment A7* above (Lines 626–641).

[B3] Furthermore, in the whole paper, the influence of texture on POM and MAOM is totally missing, although texture is highly important for the proportion of those OM pools and also the formation efficiency of MAOM. It was the second most important variable in the literature dataset, and also in the two long-term experiments a clay loam and a silt loam are compared (more MAOM formation in the clay loam, which is expected but never mentioned as a potential influence).

Response: We thank the reviewer for drawing our attention to soil texture. In this study, among all the key factors examined, SOC emerged as the independent and interactive factor, showing close correlations with other variables (Supplementary Fig. 4). Therefore, we have given SOC special consideration.

We fully agree with the Reviewer that soil texture (represented by silt and clay content in this study) is indeed an important factor influencing the response

of POC and MAOC to N fertilization (Supplementary Fig. 4). Following the Reviewer comments, we further categorized the soil texture data into three groups—sandy, loam, clay-loam, and clay—based on the international standard for soil texture classification (Supplementary Fig. 15). Our meta-analysis showed significant differences in POC content among soils with these texture groups using the between-group heterogeneity test (Supplementary Fig. 15). Note that the increase of POC under N fertilization was more significant in clayey soils than that in clay loamy soils (Q_M = 12.3, P = 0.002). However, this increase of POC in clayey soils was not significantly different from that for in loamy soils (Supplementary Fig. 15). Similarly, the response of MAOC to N fertilization was not influenced by soil texture (Q_M = 2.58, P = 0.274; Fig. R2). We fully acknowledge this and have emphasize it further in the discussion.

Lines 287-291: "Soil texture, alongside aggregate formation, is a key factor influencing C accumulation [39–41]. Our meta-analysis indicated that clay and silt content strongly impact POC and MAOC accumulation (Supplementary Fig. 4). Heterogeneity analysis revealed that the effect size of POC is larger in clayey soils than clay loamy soils (Supplementary Fig. 15), as clay has more reactive sites for C adsorption [42]."

Lines 320-322: "Concurrently, C-rich soils have a higher clay-and-silt content (SP and YA, see methods), which provide more mineral surfaces and reactive sites for C adsorption [42,49], thereby promoting MAOC accumulation."

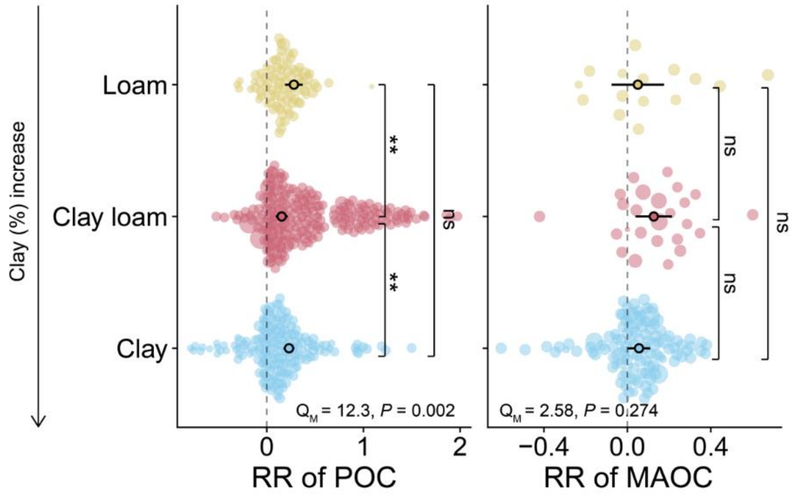

Supplementary Figure 15. Effects of N fertilization on particulate organic carbon (POC) and mineral-associated organic carbon (MAOC) associated with soil

texture.

The between-group heterogeneity (Q_M) statistical test was used to compare the differences in weighted effect sizes among soil texture groups. A significant Q_M value ($P < 0.05$) suggested that the weighted effect sizes of a given variable differed among groups. No samples were classified as sandy, resulting in only three observed soil texture categories. An asterisk means there is a significant difference between the two groups, and ns means no difference. * $P < 0.05$; ** $P < 0.01$; *** $P < 0.001$; ns: no significant.

[B4] Texture and SOC are generally coupled. Nevertheless, the authors never mention any potential texture influence, but define a texture-independent SOC threshold, which appears highly unlikely. Not even a specific land-use type is really mentioned (although I believe it is about agricultural soils only), or the C:N ratio of the SOC itself, which could also play a role in this context. So, in essence the authors assume that sandy forest soils with a lot of POM and a wide C:N ratio, would follow the same pattern (regarding the threshold) as fine-textured agricultural soils, which is hard to believe because 1.5% SOC is a completely different thing in those two situations.

Response: We apologize for the lack of clarity and appreciate the Reviewer's advice. In our study, we use data from agricultural soils only, and we have revised the text to make this clearer (Lines 576–579). We agree with the Reviewer's opinion that "texture affects SOC content." In light of this, we have conducted further analysis on soil texture and have supplemented the relevant information accordingly. We kindly refer the Reviewer to our previous response for additional details (*comment B3*).

It is important to note that the sandy soils were not represented in the studies included in the meta-analysis (Fig. 2, below). Sandy soils are generally less suitable for cropping due to their high hydraulic conductivity, low cation exchange capacity and poor nutrient storage and release, which lead to low water retention, low organic carbon, and making frequent fertilization and irrigation necessary to sustain productivity (Huang & Hartemink, 2020).

Furthermore, we analyzed the soil C:N from the 118 articles included in the meta-analysis. The results showed that C:N in dryland farmland soils were relatively narrow, mostly around 10 (Fig. 3, below), aligning with our understanding of soil texture mentioned above.

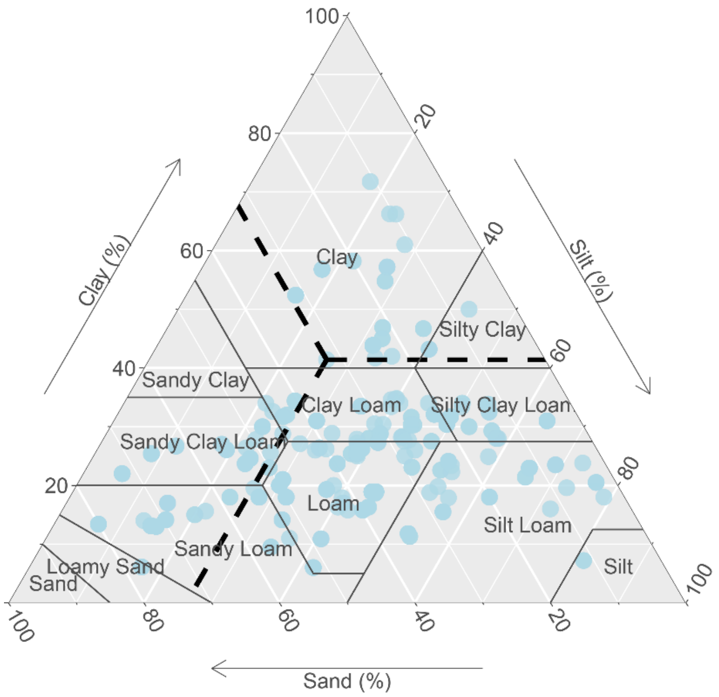

Figure 2. Soil texture classification of the 118 studies included in the meta-analysis.

Each dot represents a single study.

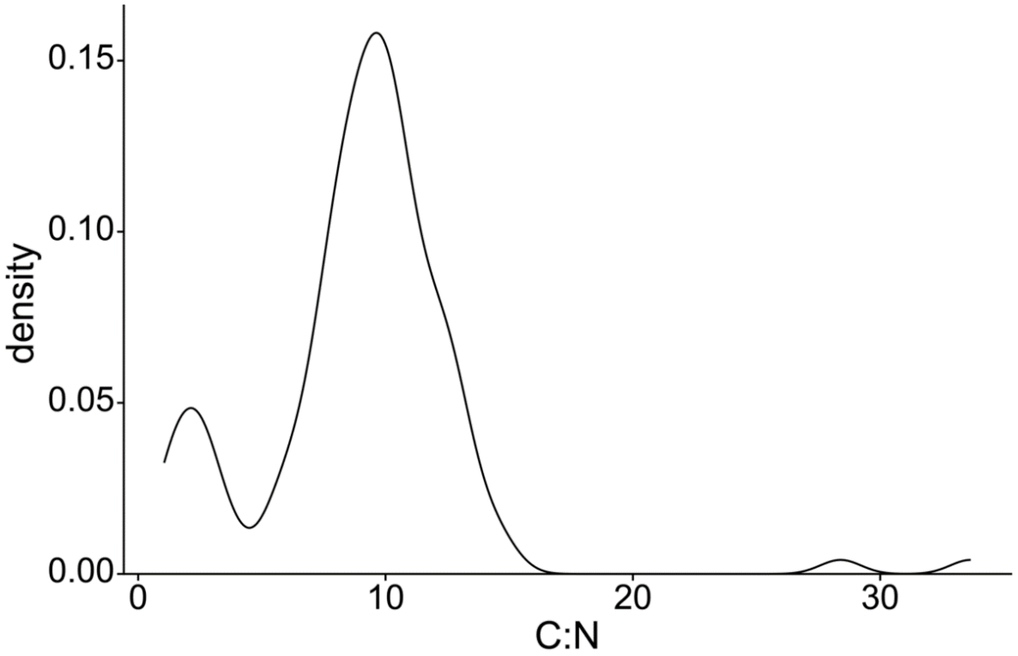

Figure 3. The ratio of SOC to TN (C:N) of the 118 studies included in the meta-analysis.

Huang, J., & Hartemink, A. E. (2020). Soil and environmental issues in sandy soils. *Earth-Science Reviews*, *208*, 103295. https://doi.org/10.1016/j.earscirev.2020.103295

[B5] In this context, and also in general, the predictive modelling and mapping

of POC and MAOC increases across the globe (here it is farmland) is weak and misleading. I do not think that there is an increase in MAOC and POC under current fertilization practices in global agricultural soils. Authors write about restoring SOC in degraded agricultural soils by N fertilization. In many parts of the world (industrialized agriculture), there is already too much N in the system, so the overall potential to increase N inputs is low. Moreover, we can even observe losses of SOC in fertilized agricultural soils all over the world, so fertilization alone is not the answer. Therefore, I do not think that showing the maps makes much sense.

Response: We thank the Reviewer for raising this important point and apologize for our lack of clarity. Our goal was not to advocate for increased fertilization rates, as such practices frequently leads to environmental consequences, including elevated greenhouse gas emissions, groundwater pollution, and biodiversity loss. This concern has been emphasized in lines 338–340. Instead, our aim was to explore the relationship between current fertilization levels and soil C dynamics (lines 330–333). Additionally, we agree that in many over-fertilized regions, the potential for further increasing soil C is limited, as noted in lines 352–354.

Many countries, especially developing counties, have applied the Tier-1 approach from the Intergovernmental Panel on Climate change (IPCC) for assessing soil carbon change due to management practices. While straightforward to implement, the simplified Tier-1method lacks the granularity needed to capture spatial in soil C changes. In addition, the complexity of C pool fractions results in vastly different carbon benefits arising from accumulation in various C pools, a topic that remains underexplored in the IPCC framework. In our study, we compiled the most comprehensive dataset to date on the effects of N fertilization on POC and MAOC in dryland croplands. Utilizing globally sourced covariates, we assessed and mapped the global spatial distribution of POC and MAOC changes induced by fertilization by applying multiple machine learning methods (Material and methods; Lines 595–612; Bergen et al., 2019).

There is indeed a common and logical criticism of regarding the direction and extent of the effect of N fertilizer on organic C accumulation. Many experiments and meta-analysis have documented positive (Han et al., 2016; Li

et al., 2017), neutral (Poffenbarger et al., 2017), and negative (Khan et al., 2007) effects of N addition on soil C accumulation. Similar to the findings of our meta-analysis, SOC exhibited varied responses to N fertilization (Fig. 4, below). While most responses were either neutral or positive, the overall effect was positive. we also agree with the Reviewer's perspective that N fertilization should be managed within a reasonable range to achieve both food security and the restoration or maintenance of C levels. This hypothesis is further supported by a century of SOC data reported here in for the Morrow Plots, the world's oldest continuous maize experimental site (Khan et al., 2007). However, it is undeniable that insufficient N fertilization in underdeveloped regions, such as Africa, restricts crop growth and plant C input to soil, leading to smaller SOC accumulation rates—consistent with our global forecast results.

Our global estimates indicate that N fertilizer application has resulted in a 26.3% increase in POC (Fig. 4a). Compared with no fertilization, the yield-boosting effect of N fertilization is undeniable (Martre et al., 2024; Mueller et al., 2012), leading to greater plant C input and further POC accumulation. Our global meta-analysis indicates that N fertilization increases MAOC by only 8.3% on average compared to no fertilization (Fig. 4b). We integrated findings from the few available meta-analyses and found that N fertilizer generally promotes the sequestration of MAOC in farmland soils (6.35%, 4.56%, 13.1%, 4.68%) (Rocci et al., 2021; Tang et al., 2023; Wu et al., 2023; Xu et al., 2024). These results are comparable to our globally extrapolated estimate (~8%; Fig. 4b). In addition, national-scale data from China confirm that the increase in organic carbon storage in farmland from 1980 to 2010 was primarily driven by greater crop biomass production due to N fertilization and the subsequent incorporation of straw into the soil (Zhao et al., 2018), despite China's reputation for excessive fertilizer use.

Overall, we believe that the global extrapolation results can offer valuable guidance for scientific fertilization strategies (as we mentioned in the Discussion Lines 335–337, 368–370), particularly in the contexts of climate change and food security. However, we recognize the importance of not over-interpreting the prediction results. To address this, we have explicitly discussed the limitations of the global prediction outcomes in the Discussion section (Lines 352–370), ensuring a balanced and cautious interpretation of our findings.

Based on this justification, we hope that the Reviewer will now agree that it is valuable to retain the mapping.

Lines 337–340: "Several existing meta-analysis studies have also shown that fertilization slightly promoted MAOC accumulation [9,12,43,52]. However, this strategy should be implemented with caution to mitigate potential global risks to the economy, environment, and public health [53]."

Lines 352–370: "Although fertilization has been observed to increase C pools (Fig. 4), caution is necessary when assessing its future potential, particularly in areas with overfertilization and high fertility, where the outcomes are often suboptimal [51].While our analysis has enabled the estimation of N fertilization-induced SOC pools changes and climate mitigation potentials, it is important to acknowledge its limitations. First, we must emphasize that the correlations do not imply causation (Figs. 1a, b). In this context, conducting extensive field experiments with N fertilizer gradients, along with detailed plot-level data (for example, plant and soil conditions), and advanced analytical techniques (e.g., structural equation modeling) could further substantiate causal relationships, a nuance that remains a limitation in our current approach. Second, a portion of the variability in C pools changes remains unexplained (Supplementary Table5), potentially due to limited plot-level data and geographic biases in data availability (most data primarily from China; Fig. 1a). Addressing this limitation will require concerted efforts from the scientific community to perform large-scale, networked experiments across continents. Third, our estimates rely on interpolated global datasets with coarse spatiotemporal resolution (Supplementary Table 5), as many plot-level metadata are unavailable. To reduce uncertainties in such datasets, further efforts to enhance data quality and resolution are necessary. Despite these limitations, the comprehensive nature of our study, which incorporates a diverse range of data sources and advanced analytical methods, provides a robust estimation of N fertilizer-induced C pools changes."

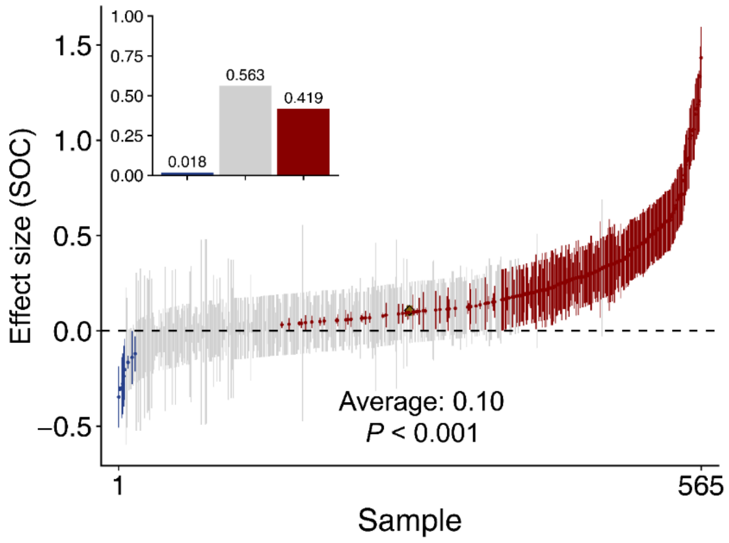

Figure 4. Sample effect sizes of SOC in responding to nitrogen (N) addition.

The heights of bar plots are relative proportions of negative (blue), neutral (grey) and positive (red) (CIs crossing zero) effect sizes, and were standardized, ranging from 0 to 1. Circles and error bars represent average parameter estimates and 95% confidence intervals (CIs) in the linear mixed effects models (two–sided). The vertical dashed line represents the warming effect size = 0.   If the 95% CI did not overlap zero, the effect of warming was statistically significant ($P < 0.05$).

Bergen, K. J., Johnson, P. A., De Hoop, M. V., & Beroza, G. C. (2019). Machine learning for data-driven discovery in solid Earth geoscience. Science, 363(6433), eaau0323. https://doi.org/10.1126/science.aau0323

Han, P., Zhang, W., Wang, G., Sun, W., & Huang, Y. (2016). Changes in soil organic carbon in croplands subjected to fertilizer management: A global meta-analysis. *Scientific Reports*, *6*(1), 27199. https://doi.org/10.1038/srep27199

Li, X. G., Jia, B., Lv, J., Ma, Q., Kuzyakov, Y., & Li, F. (2017). Nitrogen fertilization decreases the decomposition of soil organic matter and plant residues in planted soils. *Soil Biology and Biochemistry*, *112*, 47–55. https://doi.org/10.1016/j.soilbio.2017.04.018

Khan, S. A., Mulvaney, R. L., Ellsworth, T. R., & Boast, C. W. (2007). The Myth of Nitrogen Fertilization for Soil Carbon Sequestration. *Journal of Environmental Quality*, *36*(6), 1821–1832. https://doi.org/10.2134/jeq2007.0099

Poffenbarger, H. J., Barker, D. W., Helmers, M. J., Miguez, F. E., Olk, D. C., Sawyer, J. E., Six, J., & Castellano, M. J. (2017). Maximum soil organic carbon storage in Midwest U.S. cropping systems when crops are optimally nitrogen-fertilized. *PLOS ONE*, *12*(3), e0172293. https://doi.org/10.1371/journal.pone.0172293

Martre, P., Dueri, S., Guarin, J. R., Ewert, F., Webber, H., Calderini, D., Molero, G., Reynolds, M., Miralles, D., Garcia, G., & others. (2024). Global needs for nitrogen fertilizer to improve wheat yield under climate change. *Nature Plants*, *10*(7), 1081–1090. https://doi.org/10.1038/s41477-024-01739-3

Mueller, N. D., Gerber, J. S., Johnston, M., Ray, D. K., Ramankutty, N., & Foley, J. A. (2012).

Closing yield gaps through nutrient and water management. *Nature*, *490*(7419), 254–257. https://doi.org/10.1038/nature11420

Zhao, Y., Wang, M., Hu, S., Zhang, X., Ouyang, Z., Zhang, G., Huang, B., Zhao, S., Wu, J., Xie, D., Zhu, B., Yu, D., Pan, X., Xu, S., & Shi, X. (2018). Economics- and policy-driven organic carbon input enhancement dominates soil organic carbon accumulation in Chinese croplands. Proceedings of the National Academy of Sciences of the United States of America, 115(16), 4045–4050. https://doi.org/10.1073/pnas.1700292114

Rocci, K. S., Lavallee, J. M., Stewart, C. E., & Cotrufo, M. F. (2021). Soil organic carbon response to global environmental change depends on its distribution between mineral-associated and particulate organic matter: A meta-analysis. *Science of The Total Environment*, *793*, 148569. https://doi.org/10.1016/j.scitotenv.2021.148569

Tang, B., Rocci, K. S., Lehmann, A., & Rillig, M. C. (2023). Nitrogen increases soil organic carbon accrual and alters its functionality. *Global Change Biology*, *29*(7), 1971–1983. https://doi.org/10.1111/gcb.16588

Wu, J., Zhang, H., Pan, Y., Cheng, X., Zhang, K., & Liu, G. (2023). Particulate organic carbon is more sensitive to nitrogen addition than mineral-associated organic carbon: A meta-analysis. *Soil and Tillage Research*, *232*, 105770. https://doi.org/10.1016/j.still.2023.105770

Xu, Y., Zhao, Y., Cha, X., Yang, W., Zheng, M., Liu, S., Wang, Y., Cai, A., Han, X., Yang, G., & Ren, C. (2024). Nitrogen addition-driven soil organic carbon stability depends on the fractions of particulate and mineral-associated organic carbon. *Nutrient Cycling in Agroecosystems*, *128*(2), 269–281. https://doi.org/10.1007/s10705-024-10343-y

[B6] Overall, although I see the potential of that paper and dataset to advance our understanding on SOC responses to mineral N fertilization, I am not convinced yet and recommend that major revisions are necessary.

Response: We are very grateful to the Reviewer for their supportive and helpful comments that have informed the further development of our analysis and the overall quality of the manuscript. In doing so, we hope that we have met the concerns of the Reviewer. Please see our responses to the comments listed above and below for detailed modifications.

[B7] l.50-52: I don’t agree with that statement. Not everywhere in the world, an increase in fertilization is possible or helpful to increase crop yields. In many parts, soils are already overfertilized.

Response: We thank the Reviewer for making this important point. We have reworded the sentence as: “Nitrogen (N) fertilization is essential for agricultural productivity to meet the human demand for food [6], but can strongly affect SOC dynamics [7,8].” (Lines 58–60)

[B8] l.79-81: This sentence is not very logical to me. Where is the connection between mineral properties and initial SOC?

Response: We apologize for the lack of clarity. We have revised the sentence as: "Additionally, SOC-rich soils can buffer N-induced acidification due to the cation exchange capacity of soil organic matter, at the cost of increased leaching of base cations (e.g., Ca^{2+} and Mg^{2+}) and greater solubility of hydrolyzed cations (e.g., Fe^{3+} and Al^{3+}) [26]. Changes in MAOC induced by N fertilization may differ between SOC-poor and SOC-rich soils as clay mineralogy is a major control of MAOC accumulation and retention [17,27]." (Lines 85–90)

[B9] l.91: . and is wrong.

Response: Thank you for pointing this out. The suggested revision has been made.

[B10] l.93: MAOC instead of MOAC

Response: Thank you for pointing out this mistake which has been corrected.

[B11] l.181: What did you mean by N fertilization aggregated C limitation?

Response: We thank the Reviewer for making raising this issue. The global average carbon-to-nitrogen (C:N) ratio of soil microbial biomass is approximately 8 (Xu et al., 2013), with an average carbon use efficiency (CUE) of 0.3 and nitrogen use efficiency (NUE) of 0.9 (Mooshammer et al., 2014; Sinsabaugh et al., 2013). Based on threshold element ratios (TER) theory, the global average TER of soil microbial biomass is around 21. However, given that the average soil C:N ratio is 16, or even lower in mineral soils and cultivated lands, soil microorganisms are generally C-limited (Soong et al., 2020). N addition often expands the C demand by microorganisms, thus further increased microbial C limitation (Kamble et al., 2013) according to stoichiometry theory (Bertrand et al., 2019). We have strengthened the discussion on how N fertilization exacerbates C limitation in C-poor soils.

Lines 278–280: "Furthermore, according to EST, when N is abundant in C-poor soils, an imbalance between resource availability and microbial nutrient needs can lead to stoichiometric imbalance [22,24]."

Xu, X., Thornton, P. E., & Post, W. M. (2013). A global analysis of soil microbial biomass carbon, nitrogen and phosphorus in terrestrial ecosystems. *Global Ecology and Biogeography*,

22(6), 737–749. https://doi.org/10.1111/geb.12029
Mooshammer, M., Wanek, W., Hämmerle, I., Fuchslueger, L., Hofhansl, F., Knoltsch, A., Schnecker, J., Takriti, M., Watzka, M., Wild, B., & others. (2014). Adjustment of microbial nitrogen use efficiency to carbon: Nitrogen imbalances regulates soil nitrogen cycling. *Nature Communications*, *5*(1), 3694. https://doi.org/10.1038/ncomms4694
Sinsabaugh, R. L., Manzoni, S., Moorhead, D. L., & Richter, A. (2013). Carbon use efficiency of microbial communities: Stoichiometry, methodology and modelling. *Ecology Letters*, *16*(7), 930–939. https://doi.org/10.1111/ele.12113
Soong, J. L., Fuchslueger, L., Marañon-Jimenez, S., Torn, M. S., Janssens, I. A., Penuelas, J., & Richter, A. (2020). Microbial carbon limitation: The need for integrating microorganisms into our understanding of ecosystem carbon cycling. *Global Change Biology*, *26*(4), 1953–1961. https://doi.org/10.1111/gcb.14962
Kamble, P. N., Rousk, J., Frey, S. D., & Bååth, E. (2013). Bacterial growth and growth-limiting nutrients following chronic nitrogen additions to a hardwood forest soil. *Soil Biology and Biochemistry*, *59*, 32–37. https://doi.org/10.1016/j.soilbio.2012.12.017
Bertrand, I., Viaud, V., Daufresne, T., Pellerin, S., & Recous, S. (2019). Stoichiometry constraints challenge the potential of agroecological practices for the soil C storage. A review. *Agronomy for Sustainable Development*, *39*(6), 54. https://doi.org/10.1007/s13593-019-0599-6

[B12] l. 261: F:B ratio: Why is it increasing with N fertilization? I expected the opposite.

Response: We thank the Reviewer for drawing our attention to this. We have changed “F/B ratio” to “F:B”. Generally, fungi have a higher C assimilation capacity than bacteria and are more effective in forming biomass by metabolizing organic compounds (Herrmann et al., 2014). In our study, N fertilization intensified microbial C limitation in C-poor soils, thus making it more suitable for fungi that decompose recalcitrant carbon to survive (Ye et al., 2019). This is also supported by previous study showing that carbon availability can affect microbial community structure (Fanin et al., 2015). The observed increase in the F:B due to N fertilizer application has also been reported in other field experiments and was further explained (Dai et al., 2017; C. Han et al., 2024).

Herrmann, A. M., Coucheney, E., & Nunan, N. (2014). Isothermal microcalorimetry provides new insight into terrestrial carbon cycling. *Environmental Science & Technology*, *48*(8), 4344–4352. https://doi.org/10.1021/es403941h
Ye, G., Lin, Y., Kuzyakov, Y., Liu, D., Luo, J., Lindsey, S., Wang, W., Fan, J., & Ding, W. (2019). Manure over crop residues increases soil organic matter but decreases microbial necromass relative contribution in upland Ultisols: Results of a 27-year field experiment. *Soil Biology and Biochemistry*, *134*, 15–24. https://doi.org/10.1016/j.soilbio.2019.03.018

Fanin, N., Hättenschwiler, S., Schimann, H., & Fromin, N. (2015). Interactive effects of C, N and P fertilization on soil microbial community structure and function in an A mazonian rain forest. *Functional Ecology*, *29*(1), 140–150. https://doi.org/10.1111/1365-2435.12329

Dai, X., Wang, H., & Fu, X. (2017). Soil microbial community composition and its role in carbon mineralization in long-term fertilization paddy soils. *Science of The Total Environment*, *580*, 556–563. https://doi.org/10.1016/j.scitotenv.2016.11.212

Han, C., Chen, L., Xin, X., Zhou, G., Zhang, C., Ma, D., Li, Y., Ma, L., & Zhang, J. (2024). Long-term fertilization affects microbial necromass accumulation by regulating nutrient and enzymatic stoichiometry in a calcareous Fluvisol. *Applied Soil Ecology*, *194*, 105169. https://doi.org/10.1016/j.apsoil.2023.105169

[B13] l.287: recent advances of which kind?

Response: We thank the Reviewer for pointing this out. Recent studies highlight the critical role of soil enzymes in decomposing various carbon pool components. Numerous papers have shown that enhanced oxidase activity can accelerate the decomposition of carbon with slower turnover rates, such as the MAOC pool (Chen et al., 2018; Hu et al., 2024; Rui et al., 2022). To avoid potential confusion, we removed references to "recent advances" and instead included additional literature citations for clarity.

Lines 309–311: “Third, analysis of extracellular enzymes provides evidence that elevated phenol oxidase and peroxidase activities may increase the potential of microbes to acquire C from SOC pools with slower turnover rates [31,46]. ”

Chen, J., Luo, Y., Van Groenigen, K. J., Hungate, B. A., Cao, J., Zhou, X., & Wang, R. (2018). A keystone microbial enzyme for nitrogen control of soil carbon storage. *Science Advances*, *4*(8), eaaq1689. https://doi.org/10.1126/sciadv.aaq1689

Hu, Q., Zhang, Y., Cao, W., Yang, Y., Hu, Y., He, T., Li, Z., Wang, P., Chen, X., Chen, J., & others. (2024). Legume cover crops sequester more soil organic carbon than non-legume cover crops by stimulating microbial transformations. *Geoderma*, *450*, 117024. https://doi.org/10.1016/j.geoderma.2024.117024

Rui, Y., Jackson, R. D., Cotrufo, M. F., Sanford, G. R., Spiesman, B. J., Deiss, L., Culman, S. W., Liang, C., & Ruark, M. D. (2022). Persistent soil carbon enhanced in Mollisols by well-managed grasslands but not annual grain or dairy forage cropping systems. *Proceedings of the National Academy of Sciences*, *119*(7), e2118931119. https://doi.org/10.1073/pnas.2118931119

[B14] l.323-325: This contradicts state-of-the-art literature. Others either find a relatively stable ratio of POC and MAOC across a wide range of C contents (Begill, Matus) or an increasing proportion of POC with increasing SOC (Cotrufo).

Response: We thank the Reviewer for bringing raising this important issue. Our global predictions are based on extrapolating from the database we constructed. Specifically, the control represents no fertilizer application, while the treatment involves N fertilization. The results reflect the percentage change in POC/MAOC caused by fertilization compared to no fertilizer, rather than the contribution to SOC. To avoid any misunderstanding, we have revised the description in the Results (Lines 235–238). We are grateful to the Reviewer for providing pertinent references, and we have cited them in the revised text (Lines 69–71; Lines 287–288).

Lines 235–238: "N fertilizer application increased POC by 26% in C-poor soils and 22% in C-rich soils, compared to unfertilized soils (Supplementary Fig. 12). However, the MAOC increase induced by fertilization was 7% in C-poor soils and 8% in C-rich soils (Supplementary Fig. 12)."

[B15] L363-366: How large is the actual difference in texture between the two experiments? That is important, because this could also explain the difference in SOC, as well as the dynamic of MAOC and POC.

Response: We thank the Reviewer for stressing the important issue of soil texture with which we agree entirely. The soil textures are all stated in the Field Site Description of the soils at the 4 experimental sites: Siping is silt clay, Yaan is clay loam, Changwu is silt loam and Quzhou is clay loam. As suggested, we further analyzed the mediating role of soil texture in the dynamics of POC and MAOC under N fertilization across the global dryland farmland. Please refer to *Comment B3* for details.

[B16] l.520: this whole section is not explained well enough. How is the global mapping done, which kind of covariate maps are in the background, and where did they come from?

Response: We apologize to the Reviewer for the lack of clarity. We have provided a more detailed description to clarify our approach to global-scale upscaling (lines 595–612). We have also provided detailed information on all the covariates used for upscaling, including climate, soil, and fertilizer variables, along with their respective units, resolutions, and data sources (Supplementary Table 6).

Lines 595–612: "To create a spatially predictive model of the effect of current N

fertilizer on POC and MAOC, we first sampled our prepared stack of environmental covariates at each independent data point within the compiled database. These layers included climatic, soil, and fertilizer use (Supplementary Table 6). Specifically, values of all grids of environmental layers were extracted based on site-specific coordinates or directly from original studies when available. All covariates were resampled and reprojected to a unified pixel grid in EPSG:4326 (WGS84) at five-arcminute spatial resolution. We selected four common and reliable machine-learning models (random forest model, the extreme gradient-boosting model, the light gradient-boosting machine model, and the gradient-boosting decision trees mode) to predict POC and MAOC changes. The data used for training and validation are 80% and 20%, respectively. We used a grid-search procedure, with five-fold cross-validation, to select the best model. The predictive power of the models was measured using three key metrics: the Root Mean Square Error (RMSE), the Mean Absolute Error (MAE), and the goodness of fit, as represented by the coefficient of determination (R^2). The results showed that the gradient-boosting decision trees machine model performed best among all models (characterized by the maximum R^2 and the minimum MAE and MSE; Supplementary Table 5) and was therefore selected to predict the global variations in POC/MAOC. All models were run in Python."

Supplementary Table 6. Summary of covariates used in this study for upscaling our results to a global context.

Group	Covariates	Units	Resolution	Source
Climate	Mean annual temperature (MAT)	℃	30 s	WorldClim, (Fick & Hijmans, 2017)
	Mean annual precipitation (MAP)	mm $year^{-1}$	30 s	
Soil	Soil organic carbon (SOC)	g kg^{-1}	250 m	SoilGrids, (Poggio et al., 2021)
	Total nitrogen (TN)	g kg^{-1}	250 m	
	pH	/	250 m	
	Clay Fraction (Clay)	%	250 m	
	Silt Fraction (Silt)	%	250 m	

Fertilizer	Nitrogen fertilization rate (Ninput)	kg ha^{-1} $year^{-1}$	5 min	PANGAEA, (Zhang et al., 2017)
/	Land use data	/	30 m	(Potapov et al., 2022)

Soil properties from SoilGrids were converted into standardized units based on conversion relationships provided in the official manual to align with our dataset.

Fick, S. E., & Hijmans, R. J. (2017). WorldClim 2: New 1-km spatial resolution climate surfaces for global land areas. *International Journal of Climatology*, *37*(12), 4302–4315. https://doi.org/10.1002/joc.5086

Poggio, L., De Sousa, L. M., Batjes, N. H., Heuvelink, G. B., Kempen, B., Ribeiro, E., & Rossiter, D. (2021). SoilGrids 2.0: Producing soil information for the globe with quantified spatial uncertainty. *Soil*, *7*(1), 217–240. https://doi.org/10.5194/soil-7-217-2021

Zhang, B., Tian, H., Lu, C., Dangal, S. R., Yang, J., & Pan, S. (2017). Global manure nitrogen production and application in cropland during 1860–2014: A 5 arcmin gridded global dataset for Earth system modeling. *Earth System Science Data*, *9*(2), 667–678. https://doi.org/10.5194/essd-9-667-2017

Potapov, P., Turubanova, S., Hansen, M. C., Tyukavina, A., Zalles, V., Khan, A., Song, X.-P., Pickens, A., Shen, Q., & Cortez, J. (2022). Global maps of cropland extent and change show accelerated cropland expansion in the twenty-first century. *Nature Food*, *3*(1), 19–28. https://doi.org/10.1038/s43016-021-00429-z

C. Response to Review #3

[C1] I have enjoyed reviewing your manuscript entitled "Soil organic carbon thresholds control fertilizer effects on carbon accrual in croplands worldwide." Your research presents valuable insights into unraveling the N fertilization effects on soil organic carbon dynamics, which has significant implications for environmental and agricultural management. Overall, I found your paper well-written, scientifically sound, and impactful. The data and analyses you present provide a strong foundation for your conclusions, and your discussion of global implications is well-argued.

Response: We thank the Reviewer for supportive comments and for highlighting the strengths and the novelty of our study.

[C2] L91 ". and"

Response: We have made the revision suggested.

[C3] L172-174. N fertilizer effect on lignin phenols was not significant in C-poor soil (Figure 3a; Supplementary Figure 8). I see the trend, but could you soften the statement or provide the p-value?

Response: We thank the Reviewer for raising this point and apologize for the lack of clarity. We have added the *P*-value to make it clear; it now reads:

Lines 186–188: "N fertilizer increased lignin phenols by 18.4% (P = 0.121) but decreased microbial necromass C in C-poor soils (P < 0.001; Fig. 3a and Supplementary Fig. 8)."

[C4] L187 "…the mean weight diameter (MWD) of aggregates"

Response: We thank the Reviewer for raising this point. The sentence now reads:

Lines 201–202: "We then measured the mean weight diameter (MWD) of aggregates and the content of exchangeable cations to quantify SOC protection by aggregates and minerals, respectively."

[C5] L261 F/B was not defined. (chose between F:B or F/B to refer to the ratios)

Response: Thank you for pointing this out. We have standardized the abbreviations for the fungal-to-bacterial ratio (F:B) throughout the article to enhance clarity.

[C6] L306. I'm confused with what you refer to with the recalcitrant and labile

pools. Aliphaticity and recalcitrance are indexes but do not represent SOC pools. The organic compounds interacting with minerals in the MAOM pools are not necessarily recalcitrant; they could be labile compounds that do not decompose because of mineral protection. Therefore, I'm not sure that the accumulation of SOC is attributed to the "recalcitrant C pool." Aliphaticity and recalcitrance increased in C-rich soil with N addition, but we don't know if these compounds are in POC or MAOC. N could produce a quality change in POC that does not necessarily explain increases in MAOC or SOC.

Response: We thank the Reviewer for raising the point that MAOC may include easily degradable compounds whose breakdown is hindered by mineral protection. We agree with the Reviewer that organic compounds bound to minerals are not inherently related chemically recalcitrant. We have revised the manuscript to refer to stable rather than 'recalcitrant' descriptions to directly link enhanced necromass carbon and mineral protection with MAOC, rather than SOC indexes, as supported by previous studies (Buckeridge et al., 2020; Cotrufo et al., 2013; C. Liang & Zhu, 2021; Liu et al., 2022).

Lines 325–327: "Overall, N-induced MAOC accumulation in C-rich soils mainly resulted from accumulated necromass and increased stabilization on minerals (Figs. 2d and 3a)."

Buckeridge, K. M., Mason, K. E., McNamara, N. P., Ostle, N., Puissant, J., Goodall, T., Griffiths, R. I., Stott, A. W., & Whitaker, J. (2020). Environmental and microbial controls on microbial necromass recycling, an important precursor for soil carbon stabilization. *Communications Earth & Environment*, *1*(1), 36. https://doi.org/10.1038/s43247-020-00031-4

Cotrufo, M. F., Wallenstein, M. D., Boot, C. M., Denef, K., & Paul, E. (2013). The Microbial Efficiency-Matrix Stabilization (MEMS) framework integrates plant litter decomposition with soil organic matter stabilization: Do labile plant inputs form stable soil organic matter? *Global Change Biology*, *19*(4), 988–995. https://doi.org/10.1111/gcb.12113

Liang, C., & Zhu, X. (2021). The soil Microbial Carbon Pump as a new concept for terrestrial carbon sequestration. *Science China Earth Sciences*, *64*(4), 545–558. https://doi.org/10.1007/s11430-020-9705-9

Liu, F., Qin, S., Fang, K., Chen, L., Peng, Y., Smith, P., & Yang, Y. (2022). Divergent changes in particulate and mineral-associated organic carbon upon permafrost thaw. *Nature Communications*, *13*(1), 5073. https://doi.org/10.1038/s41467-022-32681-7

[C7] L305. MAOC could also have plant-derived compounds, not just necromass.

Response: We thank the Reviewer for highlighting this point. We agree that two

pathways (increasing the microbial necromass or protection of low– molecular weight organic compounds from plants) are key C-inputs for MAOC formation (Cotrufo et al., 2013; Lavallee et al., 2020). In our experiment, N fertilization increased the content of microbial necromass C in high-fertility soil while decreasing lignin phenols. Consequently, we attribute the accumulation of MAOC primarily to the increase in necromass C and the subsequent enhancement of mineral protection. We have revised the sentence in question and hope that the meaning is now improved (Lines 325–327).

Cotrufo, M. F., Wallenstein, M. D., Boot, C. M., Denef, K., & Paul, E. (2013). The Microbial Efficiency-Matrix Stabilization (MEMS) framework integrates plant litter decomposition with soil organic matter stabilization: Do labile plant inputs form stable soil organic matter? *Global Change Biology*, *19*(4), 988–995. https://doi.org/10.1111/gcb.12113

Lavallee, J. M., Soong, J. L., & Cotrufo, M. F. (2020). Conceptualizing soil organic matter into particulate and mineral-associated forms to address global change in the 21st century. *Global Change Biology*, *26*(1), 261–273. https://doi.org/10.1111/gcb.14859

[C8] L307. Belowground biomass was not measured, so assessing it is impossible. However, roots and rhizodeposition likely play a role in MAOC formation, not just AGB. Nitrogen fertilization positively impacted AGB, and it is likely that it also increased root biomass (bigger plants, more roots too). It would be good to mention the potential role of roots in your discussion.

Response: We are grateful for the Reviewer's comment, which was also raised by Reviewer#1 *[Comment A2]*). We have now provided root biomass data and highlight the key role of roots in the formation of MAOC in the Discussion section:

Lines 265–266: "In agricultural systems, crop yields are often increased by N fertilization, and both above- and belowground biomass and root-derived C inputs are generally larger [18]."

Lines 302–305: "First, the increase in root biomass and rhizodeposition induced by N fertilizer increased the availability of simple carbohydrates, which are more efficiently utilized to form microbial biomass and subsequently transformed into MAOC [44,45]."

[C9] L821 "…Global map of particulate organic carbon (POC) changes"; "Global map of mineral-associated organic carbon (MAOC) changes".

Response: We thank the Reviewer for pointing out this mistake. We have

revised the phrase according to their suggestion.

[C10] Figure 1. I would flip the color for C-poor and C-rich soils. This is more personal and unnecessary, but for me, it is more intuitive that the green color is associated with higher SOC.

Response: We appreciate the Reviewer's comment. We have updated the color scheme to represent C-rich soils in green and C-poor soils in brown based on Reviewer's comments. Please see the revised Figure 1.

[C11] Figure 1. In panels d and e, the RR of reference (no change) should be 1 – not zero – according to its definition in methods.

Response: We appreciate the Reviewer's comment. We used the natural logarithm of the response ratio (lnRR) as the effect size in this study. We apologize for the previous omission of part of the calculation formula for lnRR in the Materials and Methods section, which has now been included. The effect was significant if the 95% CI of lnRR did not overlap with zero.

Lines 616–619: "The effects of N fertilization were estimated based on the natural log-transformed response ratio (*RR*) [69], confidence intervals (CIs) on the weighted effect size were generated using boot-strapping (999 iterations, function confint, package metafor):"

$$lnRR = \ln\left(\overline{X_t}/\overline{X_c}\right) = \ln(X_t) - \ln(X_c) \quad (1)$$

[C12] Figure 1. In panels b and c, the linear fitted models at two sides of the threshold ignore the continuum nature of the initial SOC. Given that there are different models, an abrupt change in the estimation occurs from one side to the other side of the threshold. Therefore, I think it is not a good way to assess differences between C-poor and C-rich soils. In addition, using bootstrapping to assess the slopes of the model assumes independence between groups. However, some data in these groups could come from the same study, and this independence assumption is violated. The generalized additive model is more appropriate. (The caption does not explain the difference between solid and dashed blue lines).

Response: We appreciate the Reviewer's observation regarding the potential issue of ignoring the continuity of initial SOC in our linear fits before and after the threshold value. To address this, we would like to further clarify our

approach regarding the threshold analysis. We used the Akaike information criterion (AIC) in two steps to identify the most suitable model that describing the relationship between initial SOC (x) and effect sizes of POC/MAOC (y) (Berdugo et al., 2020). First, we compared linear and nonlinear models, including quadratic and generalized additive models (GAM), to evaluate x–y relationships. By fitting the data with both linear and nonlinear models, we found that the nonlinear model provided a better fit to the data, suggesting the presence of a C threshold. This finding offers valuable insights for informed management practices.

We further assessed whether C functional component responses along initial SOC gradients align with an initial C threshold following method of (Gross et al., 2024). We employed threshold models to identify breakpoints by partitioning observations based on initial SOC values, applying different fitting functions on either side by using the R package `chngpt` (Fong et al., 2017). The 6 models included a conventional linear model (GAM), step, segmented, stegmented, hinge 12, and hinge 22 models were further used to optimal threshold model. Model performance was evaluated using AIC and BIC, with the model showing the lowest BIC deemed the best fit. The stegmented model emerged as the best fit (Supplementary Table 1), consistent with previous analyses.

To test whether the identified threshold significantly impacted the slope and/or intercept of the fitted regressions, we bootstrapped linear regressions on both sides of the threshold and used a Mann–Whitney U-test to compare slopes. In most cases with better nonlinear fits, slopes differed significantly across the threshold (Figs. 1b and c). The Reviewer noted that some data might originate from the same study, making bootstrapping unsuitable. We agreed and instead applied a mixed linear model (*lme4* package), treating the study as a random effect. The results are consistent with those obtained by Mann-Whitney U-test. ($P < 0.001$ for POC; $P < 0.001$ for MAOC).

We also acknowledge the Reviewers' suggestion to use the GAM for nonlinear fitting. The black long-dashed lines represent the smoothed trend fitted by a generalized additive model and blue solid lines represent the linear fits at both sides of each threshold, as described in the revised figure legend (Figs. 2b, c). Furthermore, we have supplemented the heterogeneity analysis

to assess significant difference in effect size between before and after the threshold revealed (Figs. 2b, c). The results further support the reliability of the identified threshold in the previous analysis. We have also revised the description of the threshold analysis method to enhance its clarity and ensure its reliability (Lines 626–641).

Berdugo, M., Delgado-Baquerizo, M., Soliveres, S., Hernández-Clemente, R., Zhao, Y., Gaitán, J. J., Gross, N., Saiz, H., Maire, V., Lehmann, A., Rillig, M. C., Solé, R. V., & Maestre, F. T. (2020). Global ecosystem thresholds driven by aridity. *Science*, *367*(6479), 787–790. https://doi.org/10.1126/science.aay5958

Gross, N., Maestre, F. T., Liancourt, P., Berdugo, M., Martin, R., Gozalo, B., Ochoa, V., Delgado-Baquerizo, M., Maire, V., Saiz, H., Soliveres, S., Valencia, E., Eldridge, D. J., Guirado, E., Jabot, F., Asensio, S., Gaitán, J. J., García-Gómez, M., Martínez, P., … Le Bagousse-Pinguet, Y. (2024). Unforeseen plant phenotypic diversity in a dry and grazed world. *Nature*, *632*(8026), 808–814. https://doi.org/10.1038/s41586-024-07731-3

Fong, Y., Huang, Y., Gilbert, P. B., & Permar, S. R. (2017). chngpt: Threshold regression model estimation and inference. *BMC Bioinformatics*, *18*, 1–7. https://doi.org/10.1186/s12859-017-1863-x

[C13] Figure 2 (b,c) This figure has too much information, and the analysis shown in panels b and c is not intuitive. I suggest trying another way to highlight this correlation's main findings and may leave these panels for the supplementary material. In panel c, the meaning of color dots is not described, and the underscore in variable names should be removed.

Response: We are grateful for the Reviewer’s comment. We selected partial correlation analysis over other methods (such as Random Forest, Variance Partitioning Analysis, etc.) to evaluate variable importance because partial correlation allows us to assess the effect of each factor on the target variable while controlling for other variables within each category. Following the Reviewer’s suggestion, we have removed insignificant-correlation grid fill colors to make our results more clearly (Figs. 2b, c). In addition, we have also made the corresponding modifications to panel c.

[C14] Figure 4. I would explore a different legend scale to see clearer the spatial patterns.

Response: We thank the Reviewer for their suggestion, and have made revisions in response to their comments. First, we examined the data’s frequency distribution, identified the interval with the highest frequency, and

subdivided the data in increments of 0.5 to enhance the color gradient. Second, we adjusted the aspect ratio to improve visual appeal. Third, we ensured the plot area fully occupies the drawing space by removing any blank margins around the axes. Finally, we reviewed official mapping examples and replaced the colors with more distinct and easily distinguishable options. Please see Fig. 4.

[C15] Is it possible to know which types of crops are represented in the meta-analysis? Are they all annual crops?

Response: We appreciate the Reviewer’s comment. We checked our database and found that maize and wheat were the predominant crop types (62.7% and 50.7%, respectively—most studies were done on two crops, so this accounts for more than 100%). Additionally, we categorized the crops into annual and perennial types, discovering that only 2 studies mentioned perennial crops—milk vetch and oat clover—accounting for just 1.69% of the total. We believe that this limited presence of perennial crops will not confuse our findings.

Response to reviewer comments

A. Response to Reviewer #1:

[A1] The authors did a good job, and they addressed all of my concerns, I have no more comments.

Response: Thank you very much for your kind support and for taking the time to review our work again. We greatly appreciate your positive feedback.

B. Response to Reviewer #2:

[B1] The authors took a great effort to revise the manuscript, which is now much improved. The analytical and statistical part of that manuscript is very strong, my remaining points are mostly related to the text. I believe that it can be ready for publication after some moderate revisions.

Response: Thank you very much for your kind support and for taking the time to review our work again. We greatly appreciate your positive feedback.

[B2] l.44-46: I have the feeling that it should be toned down a bit. On average, POC and MAOC are promoted in all cases. There just seems to be a higher tendency of MAOC vs. POC formation in high C soils. This should be better reflected here.

Response: Thanks for your constructive comments and careful reading. We have reworded the sentence as:

Lines 45-49: "In SOC-poor soils (< 15 g C kg^{-1}), N fertilizer increases plant-derived C inputs and promotes soil aggregation, favouring POC accumulation. Conversely, in SOC-rich soils, N fertilizer stimulates microbial metabolic efficiency, leading to larger necromass production and stabilization by mineral protection, observed as more pronounced MAOC accrual."

[B3] l.70: increases of plant C inputs.

Response: Thanks for pointing this out. The suggested revision has been made (Line 72).

[B4] l.76-85: I have the feeling that the EST part and particularly the difference between C-poor and C-rich soils still needs some better explanation here. How and why does the stoichiometric imbalance (C limitation) occur in a soil with 1.4% C and not in a soil with 1.6% C. When it is so driven by stoichiometry, then it should not only be the amount of C in the soil, but e.g. also the amount of N that is added to the soil, right? It should be crystal-clear, why such a sharp threshold value is mechanistically justified.

Response: We appreciate the Reviewer's careful consideration and comments.

Ecological stoichiometric theory unifies ecology with elemental ratios and stoichiometric invariance that can predict nutrient retention and biomass production of microorganisms (Allen et al., 2009; Sinsabaugh et al., 2012). N fertilizer supplies readily available nutrients for microorganisms but may lead to changes in C limitation depending on SOC contents (Traoré et al., 2016; Zheng et al., 2020; Liu et al., 2023). Our study identified a threshold of 1.5% SOC modifies the response of POC and MAOC to N fertilizer, resonating with other studies that focus on the influence of SOC threshold on microbial metabolism. Based on a global meta-analysis, Cui et al (2021) proposed a 1% SOC threshold where microbial metabolism transitions from energy limitation to nutrient limitation. Clayton et al. (2021) reported a threshold of 1% SOC below which microorganisms are more C-limited and exhibit reduced metabolic efficiency, as evidenced by elevated qCO_2 values; above 1% SOC, microorganisms transition to a new equilibrium and N becomes the limiting factor for growth, enabling more efficient C acquisition. We have expanded the discussion on the impact of initial SOC on microbial metabolism (Lines 319-326).

Critically, our study addresses how the response of C pools to N fertilization is affected by SOC levels. While EST is undoubtably important, the SOC threshold in our study but is associated with multiple potential factors, including aggregate stability, mineral properties, and edaphic conditions. By applying systematic research and considering diverse viewpoints, we probe the mechanisms underlying the formation of SOC functional components and how they are moderated by N fertilizer and initial SOC contents by systematically analyzing the sources, transformation processes, and stability of these components. Therefore, we have deepened the understanding of how initial SOC content influences the response of POC and MAOC pools to N fertilizer through direct effects on microbial metabolism influenced by stoichiometric balance and indirect effects on soil physicochemical and mineralogical properties in Introduction section (Lines 78-104).

We also fully agree with the Reviewer's point that microbial stoichiometric balance is influenced not only by C content but also by the amount of N added. Several independent studies have demonstrated that the effects of N fertilizer on soil microbial metabolism (Fierer et al., 2012; Feng et al., 2022), composition (Fierer et al., 2012), and biomass (Yue et al., 2017) are not influenced by the addition rate and exhibit a consistent trend. However, one study found that the impact of N addition on microorganisms became more significant as both the rate and duration of addition increased (Zhang et al., 2018). Therefore, we expanded the discussion on the impact of N fertilizer application rate on microorganisms (Lines 322-326).

Sinsabaugh, R. L., & Shah, J. J. F. (2012). Ecoenzymatic stoichiometry and ecological theory. *Annual Review of Ecology, Evolution, and Systematics*, *43*(1), 313–343. https://doi.org/10.1146/annurev-ecolsys-071112-124414

Allen, A. P., & Gillooly, J. F. (2009). Towards an integration of ecological stoichiometry and the metabolic theory of ecology to better understand nutrient cycling. *Ecology Letters*, *12*(5), 369–384. https://doi.org/10.1111/j.1461-0248.2009.01302.x

Traoré, O. Y. A., Kiba, D. I., Arnold, M. C., Fliessbach, A., Oberholzer, H. R., Nacro, H. B., Lompo, F., Oberson, A., Frossard, E., & Bünemann, E. K. (2016). Fertilization practices alter microbial nutrient limitations after alleviation of carbon limitation in a Ferric Acrisol. *Biology and Fertility of Soils*, *52*(2), 177–189. https://doi.org/10.1007/s00374-015-1061-9

Zheng, L., Chen, H., Wang, Y., Mao, Q., Zheng, M., Su, Y., Xiao, K., Wang, K., & Li, D. (2020). Responses of soil microbial resource limitation to multiple fertilization strategies. *Soil and Tillage Research*, *196*, 104474. https://doi.org/10.1016/j.still.2019.104474

Liu, J., Qiu, T., Peñuelas, J., Sardans, J., Tan, W., Wei, X., Cui, Y., Cui, Q., Wu, C., Liu, L., Zhou, B., He, H., & Fang, L. (2023). Crop residue return sustains global soil ecological stoichiometry balance. *Global Change Biology*, *29*(8), 2203–2226. https://doi.org/10.1111/gcb.16584

Clayton, J., Lemanski, K., & Bonkowski, M. (2021). Shifts in soil microbial stoichiometry and metabolic quotient provide evidence for a critical tipping point at 1% soil organic carbon in an agricultural post-mining chronosequence. *Biology and Fertility of Soils*, *57*(3), 435–446. https://doi.org/10.1007/s00374-020-01532-2

Cui, Y., Moorhead, D. L., Guo, X., Peng, S., Wang, Y., Zhang, X., & Fang, L. (2021). Stoichiometric models of microbial metabolic limitation in soil systems. *Global Ecology and Biogeography*, *30*(11), 2297–2311. https://doi.org/10.1111/geb.13378

Feng, X., Qin, S., Zhang, D., Chen, P., Hu, J., Wang, G., Liu, Y., Wei, B., Li, Q., Yang, Y., & Chen, L. (2022). Nitrogen input enhances microbial carbon use efficiency by altering plant–microbe–mineral interactions. *Global Change Biology*, *28*(16), 4845–4860. https://doi.org/10.1111/gcb.16229

Fierer, N., Lauber, C. L., Ramirez, K. S., Zaneveld, J., Bradford, M. A., & Knight, R. (2012). Comparative metagenomic, phylogenetic and physiological analyses of soil microbial communities across nitrogen gradients. *The ISME Journal*, *6*(5), 1007–1017. https://doi.org/10.1038/ismej.2011.159

Yue, K., Fornara, D. A., Yang, W., Peng, Y., Li, Z., Wu, F., & Peng, C. (2017). Effects of three global change drivers on terrestrial C:N:P stoichiometry: A global synthesis. *Global Change Biology*, *23*(6), 2450–2463. https://doi.org/10.1111/gcb.13569

Zhang, T., Chen, H. Y. H., & Ruan, H. (2018). Global negative effects of nitrogen deposition on soil microbes. *The ISME Journal*, *12*(7), 1817–1825. https://doi.org/10.1038/s41396-018-0096-y

[B5] l.88-89: In the last round I asked for the inclusion of texture, but this is not the way it should be done. This sentence makes no sense.

Response: Thank you for pointing this out. We apologize for the confusion. We have supplemented progress of influence of soil texture on SOC pools according to Reviewer's comments.

Lines 94-98: "Compared to finer-textured (clayey) soils, coarse-textured (sandy) soils tend to be C-poor with smaller CEC and thus poorer buffering capacity against cation leaching caused by N fertilizer use [28]. Variations in mineral cation chemistry influence adsorption and co-precipitation reactions with organic compounds in soils, including those derived from microbial necromass, causing variation in the effect of N fertilizer on MAOC formation [29,30]."

[B6] l.298; maybe call it "a particularly positive effect" to clarify that both fractions in both soils were positively affected by N fertilization (at least in the literature dataset).

Response: We appreciate the Reviewer's insightful suggestion. The sentence now reads:

Lines 308-309: "Here, we observed that N fertilization had particularly positive effect on MAOC in C-rich soils (Figs. 2c, d; Supplementary Fig. 6)."

[B7] l.320-321: Don't make it sound too general, here you are writing about your experiments.

Response: We appreciate the Reviewer's insightful suggestion. We have revised the sentence to clearly reflect that the observed effects are based on our experimental data, rather than a generalized statement. Specifically, we highlight the C-rich soils from SP and YA, emphasizing the larger clay and silt fractions and their direct influence on water-holding capacity, microbial activity, and MAOC accumulation. This revision ensures that the description of our findings is more precise and rooted in the experimental context.

Lines 337-341: "Our study demonstrates that C-rich soils that contain larger clay and silt fractions (such as those from SP and YA) with enhanced water-holding capacity support microbial biomass production and thereby microbial necromass formation [52]. The large surface area of fine soil particles provides more mineral surfaces and reactive sites to stabilize microbial-derived C and promote MAOC accumulation [43,53]."

[B8] l.255: long-term filed networking experiments? What does that mean?

Response: We apologize for any confusion caused by the lack of clarity. In our study, the term "long-term field networking experiments" refers to four long-term field experiments involving N fertilizer, conducted across various grain production regions in China. Hence, we replaced "long-term field networking experiments" with "four field experiments" for clarity and precision (Line 266).

[B9] l.301: This indicates a shift… Grammar in this sentence is not correct.

Response: We thank the Reviewer for pointing out the grammatical issue. The sentence now reads:

Lines 312-313: “This indicates a shift in the trade-off between labile and stable SOC pools under N fertilization, which can be due to multiple overlapping mechanisms.”

[B10] l.337-340: It could also be mentioned that C sequestration via N fertilization is also not a climate mitigation measure, because N fixation requires a lot of energy and N_2O emissions are also strongly increased. The overall GHG budget of a fertilized soil is still less positive than that of an unfertilized soil. This should be briefly mentioned here as well.

Response: We appreciate the Reviewer’s valuable suggestion. We have added the relevant description:

Lines 359-363: “For instance, the benefits of increasing the MAOC sink may be partly offset by increased N_2O emissions, fossil energy and the associated greenhouse gas emissions during fertilizer manufacture [58,59]. Considering the greenhouse gas emissions throughout the entire life cycle of N fertilizer, the global warming potential of fertilized soil remains greater than that of unfertilized soil [59].”

[B11] l.383: Again, the term predominantly is somewhat to strong. In the large literature dataset, the difference is not so large, so I would rather call it “a more pronounced accumulation of POC…” or similar.

Response: We appreciate the Reviewer’s valuable comment. We have revised the sentence as follows:

Lines 405-408: “The dichotomy in SOC accumulation mechanisms we have uncovered – more pronounced POC accumulation in C-poor soils versus MAOC accrual in C-rich conditions - challenges and extends traditional notions of N fertilization impacts.”

C. Response to Reviewer #3:

[C1] The authors responded satisfactorily to my reviews and provided an improved version. My recommendation is to accept the manuscript.

Response: We sincerely thank the reviewer for their positive feedback. We are pleased that our revisions have addressed the concerns, and we appreciate the recommendation for acceptance of the manuscript.
